# Fighting Hypoxia to Improve PDT

**DOI:** 10.3390/ph12040163

**Published:** 2019-10-30

**Authors:** Ludivine Larue, Bauyrzhan Myrzakhmetov, Amina Ben-Mihoub, Albert Moussaron, Noémie Thomas, Philippe Arnoux, Francis Baros, Régis Vanderesse, Samir Acherar, Céline Frochot

**Affiliations:** 1 Laboratoire Réactions et Génie des Procédés (LRGP), UMR 7274, CNRS, Université de Lorraine, 54000 Nancy, France; ludivine.larue@univ-lorraine.fr (L.L.); albert.moussaron@univ-lorraine.fr (A.M.); philippe.arnoux@univ-lorraine.fr (P.A.); francis.baros@univ-lorraine.fr (F.B.); 2 M.Kh. Dulaty Taraz State University, Taraz 080012, Kazakhstan; baur_86_86@mail.ru; 3 Laboratoire de Chimie Physique Macromoléculaire (LCPM), UMR 7375, CNRS, Université de Lorraine, 54000 Nancy, France; amina.ben-mihoub@univ-lorraine.fr (A.B.-M.); regis.vanderesse@univ-lorraine.fr (R.V.); samir.acherar@univ-lorraine.fr (S.A.); 4 Biologie, Signaux et Systèmes en Cancérologie et Neurosciences, CRAN, UMR 7039, Université de Lorraine, CNRS, 54000 Nancy, France; noemie.thomas@univ-lorraine.fr

**Keywords:** PDT, oxygen, hypoxia

## Abstract

Photodynamic therapy (PDT) has drawn great interest in recent years mainly due to its low side effects and few drug resistances. Nevertheless, one of the issues of PDT is the need for oxygen to induce a photodynamic effect. Tumours often have low oxygen concentrations, related to the abnormal structure of the microvessels leading to an ineffective blood distribution. Moreover, PDT consumes O_2_. In order to improve the oxygenation of tumour or decrease hypoxia, different strategies are developed and are described in this review: (1) The use of O_2_ vehicle; (2) the modification of the tumour microenvironment (TME); (3) combining other therapies with PDT; (4) hypoxia-independent PDT; (5) hypoxia-dependent PDT and (6) fractional PDT.

## 1. Introduction

In the case of cancer, tissue hypoxia is a key feature of virtually all solid tumours [1]. The hypoxic adaptation, which is recognized as a source of therapeutic failure in clinical oncology [2] is critical for tumour expansion, invasion and metastasis, and some studies aim to target hypoxia for enhanced cancer therapy [3,4]. In vivo, reduced oxygenation induces a lack of nutrients and growth factors, increased acidosis and production of products coming from the close necrotic area. This leads to alteration of cells and cell-matrix interaction and even sometimes to cell death. Nevertheless, some tumor cells survive and these “hypoxic” cells become very aggressive and induce a local tumor relapse [5]. It is necessary for a treatment to be efficient to detect and control oxygenation in the tissue. Photodynamic therapy (PDT) has been applied to cure many diseases such as AMD, psoriasis, Barrett’s esophagus, and various malignant cancers for over a decade [6]. PDT is a simple and efficient approach relying on irradiation to move a photosensitizer (PS) from the ground state to a singlet excited state and then to a triplet excited state. This excited PS can react with the surrounding medium to produce Reactive Oxygen Species (ROS) such as superoxide anion (O^2−•^) and hydroxyl radical (HO^•^) or transfer its energy to triplet oxygen to generate singlet oxygen (^1^O_2_). Success of PDT requires that the PS incorporate preferentially into the tumor cell, that the number of photons be correctly evaluated in order to excite a majority of the PS molecules, and that there be enough O_2_ to induce the photo-oxidation process [7]. Indeed, PDT, like radiotherapy, suffers from a severe drawback: the need for O_2_ to be efficient. Therefore, PDT displays a low efficiency in hypoxia or when hypoxia occurs due to the consumption of molecular O_2_ by the PS, leading to PDT resistance. When the oxygen concentration in the medium is not high enough, the photodamage is reduced or even no photodynamic reaction occurs at all [8]. These spare hypoxic cells can induce the regrowth of the tumor. Hypoxia occurring during PDT has been studied by several teams last years [9,10,11]. Bush et al. for example used in 2000 oxygen-sensitive electrodes during PDT treatment in vivo [10]. The same team also showed that the spatial distribution of hypoxia during PDT treatment can influence the balance between destruction of the tumor and damage of the neovessels [12]. Several approaches have been described during the last decades to overcome hypoxia in PDT. In this review, we chose to divide them onto six strategies developed in the literature to fight hypoxia for an enhancement of PDT: (1) The use of O_2_ vehicles; (2) the modification of the tumour microenvironment (TME); (3) combining other therapies with PDT; (4) hypoxia-independent PDT; (5) hypoxia-dependent PDT and (6) fractional PDT. 

## 2. O_2_ vehicles

Various clinical applications (i.e. radio-, chemo- and phototherapies) involve the presence of O_2_ in the neighbourhood of the tumours to be treated, but hypoxia can lead to a poor treatment efficiency with enhanced malignant cell survival, increased angiogenesis and metastasis. The supply of O_2_ is therefore a major asset and a new modality for many disease interventions [13,14]. In fact, the increase of the arterial and capillary O_2_ pressure (pO_2_) improves the transport of dissolved O_2_ into the tissue. 

### 2.1. Hyperoxygenation/Hyperbaric Oxygenation (HBO)

Hyperoxic vasodilatation in healthy tissue makes way to vasoconstriction in hypoxic tissues, so one idea is to perform hyper-oxygenation during PDT of pre-existing hypoxic cells and to compensate the O_2_ depletion. This so-called hyperbaric oxygenation (HBO) can be realized in several ways, which will be developed in this section. One way is to provide additional O_2_ in the areas to be treated by the addition of ^1^O_2_ itself, H_2_O_2_, or other molecules. Another way is to favour the formation of ROS at the expense of ^1^O_2_ by switching the photochemistry of PSs from a type II to a type I mechanism [15,16]. Some other interesting reviews on this topic can help in understanding the history, the notion of hypoxia and the broad scope of HBO [17,18,19]. 

Hjelde et al. in 2005 [20] compared the in vitro PDT efficiency under normoxic or hyperoxic exposure on AY27 tumour cells, human colon adenocarcinoma WiDr and SW840 cells. The cultured cancer cells were incubated with 5-ALA and irradiated (435 nm, 0.5 and 30 min, 12.9 mW∙cm^−2^), which matched a total light dose of 0.387 and 23.22 J∙cm^−2^ respectively. After PDT treatment under normoxia (21 kPa O_2_) and hyperoxia (100, 200, 300, or 400 kPa), the cells’ survival was evaluated and surprisingly, showed no significant difference between normoxia and HBO. However, the three cell lines exhibited very different sensitivities to PDT in normoxic conditions: At 21 kPa, and within 5 min illumination, about 90% of WiDr and SW840 cells were killed, while only 20–25% of AY27 cells were destroyed and an illumination for up to 30 min was necessary to attain a similar result. 

Mei et al. in 2019 [21] used 5-ALA with or without HBO for PDT treatment of human squamous carcinoma (A431 cell line) in vitro. For HBO assays, the cells were flushed by a mixture 98% O_2_ plus 2% CO_2_ (0.25 MPa) for 1 h daily before addition of 5-ALA. Both treatments, after irradiation (530 nm, 5 J∙cm^−2^, 8 days) indicated: (a) a decrease of cell proliferation as a function of a concentration gradient (0, 0.1, 0.3, 1, and 3 mM ALA), (b) a synergistic effect of the combination ALA-HBO, especially at 1 and 3 mM ALA with 30–35% cell viability. Western blot analysis allowed the detection of apoptosis-related protein Bax, Bcl-2 and caspase 3. The data suggested synergistically increased levels of the proteins Bax and caspase, but a decrease for Bcl-2 in A431 cells, evidencing a mitochondria-intrinsic pathway cell apoptosis. Furthermore, it has been shown that the dual ALA-HBO treatment reduced ROS production in A431 cells and favoured autophagy. It can be considered that ALA-HBO could be used in vivo for the treatment of human squamous carcinomas.

Chen et al. [22,23] used hyper-oxygenation in vivo in mammary carcinoma tumours implanted into C3H mice (6–8 mm). Tumours were treated by Photofrin and illuminated at 630 nm (200 J∙cm^−2^, 150 mW∙cm^−2^). Hyperbaric conditions were attained when the mice were under a 3-atmosphere pressure. Under normal conditions (1 atm and normoxia) about 20% of the tumour disappeared after 60 days. In normobaric conditions (NBO, 100% O_2_), this percentage increase to 80 %. In hyperbaric conditions (HBO, 100% O_2_), it was only 60 %. Reduction of light fluence to 75 mW∙cm^−2^ combined with NBO treatment led to a 70% tumour cure. The relative negative effect of HBO was perhaps due to a reduced cardiac outcome. The same team used also an atmospheric composition of 95% O_2_ and 5% carbon dioxide (carbogen) [24]. They did not observe any effects after decreasing the fluence. In all cases (HBO, NBO or carbogen) the rate of cellular death was doubled, compared to normal atmospheric conditions. In 2003, they [25] used the same protocol as previously, but measured the O_2_ concentration in tumour in vivo with the Oxylite system. First, only room air (RA) and NBO conditions were used. Table 1 shows the important effect of NBO on the survival rate of tumours after different times (as a tumour fraction).

**Figure 1:**
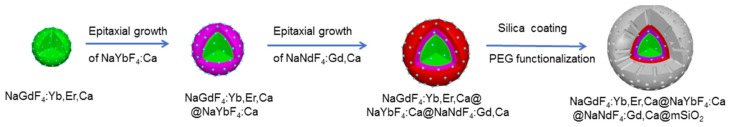
Schematic illustration for the synthesis of NaGdF4:Yb,Er,Ca@NaYbF4:Ca@NaNdF4:Gd,Ca @mSiO2 NPs. Reprinted from [26] with permission from the American Chemical Society, Copyright 2018.

Similar results as previously were obtained using carbogen and HBO conditions. Li et al. [26] used also hyperbaric conditions with upconversion nanoparticles (UCNPs). They synthesized a core@shell NPs NaGdF_4_:Yb,Er,Ca@NaYbF_4_:Ca, with an upconversion core which can transfer its energy to Rose Bengal, and produce ROS under 808 nm laser excitation (Figure 1). It was demonstrated that PDT assisted by HBO led to strong depletion of collagen fibre in the extracellular matrix, with a reduction of hypoxia and a simultaneous enhancement of NP levels in the tumour blood vessels.

In vivo experiments were performed on xenograft 4T1 cells (murine breast tumour cell line) tumour-transplanted mice mouse model. Fluorescence in tumour was observed 6 h after NP administration, up to 48 h and disappeared progressively from the tumour. Consequently, a second treatment (16 min of irradiation with an 808 nm laser, 0.75 W∙cm^−2^) was administered 48 h after the first one. In vitro, 4T1 cell viability was measured to about 75% under hypoxia, about 35% in normoxia and 20% in HBO conditions. In vivo, the tumour weight, initially 0.5 g, decreased to about 0.1 g with UCNP in HBO conditions.

Maier et al. [27] conducted a clinical pilot study to treat patients with advanced oesophageal carcinoma in 2000. They used HpD and illumination (630 nm, 300 J∙cm^−2^), 14 patients were treated by PDT and 17 with PDT under HBO (2 absolute atm pressures). A difference was observed between the two groups, with a median overall survival for the PDT group of 7.0 months (12-months survival rate was 28.6%) and 12 months for the PDT/HBO group (the 12-months survival rate was 41.2% for the PDT/HBO group). The same team published in 2000 another study for the treatment of advanced carcinoma of the cardia and oesophagus [28]. In the same conditions (vide supra), 23 patients were treated by PDT and 29 with PDT/HBO. The mean survival time for the PDT group was 8.7 months and for the PDT/HBO group 13.8 months. This experience was re-conducted the same year on 31 patients for PDT alone and 44 patients for PDT/HBO for the treatment of tumour at the oesophago-gastric junction [29]. A median overall survival of 7 and 12 months was observed, respectively. In order to avoid the prolonged photosensitivity of the skin of HpD, the authors evaluated the efficacy of Photosan^®^ and ALA on patients with oesophageal cancer [30,31]. As expected, polyhematoporphyrin seemed to be more effective than ALA in terms of reduction of dysphagia, tumour stenosis and Karnovsky performance status. A questionnaire of the patients indicated a best comfort for oral administration of ALA than intravenous injection of Photosan^®^ and no complication or sunburn occurred for either treatment. Such a comparison has been made on lung cancer and more particularly in the treatment of advanced malignant bronchogenic stenosis [32,33] and no definitive conclusions have been drawn. At the best of our knowledge, no further investigations in the field of HBO have been undertaken by this team.

In 2001, Schouwink et al. [34] conducted preclinical studies for optimization of PDT by *m*-THPC in female BALB/c mice bearing human mesothelioma (H-MESO1) tumour. To overcome the PDT-induced O_2_ deficit, they proposed a preliminary treatment by nicotinamide (300 mg∙kg^−1^) and/or carbogen (i.e. a mixture 5% CO_2_ and 95% O_2_) 30 min and/or 5 min before illumination, respectively. In a first series, they studied the influence of nicotinamide injection and/or carbogen breathing on the mean time of regrowth of tumour from 2 to 5 mm diameter. The mean time was found to be about 42 days in hypoxic conditions and 17 days in hyperbaric conditions. They analysed also the mean median pO_2_ and they observed no significant increase of oxygenation with nicotinamide alone (17.6 mm Hg to be compared to a control value of 12.6 mm Hg), but a pO_2_ of 24.8 mm Hg for nicotinamide plus carbogen. The efficiency of PDT by *m*-THPC (0.15 mg∙kg^−1^, 100 mW∙cm^−2^) was then discussed and mice survival (after 24 h post PS-injection and illumination) reached 11% for PDT alone and 58% for PDT and carbogen, the nicotinamide bringing almost no improvement. Table 2 lists recent articles involving HBO as O_2_ vehicle.

### 2.2. Red Blood Cells (RBC) or Hemoglobin (Hb)

A new optical method based on in vivo laser-induced photodissociation of blood oxyhemoglobin have been proposed by Asimov et al. [35] to solve the hypoxia problem in malignant tissue and to stimulate the aerobic metabolism of cells. The kinetics of laser-induced oxygenation of cutaneous tissue dependence on the laser-irradiation time was investigated. It was found that upon He-Ne laser irradiation (632 nm, 225 W∙m^−2^) of the cutaneous blood vessels, a local increase in the O_2_ concentration in the irradiated region was observed. Based on the obtained results, the proposed optical method for local hypoxia elimination in malignant tissues may be a good key to increase the efficiency of the different therapeutic procedure in oncology.

**Figure 2:**
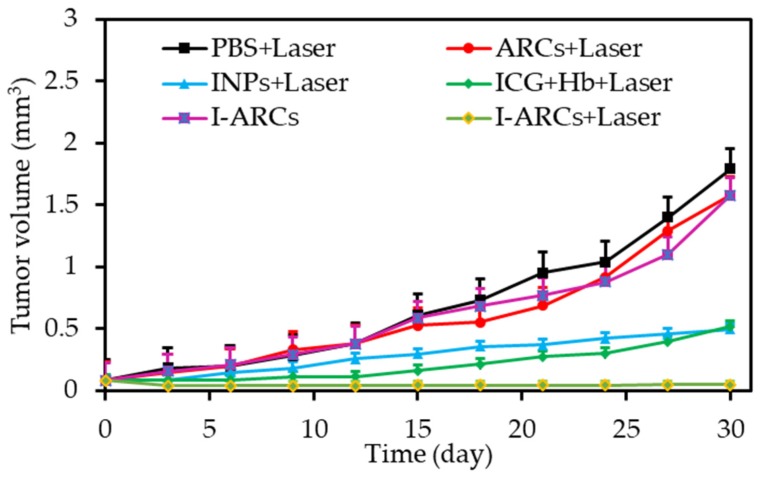
MCF-7 tumour growth curves of different groups after treatments. * *p* < 0.05, ** *p* < 0.01. All injectants were oxygenated before experiments. Adapted from Luo et al. [36].

Luo et al. [36] developed stable nanosize artificial red cells denoted as I-ARCs by loading complexes of indocyanine green (ICG) and O_2_ carrier (hemoglobin, Hb) to incorporate an O_2_ supply and monitor the PDT process. The in vitro studies of I-ARCs compared to deoxy-I-ARCs and ICG NPs (INPs) were performed on MCF-7 human breast cancer cell line under NIR laser irradiation (100 mW∙cm^−2^, 808 nm, 5 min). I-ARCs showed massive generation of ROS (9.5 times stronger than INPs) and 10.7-times higher formation of ferryl-Hb than deoxy-I-ARCs. I-ARCs presented the best phototoxic effects against MCF-7, with a cell viability of 8.9%. The authors explained the remarkable phototoxicity in I-ARCs by the enhanced both ROS production and ferryl-Hb generation. Similarly, the result of PDT-treated mice bearing MCF-7 tumours obtained with I-ARCs was the best compared to the other systems (Figure 2). 

Two years later, the same team [37] designed a protein hybridization approach by developing a bioinspired hybrid protein O_2_ nanocarrier with encapsulated chlorin e6 (Ce6) (C@HPOC) via intermolecular disulfide conjugations for O_2_-augmented immunogenic PDT against tumour growth and metastasis. In vitro ^1^O_2_ production ability of C@HPOC was investigated in 4T1 tumour cells and compared to that of Ce6 and C@HSA. It was found that under laser irradiation (600 nm, 0.1 W∙cm^−2^, 2 min) C@HPOC significantly enhanced the ^1^O_2_ levels compared to the other systems. The PDT treatment revealed that at 1 µg∙mL^−1^ of Ce6, C@HPOC showed the highest PDT effect (80% apoptosis ratio of 4T1 cells) compared to Ce6 and C@HSA under the same conditions, indicating that C@HPOC boosted the PDT effect to kill tumour cells. In addition, according to the in vivo results, O_2_-boosted PDT of C@HPOC provoked immunogenic cell death with enhanced release of danger-associated molecular patterns from 4T1 tumour cells and then promoted the maturation of dendritic cells. Finally, the well-defined C@HPOC evoked O_2_-enhanced immunogenic PDT, which not only destroyed the primary tumours but also effectively suppressed distant tumours and lung metastasis in metastatic triple-negative breast cancer model by evoking systemic anti-tumour immunity. 

**Figure 3:**
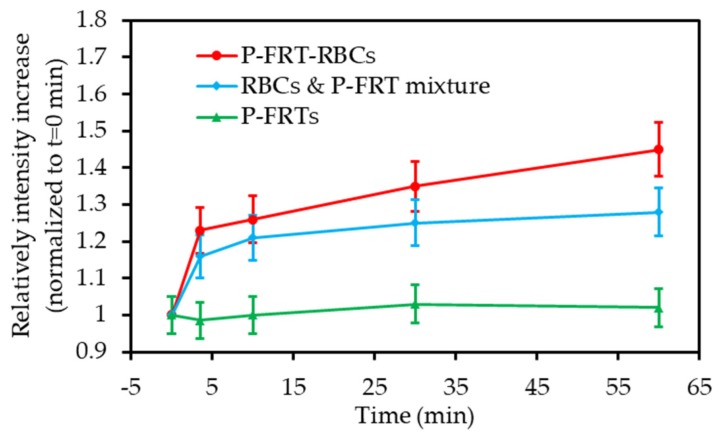
P-FRT-RBCs showed enhanced PDT effect under hypoxic environments. Comparison of ^1^O_2_ generation among P-FRT-RBCs, a mixture of RBCs and free P-FRTs, and free P-FRTs, conducted in an Ar-filled cuvette. The cuvette was irradiated by a 671 nm laser (0.1 W∙cm^−2^) for up to 60 min. SOSG was used as an indicator of ^1^O_2_ production. Adapted from Tang et al. [38].

Tang et al. [38] developed a novel red blood cells (RBC)-facilitated PDT methodology. They first loaded the phthalocyanine ZnF_16_-Pc into ferritin NPs and then coupled the ZnF_16_-Pc-loaded ferritins (P-FRT) onto RBC membranes to afford P-FRT-RBC-NPs. According to the in vitro and hypoxic tumour models, using RBCs as ZnF_16_-Pc carriers could enhance the PDT efficiency. It was shown that RBCs could provide O_2_ to enable sustained ^1^O_2_ production even when P-FRT-RBC NPs were under hypoxic conditions (Figure 3).

P-FRT-RBCs were injected onto U87MG human glioma tumour bearing mice (671 nm, 100 mW∙cm^−2^, 30 min). Significant improvement in the PDT efficiency was observed with P-FRT-RBC or O_2_-treated P-FRT-RBC groups compared to that of the P-RBC and CO-treated P-FRT-RBC groups (76.7% of tumour suppression). Such results validated the contribution of O_2_ released from RBCs in the enhanced treatment.

**Figure 4:**
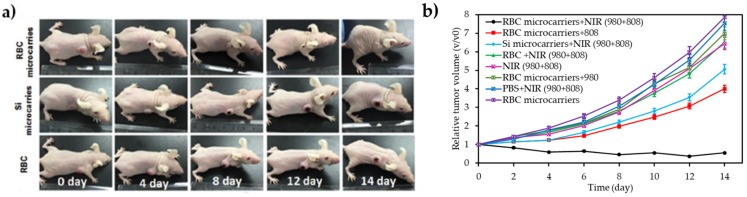
PDT for hypoxia tumours. (**a**) Digital photos of U87MG tumour-bearing mice after 14 days of O_2_ release and PDT treatments under NIR irradiation. From up to down mice were treated with RBC microcarriers + 980-nm +808-nm laser; Si microcarriers + 980-nm +808-nm laser; RBC + 980-nm +808-nm laser. (**b**) Tumour growth profiles of the mice bearing U87MG tumour with different treatments. Reprinted from [39] with permission from Elsevier, Ltd, Copyright 2017.

Wang et al. [39] reported a novel strategy for overcoming biological barriers and site specific hypoxia cancer therapy under NIR control. The latter consisted of preparing orthogonal excitation-emission UCNPs functionalized with a novel ultrasensitive specific hypoxia probe (HP) and RB, conjugated to the surface of RBC to finally obtain RBC microcarriers. According to the in vitro PDT results under hypoxic conditions, the inactive HP present in RBC microcarriers could be transformed into an active state specifically to trigger the O_2_ release from oxygenated Hb under 980 nm excitation. PDT efficiency enhanced greatly under 808 nm excitation because of the increasing of O_2_ amount from RBC microcarriers. Consequently, the highest cell mortality (60%) was achieved with RBC microcarriers after alternately irradiating by 980 nm and 808 nm laser, indicating a highest PDT efficacy which was due to the large amount of released O_2_. PDT for hypoxia tumours studies was investigated onto U87MG solid tumour-bearing mice. Much higher anti-tumour efficacy by remarkably regressing the solid tumour volumes was observed with RBC microcarriers in the presence of the alternate 980 nm and 808 nm laser irradiation, compared to that with Si microcarriers and RBC alone (Figure 4a,b). 

**Figure 5:**
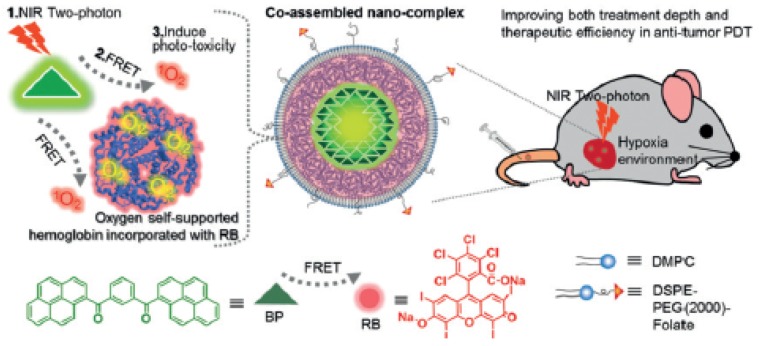
The BP@RB-Hb structure and the process of one-/two photon. Reprinted from [40] with permission from John Wiley and Sons, Copyright 2018.

Cao et al. [40] designed a multi-functional nanocomplexe (BP@RB-Hb) by simple molecular assembly of bis(pyrene) (BP), RB, Hb and nanoliposomes (Figure 5) to improve both the depth and the effectiveness of antitumour PDT treatment. In brief, upon two-photon laser irradiation, RB was excited indirectly through intra-particle FRET mechanism for improving treatment depth. At meantime, Hb could supply extra O_2_ into tumour through targeting effect for enhanced PDT efficiency. 

In vitro studies were performed on MCF-7 cells lines at different concentrations of deoxy-BP@RB-Hb or BP@RB-Hb (0–1.25 mg∙mL^−1^). Upon mono-photon laser irradiation (150 W, 480 nm cutoff filter), BP@RB-Hb showed the best photocytotoxicity, which was explained by more ROS production by BP@RB-Hb via intra-particle FRET between RB and BP species. 

**Figure 6:**
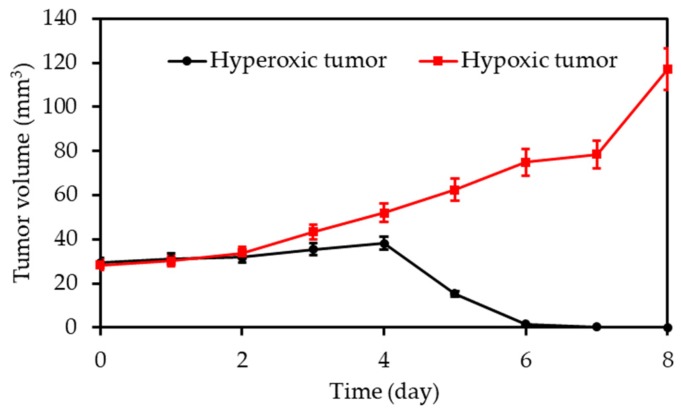
Tumour volume under hyperoxia and hypoxia. Adapted from Cao et al. [40]

Finally, in vivo photodynamic studies were also done in a mouse cancer model (BALB/c nude mice). BP@RB-Hb or BP@RB was injected to the mouse followed by two-photon laser (800 nm, 390 mW∙cm^−2^, 8 min). A significant anti-tumour effect was observed in the case of group injected with BP@RB-Hb. Furthermore, in vivo BP@RB-Hb showed a higher anti-tumour efficiency to the hyperoxic tumour than the hypoxic tumour after two-photon laser irradiation (Figure 6). 

Guo et al. [41] designed an O_2_-delivering photosensitive liposome (LIH) by loading complexes of Hb and ICG. LIH showed out-standing ability as an O_2_ carrier and could generate massive ^1^O_2_ under NIR laser irradiation (808 nm, 1 W∙cm^−2^, 1 min), even in hypoxia conditions. In vitro O_2_ carrying was investigated on CT-26 cancer colon cells. It was found LIH could not only get inside CT-26 cells and deliver O_2_ into cells in hypoxia condition, but also down-regulated hypoxia-associated protein to cells. Thus, the sufficient ^1^O_2_ produced by LIH under NIR laser treatment (808 nm, 1 W∙cm^−2^, 1 min) enhanced dramatically the PDT phototoxicity in hypoxic CT-26 cells. According to the in vivo studies on mice bearing tumours, LIH accumulated preferentially into subcutaneous and orthotopic CT-26 tumours. In addition, the vascular endothelial growth factor (VEGF) and Hypoxia-inducible factor-1α (HIF-1α) expression level in LIH treated tumours was also down-regulated and this state was maintained for several days. Consequently, under NIR laser irradiation (808 nm, 1.5 W∙cm^−2^, 3 min) dramatically enhanced PDT efficacy against hypoxic tumour in vivo was observed. 

Liu and his team [42] synthesized Hb-conjugated biodegradable polypeptides as O_2_ carrier via click reaction between polypeptides and Hb-BODIPY-Br_2_ NPs denoted (p-Hb-B-NPs). The O_2_-carrying ability of the prepared NPs was confirmed by comparing the dissolved O_2_ concentration of p-Hb-B-NPs to that of O_2_ saturated ultrapure water. PDT efficiency of the NPs was investigated on hepatocellular carcinoma HepG2 cancer cells (620 nm, 25 mW∙cm^−2^, 10 min) under normal or N_2_ atmosphere and compared to that of p-B-NPs. Even under low light irradiation both NPs showed phototoxic effect under normal atmosphere, where the best effect was obtained at a 4 µM p-Hb-B-NPs concentration, however a non-phototoxic effect was observed under N_2_ atmosphere. Furthermore, p-Hb-B-NPs found to be able to release O_2_ even in hypoxic conditions. 

Xu et al. [43] prepared a new biocompatible and stable nanocarrier (HbTcMs) with O_2_ supply for enhanced PDT. A well-defined triblock copolymer mPEG-b-PAA-b-PS copolymer could self- assemble in aqueous solution to form micelles. The micelles and the PS (carboxyphenyl-porphyrin, TCPP) both covalently conjugated to Hb via amidation reactions formed a nanocarrier (HbTcMs). According to the O_2_ binding ability results, HbTcMs was found to be able not only to retain the O_2_ binding capacity as Hb did, but also showed better stability against both oxidation and trypsin digestion. HbTcMs conjugate showed little dark toxicity and rapid efficiency of cellular uptake by 4T1 cells. More importantly, under light irradiation (600 nm, 70 mW∙cm^−2^, 2 min), HbTcMs could generate more ^1^O_2_ to exert better phototoxicity in vitro compared to the carriers without O_2_ supply. 

Liu et al. [44] designed man-made red blood cells (RBCs). A complex between Hb and polydopamine (PDA, an enzyme-mimicking) was constructed and methylene blue (MB) was encapsulated inside the biovesicle (Hb-PDA-MB, 500 nm in transverse length and 2000 nm in longitudinal length). To evaluate the influence of Hb other complexes were synthesized in which Hb was substituted with albumin bovine serum (BSA) (called BSA-PDA@RBCM and BSA-PDA-MB@RBCM, respectively). Hb was unchanged in the vesicle and the capability of Hb to carry O_2_ was the same for free Hb and Hb into the vesicle. ^1^O_2_ formation was higher for MB inside the vesicle than in solution due to the extra O_2_ supply. In 4T1 cell line upon light illumination (660 nm, 30 mW∙cm^−2^, 1 min) DCFH-DA revealed the formation of ^1^O_2_. In hypoxic media, the vesicles were still able to produce ^1^O_2_, whereas almost no more ^1^O_2_ was produced by MB alone. The phototoxicity of Hb-PDA-MB decreased by 10% in hypoxic media compared to normoxic one whereas a decrease of 50% was obtained with free MB (660 nm, 220 mW∙cm^−2^, 5 min). In vivo in 4T1 tumour bearing mice, they used the same vesicle labelling with both Ce6 and DiR (1,1-dioctadecyl-3,3,3,3-tetramethylindotricarbocyanine) and could observe that the tumour stopped growing. Some tumours even completely disappeared (Table 3).

**Figure 7:**
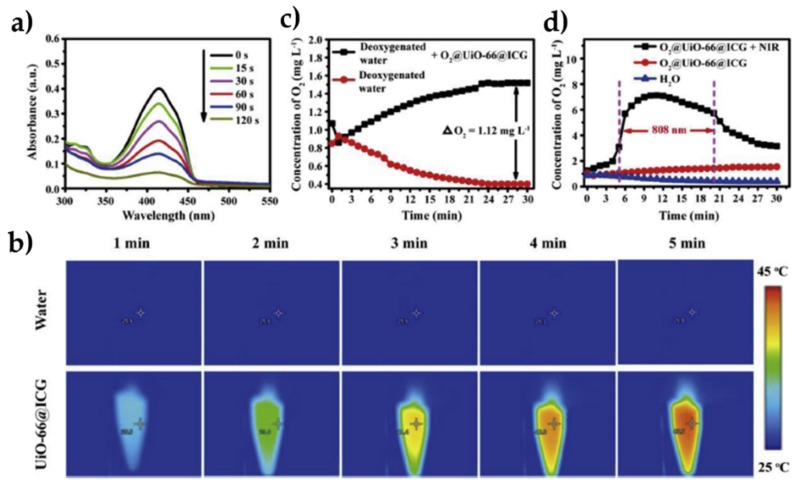
(**a**) The absorbance changes of DPBF treated with UiO-66@ICG in DMF after 808 nm laser irradiation for different times. (**b**) Infrared thermal images of pure H_2_O and UiO-66@ICG aqueous dispersion (1 mg∙mL^−1^) after irradiation for 5 min by 808 nm laser (0.06 W∙cm^−2^). (**c**) O_2_ concentration in 20 mL deoxygenated water before and after adding 5 mg of O_2_@UiO-66@ICG. (**d**) O_2_ concentration changes in solutions of O_2_@UiO-66@ICG under irradiation by 808 nm laser. DO_2_: Enhanced O_2_ concentration. The O_2_ loading capacity per 1 g of ICG@UiO-66 was calculated as follows: the loading capacity of ICG@UiO-66 ¼ (DO_2_: Enhanced O_2_ concentration)/ (ICG@UiO-66 concentration). The O_2_ loading capacity of ICG@UiO-66 was determined to be ~140 mmol∙g^−1^ or 4.48 mg∙g^−1^. Reprinted from [45] with permission from Elsevier Ltd, Copyright 2018.

Zirconium (IV)-based MOF (UiO-66) NPs (diameter 55 nm) coated with ICG were loaded with O_2_ and coextruded with Hb-RBC membranes by Gao et al. [45]. They presented high O_2_ storage capacities. The production of ^1^O_2_ was observed by DPBF after irradiation at 808 nm during 2 min (Figure 7). A photothermal effect was measured with a temperature of 43 °C after 5 min irradiation. The NPs were encapsulated in RBC membrane from male ICR mice and the circulation half-lives increased. The uptake of the NP in macrophages (RAW264.7 cell line) was confirmed. The ^1^O_2_ production in multicellular tumour spheroids (MCTSs) was observed by DCFH-DA. The PDT efficiency was assessed showing a high cell mortality after NIR irradiation (808 nm, 0.06 W∙cm^−2^, 5 min). Intravenous injection of the NP in MCF-7 and light irradiation (808 nm, 0.06 W∙cm^−2^, 1 min) tumour-bearing mice showed a notably tumour growth inhibition.

### 2.3. Perfluorocarbon

Perfluorocarbons (PFCs) are organofluorine compounds with the formula CxFy. PFCs are capable of dissolving relatively high volumes of gases due to the weak intermolecular interactions in these PFCs. They can maintain a higher O_2_ content than the tumour matrix at a given O_2_ partial pressure thanks to their high O_2_ capacity. In this context, since 2016 19 publications have described the use of PFC to relieve tumour hypoxia and improve PDT efficiency. It can be used as the contrast enhancement agent of ultrasonography in the clinic [46], different formulations of liquid PFC have been explored to serve as an artificial blood substitute. PFCs could be excreted by exhalation from lungs or skin pores. Oxypherol (Fluosol-43) has obtained FDA approval in improving myocardial oxygenation and preventing abnormalities in ventricular function [47]. Oxygent™ is a concentrated PFC emulsion being developed for use as a temporary O_2_ carrier. It has been approved in Russia to increase O_2_ delivery during acute blood loss.

Cheng et al. [48] developed nanodroplets of perfluorohexane (PFH) coated with a lipid shell incorporating IR780 as a PS (diameter 200 nm). The irradiation of NPs in water with an 808 nm laser for 5 s intervals during 60 s (2 W∙cm^−2^) showed an higher ^1^O_2_ production than irradiation of PFH or IR780 alone. MCF-7 cells or CT26 murine colon adenocarcinoma cells irradiated with 808 nm laser (20 s, 2 W∙cm^−2^) led to an increase of ^1^O_2_ production with the NPs compared to PFH or IR780 alone. Phototoxicity of the nanodroplets on the two cell lines was observed. Photothermal effect did not play any role. The effects of the NPs in hypoxia were studied in CT26 cells in hypoxic conditions. After irradiation, NPs still maintained superior cytotoxicity to traditional PDT. In vivo evaluation of ^1^O_2_ production on CT26 tumour-bearing mice with SOSG indicated higher ^1^O_2_ generation with the conjugate than with PFH or IR780 alone. The tumour growth of the mice was inhibited after intratumoural injection. The tumour volume of mice receiving NPs and irradiation (808 nm, 10+10 s, 2 W∙cm^−2^) was four folds lower than the volume of mice receiving IR780 alone.

PCF nanoemulsions (diameter 175 nm) containing a fluorous porphyrin were synthetized by Day et al. [49]. They could encapsulate O_2_ with high concentration and produced ^1^O_2_ after light illumination (420 nm, 8.5 mW∙cm^−2^, 30 min). A minimal leaching of fluorous porphyrin to the hydrophilic medium was detected. The uptake of the emulsions in human malignant melanoma cells (A375) was successfully observed with confocal microscopy. Cell death was observed after irradiation (420 nm, 8.5 mW∙cm^−2^, 30 min), whereas minimal death occurred with an emulsion containing a fluorous rhodamine. In summary, fluorous porphyrins sequestered in PFC nanoemulsions were able to deliver ^1^O and to perform efficient PDT.

**Figure 8:**
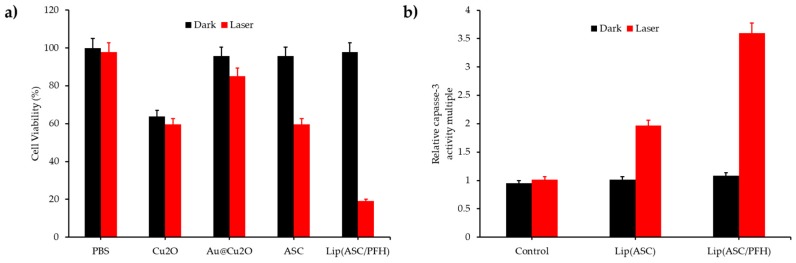
PDT in vitro on MCF-7 cells. (**a**) Cytotoxicity of different samples in the presence or absence of irradiation (670 nm, 0.48 W∙cm^−2^). (**b**) Relative activity multiple of caspase-3 protein in MCF-7 cells activated by Lip(ASC) and Lip(ASC/PFH) (20 μg∙mL^−1^) with or without 670 nm irradiation for 10 min. Adapted from Liu et al. [50].

Liu, et al. [50] synthesized a core shell NP (Au core, SiO_2_ and CuO_2_ shell) incorporated into PHF droplets to form a liposome (diameter 190 nm). The plasmon-induced resonance energy transfer (PIRET) from Au to Cu_2_O, Au@SiO_2_@Cu_2_O (670 nm, 0.48 mW∙cm^−2^, 10 min) produced ^1^O_2_ with a quantum yield evaluated at 0.71. NPs successfully incorporated into MCF-7 cells. ^1^O_2_ production in these cells was greatly enhanced by the O_2_ supply of PFH. A strong phototoxicity appeared after light illumination (670 nm, 0.48 mW∙cm^−2^, 10 min) (Figure 8a). The caspase 3 protein activity, an apoptosis indicator, showed a 3.6 higher amount than without irradiation (Figure 8b). The NPs induced significant phototoxicity to MCF-7 cells under both normoxic and hypoxic conditions. The O_2_ self-enriched effect of the NPs could guarantee PDT efficacy in MCF-7 cells. Irradiation of multicellular tumour spheroids (MCTS) of MCF-7 cells showed a high mortality. Tumour growth measurements on MCF-7 cancer-bearing female Balb/c mice conducted to a complete inhibition during 14 days after injection of the NP and irradiation (670 nm, 0.48 mW∙cm-^2^, 10 min).

Que et al. [51] elaborated fluorine-containing NPs (a core of pentafluorophenyl and 5,10,15,20-tetrakis(4-aminophenyl) porphyrin) coated with PEG as spherical micelles (diameter 30 nm). ^1^O_2_ production, measured with ABDA was highly dependent of the concentration of PFC in the core, as O_2_ supplier (655 nm, 1.52 mW∙cm^−2^, 15 min). The porphyrin in copolymers was then loaded with Pd. The heavy atom effect played a role in the enhanced PDT efficacy. No dark cytotoxicity was observed on SMMC-7721 cells (human hepatoma cell line). The phototoxicity of the micelles after irradiation (655 nm, 1.52 mW∙cm^−2^, 60 min) showed a cell viability less than 30% and correlated with the PFC amount.

A nanoliposome was prepared by Sheng et al. [52] by incorporating perfluorooctyl bromide (PFOB) and ICG into a core coated by a bilipidic shell (diameter 200 nm). The release of O_2_ from the NPs was assured by diffusion during at least 24 h. The photothermal effect of the NP was evaluated after laser irradiation (808 nm, 1 W∙cm^−2^, 5 min) with a temperature increase from 43 to 50 °C. ^1^O_2_ measured by SOSG under hypoxia was highly increased with the conjugate compared to ICG or liposome-ICG alone. The ROS production measured in vitro on human triple-negative breast cancer MDA-MB-231 cells led to the same results. Under irradiation (808 nm, 1 W∙cm^−2^, 3 min) the cell viability decreased to 10% both in normoxia and hypoxia by a combination of PDT and PTT effects. Mice bearing MDA-MB-231 tumours treated with the conjugate and irradiated (808 nm, 1 W∙cm^−2^, 10 min) achieved almost complete tumour destruction without tumour recurrence. 

**Figure 9:**
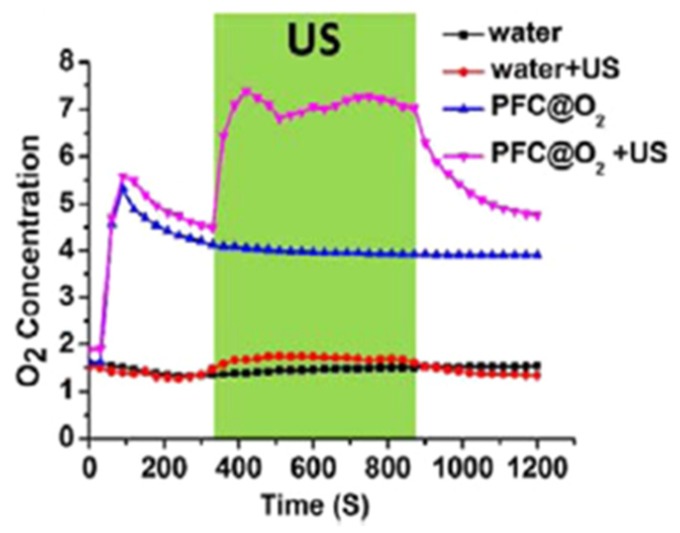
Time-dependent changes of dissolved O_2_ concentrations in deoxygenated pure water without or with addition of O_2_-loaded PFC nanoemulsion (PFC@O_2_). An US treatment was applied on these solutions within the indicated period. Reprinted from [53] with permission from the American Chemical Society, Copyright 2016.

Song et al. [53] synthesized nanodroplets of PFC (diameter 160 nm) loaded with O_2_ and stabilized by albumin. Ce6 was modified (three hexylamine were added to increase the hydrophobicity) and loaded into the lipid bilayers of PEG-modified liposomes. The release of O_2_ was triggered by an external ultrasound activation (Figure 9). This activation enhanced PDT and radiotherapy efficiency. 4T1 tumour-bearing mice placed under hyperoxic inhalation were treated with ultrasound (US). Oxygenation levels showed an increase of 50% for the mice treated with the conjugate. 4T1 tumour-bearing mice were treated with US and irradiated (671 nm, 1.2 W∙cm^−2^, 5 min). Mice tumour growth was significantly inhibited compared to the control group. Similar results were obtained on mice bearing CT-26 colon cancer tumours.

**Figure 10:**
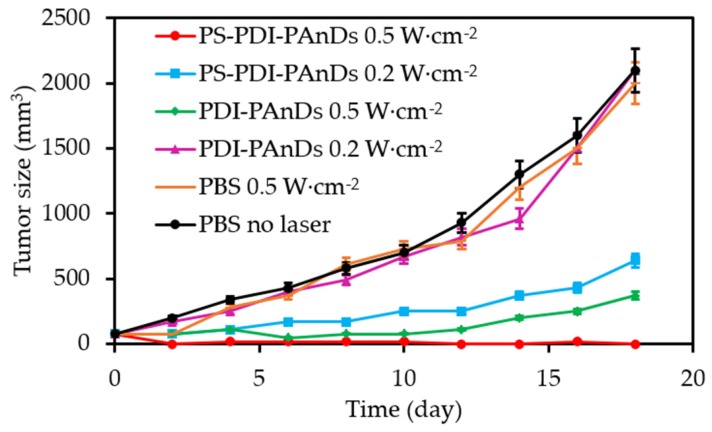
Tumour growth curves. Complete tumour eradication was found in the combinational PTT/Oxy-PDT treatment group with i.v. injection of PS-PDI-PAnDs plus a 0.5 W∙cm^−2^ laser irradiation. Significant tumour growth inhibition was also observed with animals treated with PTT only (i.v. injection of PS-PDI-PAnPs plus a 0.5 W∙cm^−2^ laser irradiation) and Oxy-PDT only (i.v. injection of PS-PDI-PAnDs plus a 0.2 W∙cm^−2^ laser irradiation), indicating tumour growth inhibition rates of 82.3% and 67.5%, respectively, on day 18. Adapted from Tang et al. [54].

Tang et al. [54] designed PFC nanodroplets coated with IR Dye 800CW (for in vivo imaging) and incorporating ZnF_16_Pc as a PS and perylene diimide (PDI) as photoacoustic agent (PA) imaging agent (diameter 110 nm). The high O_2_ solubility in PFC enhanced PDT. The PDI agent converted light energy into heat for contrast-enhanced ultrasound imaging and for a photothermal effect. The PA intensity was registered proportionally to the PDI concentration. Under irradiation at 671 nm at different irradiance (until 1.2 W∙cm^−2^, 5 min) the temperature of the solutions increased until 89 °C showing the photothermal effect for an irradiance of 1.2 W∙cm^−2^. Phototoxicity was observed after light irradiation (671 nm, 100 mW∙cm^−2^, 200 s) on U87MG cells with a viability less than 34% without photothermal effect. ^1^O_2_ was produced in normoxic and hypoxic media due to O_2_ supply by PFC. Same conclusions were observed on U87MG cells. Mice irradiated at 500 mW∙cm^−2^ during 10 min (671 nm) presented a complete tumour eradication (Figure 10). 

Tang et al. [55] designed a nanodroplet of perfluorohexane (PFH) as core and a lipid shell containing IR780 as the PS. The high concentration of O_2_ on PFH is playing as a sponge for O_2_ and was able to overcome hypoxia in tumours. After irradiation (808 nm, 2 W∙cm^−2^, 20 s) ^1^O_2_ production measured by the SOSG probe was higher with the conjugate than the lipidic NPs without PFH even when the conjugate was in hypoxic conditions. CT26 murine colon adenocarcinoma cells incubated and irradiated (808 nm, 2 W∙cm^−2^, 20 s) showed a higher level of ^1^O_2_ with the conjugates than with the lipidic NPs alone. The phototoxicity of the conjugate in hypoxia presented a high mortality in the CT26 cells, while almost no cells were killed with the lipidic NPs. The NPs were injected intravenously in mice bearing CT26 subcutaneous tumours kept in hypoxia (7% O_2_) during 30 min. After light illumination (808 nm, 2 W∙cm^−2^, 10+10 s) the tumour growth was inhibited whereas no efficacy was observed for the lipidic NP. 

Tao et al. [56] prepared fluorinated covalent organic polymers (COPs) by linking covalently a porphyrin (THPP) with perfluorosebacic acid (PFSEA) and PEG (diameter ~60 nm). PFCE, a type of PFC, was loaded into the porous structure of the NP. The dissolved O_2_ concentration was enhanced compared to the control. ^1^O_2_ level measured by SOSG showed a significant increased production after irradiation (660 nm, 10 mW∙cm^−2^, 30 min). 

**Figure 11:**
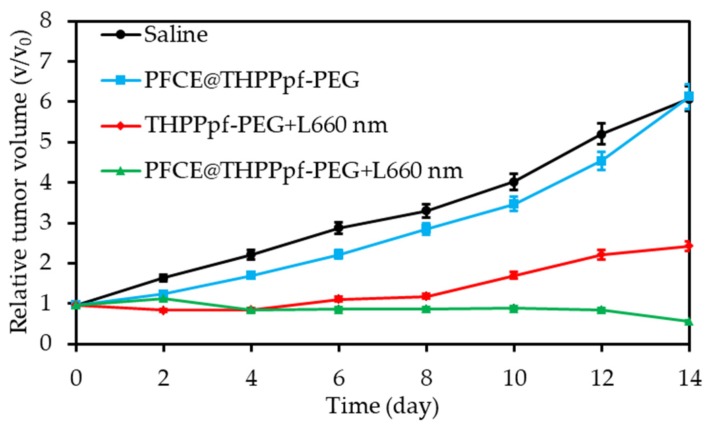
Tumour growth curves of four groups of mice after treatments as indicated. V_0_ and V stand for the tumour volumes before and after the treatments, respectively. Adapted from Tao et al. [56].

Cellular uptake of the conjugates was successfully achieved on 4T1 cells. Phototoxicity of 80% was evaluated after irradiation (660 nm, 10 mW∙cm^−2^, 60 min). The tumour growth of 4T1 tumour-bearing mice after intravenous injection and irradiation (660 nm, 50 mW∙cm^−2^, 30 min) was fully inhibited after 14 days (Figure 11).

Wang et al. [57] synthesized hollow MoSx NPs (HMoSx) by incorporating PFH in the core and loaded with albumin (HSA) and aluminum phthalocyanine (AlPc) (diameter 140 nm). The NP were then loaded with O_2_. A photothermal effect was observed after irradiation (670 nm, 1 W∙cm^−2^, 5 min), the temperature reached 68 °C. ^1^O_2_ production measured by DPBF showed a significant enhancement for the conjugate after irradiation. Phototoxicity assays realized on 4T1 cell lines led to an efficient activity of the conjugate on normoxia and hypoxia, when it was loaded with O_2_. Intracellular ROS generation on 4T1 cells was also confirmed by measurement with the ROS-ID probe. Tumour growth measurements on 4T1 tumour mice conducted to a complete inhibition during 16 days after injection of the conjugate and irradiation (670 nm, 1 W∙cm^−2^, 5 min) confirming the dual PTT/PDT effect.

**Figure 12:**
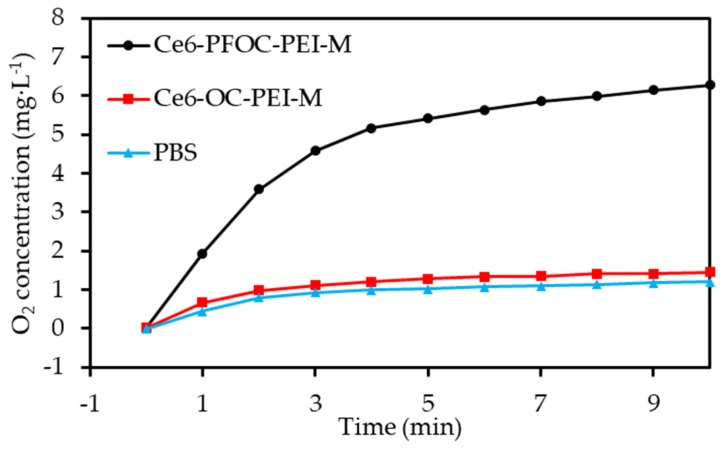
O_2_ concentration changed after the addition of O_2_ saturated Ce6-PFOC-PEI-M, Ce6-OC-PEI-M and PBS into deoxygenated water. Adapted from Wang et al. [58].

Wang et al. [58] prepared a polymeric micelle constituted of perfluorooctanoic acid and branched polyethyleneimine and incorporating Ce6 (diameter 120 nm). A non-fluorinated micelle was prepared as control. The two NPs were loaded with O_2_ and the O_2_ released concentration was significantly higher for the fluorinated NP (Figure 12). ROS production measured by DCFH-DA on C6 cells showed high concentration for the conjugate after irradiation (630 nm, 30 mW∙cm^−2^, 3 min). Hypoxia was greatly overcome by the conjugate in C6 cells compared to the control. Tumour growth measurements on BALB/c nude mice bearing C6 tumours conducted to a significant inhibition during 12 days after intravenous injection of the NP and irradiation (660 nm, 500 mW∙cm^−2^, 20 min). 

Yu et al. [59] designed PFH nanoliposomes incorporating O_2_ and Ce6 (diameter 100 nm). PFH was able to encapsulate and then release O_2_. The dissolved O_2_ concentration was greatly enhanced during ultrasonic activation. The ROS generation using DPBF showed a time-dependent production for the conjugate in contrary to the control without PFH. Phototoxicity of the conjugate was clearly established in contrary to the control without PFH. In vivo tumour growth volume measurements on mice bearing subcutaneous 4T1 tumour showed that the conjugate induced the best tumour inhibition (670 nm, 1 W∙cm^−2^, 1 min).

Yuan et al. [60] synthesized fluorinated polypeptide micelles incorporating a NIR PS BODIPY (diameter 150 nm). The NPs was water dispersible and the PFC concentration allowed a high O_2_ loading. Higher levels of dissolved O_2_ were measured with the conjugate compared to the non fluorined one. The same tendency was observed for the photosensitized ^1^O_2_ generation after irradiation at 635 nm (0.5 mW∙cm^−2^, 360 s). Phototoxicity (635 nm, 34 mW∙cm^−2^, 10 min) measured on HepG2 cells at the same irradiance showed a better efficacy of the conjugate compared to the non fluorined one, confirming the role of PFH in O_2_ supply. Tumour growth measurements on 4T1 tumour-bearing mice conducted to a complete inhibition during 19 days after injection of the NP and irradiation (635 nm, 100 mW∙cm^−2^, 10 min). 

**Figure 13:**
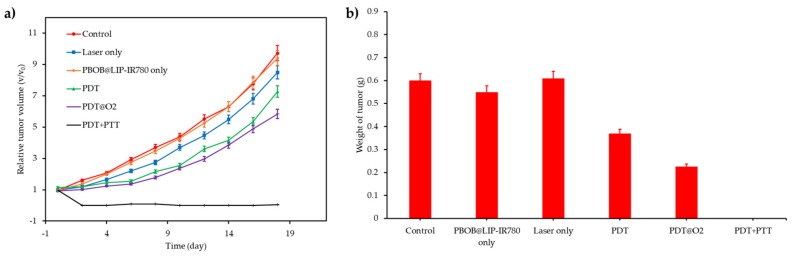
(**a**) Tumour growth curves of six groups after various treatments. (**b**) Weight of tumours 18 d post various treatments. (Values are means±s.d., n = 5, * *p* < 0.05.). Adapted from Zhang et al. [61].

Zhang et al. [61] synthesized perfluorooctyl bromide nanoliposomes incorporation IR780 NIR PS (diameter 270 nm). The photothermal effect of the NP was evaluated after laser irradiation (808 nm, 1 W∙cm^−2^, 5 min) with a temperature increase from 25 °C to 70 °C. ^1^O_2_ generation was measured by SOSG under the same fluence with a high amount for the conjugate in contrary to the non-fluorinated NP. Intracellular ^1^O_2_ generation was observed after light irradiation (808 nm, 1 W∙cm^−2^, 5 min). PTT and PDT treatments on 4T1 cancer cells showed less viability for the fluorinated conjugate and better efficacy with combined PTT/PDT. Tumour growth measurements on 4T1 cancer-bearing mice conducted to a complete inhibition during 18 days after injection of the NP and PTT/PDT treatment (808 nm laser (on 30 s, off to room temperature, 20 cycles), 1.0 W∙cm^−2^) (Figure 13a,b).

Zhao et al. [62] elaborated a NP made of a core of perfluorooctyl bromide and a shell with the PS IR780 and a tumour penetrating peptide CRGDK (diameter 210 nm). Hypoxia measurements made on MDA-MB-231 tumour-bearing mice showed a significant attenuation in the tumour where the conjugate was injected, confirming the role of PFH n in the O_2_ delivery. Tumour growth measurements on MDA-MB-231 tumour-bearing mice conducted to a complete inhibition during 13 days after injection of the NP and irradiation (808 nm, 2 W∙cm^−2^, 20 s). 

**Figure 14:**
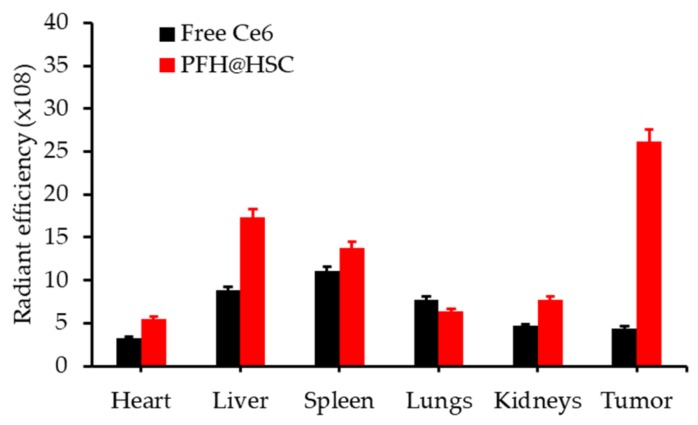
In vivo biodistribution of NPs. Semiquantitative fluorescence analysis of the radiant efficiency of organs and tumours isolated at 24 h post-injection. Adapted from Hu et al. [63]

NPs with a PFH core and a hyaluronic acid (HA) and Ce6 shell were synthetized (diameter 220 nm) by Hu D. et al. [63]. These “on-off” NPs were redox-activatable and could release Ce6 and O_2_ in hypoxic tumours. The ^1^O_2_ of the conjugate increased significantly in reducing conditions after light irradiation (660 nm, 100 mW∙cm^−2^, 5 min). The cellular uptake of the NP was studied on MDA-MB-231 cells. The ROS production in vitro was significantly higher with that conjugate than with NP with incomplete composition. NPs were intravenously injected in MDA-MB-231 breast tumour xenograft mouse model. NPs retention in the organs was stronger after 24 h than for free Ce6, especially in the tumour (5.8 fold) (Figure 14). 

Table 4 lists the recents publications with PFH.

The in vivo hypoxia intensity declined with the NP compared to the NP without PFH. The NP showed effective tumour growth inhibition compared to NP with incomplete composition even if the tumour did not disappear completely (660 nm, 100 mW∙cm^−2^, 10 min).

Hu et al. [64] synthesized different amphiphilic fluorous random copolymers with different amount of perfluorocarbon, that embed Hypocrellin B (HB) as PS, assembled themself into micelles in water (diameter 200 nm). The O_2_ carrying ability of the conjugate was related to the PFC content. ^1^O_2_ production of HB after light irradiation (630 nm, 6 J/well) of the conjugate measured with SOSG was equivalent as for HB in water. The phototoxicity (630 nm, 20 mW∙cm^−2^, 300 s) of the conjugate in human lung carcinoma H1299 cells depended on the amount of PFC. The highest amount led to the best efficiency.

Ma et al. [65] prepared NPs with fluorocarbon chains and IR780 PS in the core and iRGD peptides on the shell (diameter 80 nm). ^1^O_2_ concentration was measured in vitro and in vivo with SOSG showing increased values for the conjugate (808 nm, 2 W∙cm^−2^, 20 s). ROS generation measured on 4T1 tumour spheroids showed an enhanced concentration even inside the spheroid showing efficacy of the tumour-penetrating iRGD peptide. High phototoxicity was observed at 7.5 µg∙mL^−1^ with a cell viability less than 20% (808 nm, 2 W∙cm^−2^, 20 s). O_2_ concentration in 4T1 solid tumours measured by photoacoustic system showed an enhanced amount after injection of the NPs and irradiation. Tumour growth measurements on 4T1 cancer-bearing mice conducted to a complete inhibition during 12 days after injection of the NPs and irradiation (808 nm, 2 W∙cm^−2^, 5 min) (Table 4).

### 2.4. O_2_ Microbubbles

Free O_2_ gas bubbles are not recommended to be directly injected into blood flow for hemolysis reason. A new approach of O_2_ delivery, well documented in a review of Khan et al. [66] consists in the use of O_2_ loaded micro-/nanobubbles to bring O_2_ to the hypoxic tumour without side effects. In such particles, the shell is generally a monolayer biomaterial (lipids, proteins, polymers…) that encapsulates the core gas.

Huang et al. [67] have developed a new strategy for externally triggered PDT of tumour hypoxia using bone marrow-derived monocytes/macrophages as cellular vehicles for co-transport of O_2_ and Ce6. Superparamagnetic iron oxide NPs (SPIONs) were co-entrapped within the polymer to afford functional bubbles denoted (SCOPBs). According to the in vitro studies, these functional bubbles were found harmless to cellular hosts without external trigger. However, significant apoptosis of cancer cells co-incubated with therapeutic monocytes was observed when treated with high frequency magnetic field (HFMF) and red light laser irradiation due to the combined effect of PDT and magnetic hyperthermia was observed. Furthermore, upon tumour treatment with HFHM (9 min) and red laser irradiation (660 nm, 100 mW∙cm^−2^, 10 min), the therapeutic monocytes exhibited a superior performance in inhibiting tumour growth on tramp-C1 tumour-bearing mice. Moreover, immunohistochemistry (IHC) staining of tumour sections confirmed the successful cellular transport of the therapeutic payloads to tumour hypoxia and the pronounced antitumour effect elicited by combined hyperthermia/PDT along with the additional O_2_ supply.

In 2018, Song et al. [68] developed nanocapsules composed of a decane core and a polymer shell linked to Ce6, surrounded by lipid molecules conjugated to a hydrophilic polymer PEG (263.2±10.3 nm with a thickness of about 20 nm for the shell). The O_2_ nanobubbles (NBs-O_2_) were finally obtained by O_2_ infusion in the nanocapsules. Fluorinated caps on the PCL polymer avoided the liberation of O_2_ at an undesired place. The authors had then compared their ability to release O_2_ in comparison with well-known O_2_ carrier, lipid-coated O_2_ microbubbles (LOMs). They followed the level of O_2_ dissolved in a hypoxic solution over time. LOMs showed a higher and faster increase of O_2_ concentration, at the beginning (first minute) than NBs-O_2_ but they decreased rapidly to a lower level than NBs-O_2_. This indicated a better robustness of these nanobubbles due to their thicker shell. They also compared the effect of O_2_ release on the ^1^O_2_ production of Ce6 and observed a level 3 times higher for NBs-O_2_ in comparison with Ce6 alone. In vitro studies on C6 glioma showed that they were strongly accumulated in cytoplasm after endocytosis in comparison with Ce6 alone. In vivo studies were performed on glioma-bearing nude mice. They evaluated their ability to improve the PDT treatment and showed a significant decrease of tumour growth for mice treated with these nanobubbles and laser irradiation (670 nm, 300 mW∙cm^−2^, 30 min) in comparison with the control group (Table 5).

### 2.5. Other 

Niu et al. [69] designed β-NaGdF_4_:Yb^3+^/Er^3+^ upconverting NPs (diameter 50 nm) associated with gold oxide (Au_2_O_3_), porous silica, Ce6, cyclo-RGD and PEG. Upon a NIR irradiation, Au_2_O_3_ is able to produce O_2_ assisted by UCNPs via (FRET). When FRET occurred between UCNPs and Au/Au_2_O_3_ under NIR light, Au_2_O_3_ underwent a decomposition into Au and O_2_. The O_2_ production under NIR irradiation (980 nm, 30 min, 0.5 mW∙cm^−2^) was demonstrated using a Ru probe. FRET was observed between the NP and Au_2_O_3_ for O_2_ production and between the NPs and Ce6 for a PDT effect. The phototoxicity of the NPs on human cervical cancer cells (HeLa) was demonstrated. The NPs were intravenous injected in nude mice inoculated with HeLa tumour cells. After NIR irradiation (980 nm, 30 min, 0.5 mW∙cm^−2^), the relative tumour volume of the treated group stayed constant whereas it increased in the control group. The Ns presented also a photothermal effect that could be used for PA.

## 3. Modification of Tumour Microenvironment (TME) 

The "normal" microenvironment is complex and dynamic. Its cellular component encompasses all neighbouring cells (adjacent cells belonging to the same tissue) and cells residing in their direct environment (fibroblasts, vascular cells, dendritic cells, immune system cells, etc.). The TME or stroma is defined as the cellular components, molecular components and mechanical stresses surrounding and interacting with tumour cells. It differs from the "normal" microenvironment by the biochemical composition of the extra cellular matrix and especially by the fact that the stromal cell populations, although not transformed, are subverted and controlled by the tumour cells to meet their own needs. It has been widely demonstrated that TME plays a major role in bone marrow clearance and tumour invasion, as well as resistance to chemotherapy [70]. By modifying the TME, it seems possible through specific response pathways, such as that of the proteins of the Hypoxia Inducible Factor (HIF) family, to induce the activation of several signalling pathways that regulate many cellular processes such as angiogenesis, metabolism, cell proliferation and apoptosis.

### 3.1. H_2_O_2_ Decomposition

It is known that cancer cells have higher levels of H_2_O_2_ than normal cells [71] because of the aberrant metabolism of cancer cell. The concentrations range from 10^−4^ to 10^−3^ M. One of the strategies to improve O_2_ levels in hypoxic areas is to design compounds that could degrade endogenous H_2_O_2_ to produce O_2_ inside the tumour. It is interesting to note that catalase is able to convert around 5 million H_2_O_2_ molecules per minute and catalase is explored nowadays to overcome tumour relief [72]. Moreover, another advantage of this strategy is that the decrease of H_2_O_2_ in the cells leads to a decrease of cancer cell proliferation [72,73,74]. 

#### 3.1.1. MnO_2_

One advantage of MnO_2_ is that it can adsorb different kinds of small molecules via physisorption or Mn-N coordination links. MnO_2_ produces O_2_ by catalytic H_2_O_2_ decomposition under neutral conditions, but also under acidic conditions by reaction with H^+^. This strategy is very new. The first publication has been written in 2015, and then we can find three publications in 2016, seven in 2017, 14 in 2018 and three already in 2019 (before March). Moreover, the efficacy of PDT is not only limited by tumour hypoxia but also over-expressed glutathione (GSH) in cancer cells that consumes ROS.

Xu et al. [75] synthesized a photoactive Mn(II) complex of BODIPY derivatives ([(BDA)MnCl_2_], Mn_1_) as a O_2_ generator under irradiation (green LED, 500–600 nm, 10 W) in water. Mn_1_ was loaded on graphene oxide (Mn_1_@GO) and an in vitro biological evaluation for Mn_1_ and Mn_1_@GO on HepG-2 cells in normal and hypoxic conditions was performed. In normal conditions, both Mn_1_ and Mn_1_@GO after light illumination (green LED, 500–600 nm, 15 min) were active against HepG-2 cells. It was also found that both compounds could be used as photoactivated anticancer materials since Mn1 and Mn_1_@GO were able to exhibit inhibition on the proliferation of HepG-2 cells when light irradiation. In particular, Mn_1_@GO reacted with H_2_O_2_ resulting in the formation of active species, and worked as H_2_O_2_ and light-activated anticancer NPs. Moreover, Mn_1_@GO could be an efficient anticancer material in hypoxic environments and Mn_I_ could target mitochondria and had some effects on the expression of HIF-1α in HepG-2 cells. 

Multifunctional MnO_2_ NPs loaded with Ce6 and coated with PEG were designed (Ce6 @ MnO_2_-PEG) by Zhu et al. [76]. In vitro studies in an O_2_-deficient atmosphere have shown that Ce6 @ MnO_2_-PEG NPs can effectively increase the efficiency of light-induced PDT (661 nm, 5 mW∙cm^−2^, 30 min) due to the increased intracellular level of O_2_. In vitro in 4T1 cells, it was found that Ce6@MnO_2_-PEG NPs were very effective, even under conditions of O_2_ deficiency. With systemic administration to mice bearing subcutaneous 4T1 tumours, Ce6@MnO_2_-PEG NPs showed effective accumulation inside the tumour, in which the MnO_2_ NPs gradually decomposed into Mn^2+^ ions. Meanwhile, as expected, the level of oxygenation of the tumour was significantly increased. Due to enhanced Ce6 uptake in the tumour and reversed tumour hypoxia, highly effective PDT treatment (661 nm, 5 mW∙cm^−2^, 1 h) of cancer was implemented using Ce6@MnO_2_-PEG NPs. Given the gradual decomposition of MnO_2_ NPs, easy filtration of Mn^2+^ in the kidneys, and the lack of obvious short-term in vivo toxicity observed in this study, MnO_2_ nanostructures could be a unique type of safe nanoplatform promising for cancer. 

**Figure 15:**
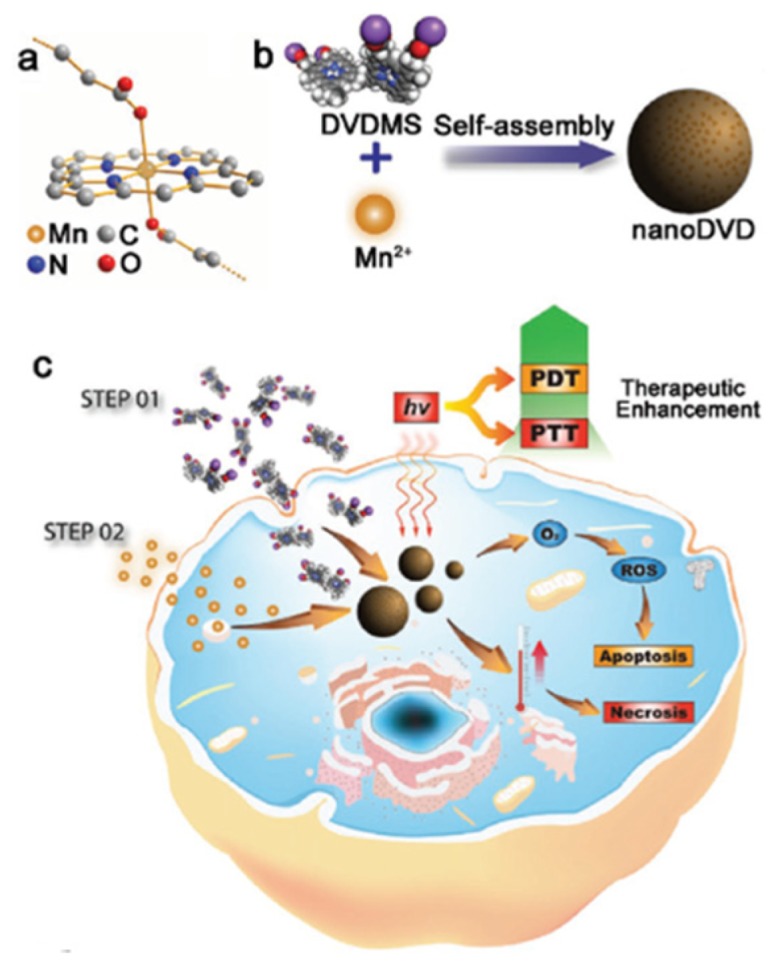
Schematic illustration showing a molecular model of the Mn^2+^ ion linking porphyrin ring and two carboxylate radicals of DVDMS molecules (**a**), the fabrication process of Mn/DVDMS (**b**), and photothermal/PDT (PTT/PDT) (c). Reprinted from Chu et al. [77] with permission from John Wiley and Sons, Copyright 2018.

Chu et al. [77] developed an engineering nanoplatform that combined many functions, including imaging (fluorescence imaging, MRI and PA imaging), as well as a synergistic combination of phototherapy (PTT and PDT). The MnO_2_ nanosheets served as a highly efficient carrier for a sinoporphyrin sodium (DVDMS) PS and an in situ O_2_ and nano-DVD (Mn/DVDMS) generator, enhanced theranostic capability of Mn^2+^-assisted assembly of DVDMS (MnO_2_/DVDMS) (Figure 15). 

MnO_2_ nanosheets served as a highly effective DVDMS carrier and in situ O_2_ and nanoDVD generator. In a tumour environment, MnO_2_/DVDMS could be reduced by GSH and H_2_O_2_, which resulted in the release of Mn^2+^, DVDMS and O_2_. In addition, GSH consumption, O_2_ production, and nanoDVD formation demonstrated an overall improved efficacy of phototherapy in vitro (630 nm, 75 mW∙cm^−2^, 5 min) and in vivo (630 nm, 300 mW∙cm^−2^, 8 min) in MCF-7 tumour. 

Gao et al. [78] developed an O_2_-generating PDT nanocomplex (IHM) by encapsulating MnO_2_ NP in HA NPs coupled to ICG. The O_2_ content in the tumour increased by 2.25±0.07 times with IHM compared without IHM treatment. After laser irradiation in the IHM group, there was a significant inhibition of tumour growth compared to the group treated with NPs. Immunofluorescent staining confirmed a decrease in both HIF1-a and pimonidazole in the IHM group, implying that generating O_2_ in the tumour not only improved the efficiency of PDT, but also weakened tumour hypoxia. In vitro studies in murine squamous SCC-7 cells showed a high suppression of cell proliferation under PDT (808 nm, 0.5 W∙cm^−2^, 10 min) treatment with IHM. In vivo in SCC-7 tumour-bearing mice highlighted a high inhibition of tumour growth with PDT treatment (808 nm, 0.8 W∙cm^−2^, 10 min) with IHM. 

Hao et al. [79] designed poly (lactide-co-glycolide) (PLGA) NPs loaded with hematoporphyrin monomethyl ether (HMME) and coated with multifunctional MnO_2_ shells, (PLGA/HMME@MnO_2_ NPs). As expected, inside the tumour they could observe the generation of O_2_ and degradation of MnO_2_ into Mn^2+^ ions by glutathione. In vitro in MCF-7 cells, under illumination (532 nm, 1.5 W∙cm^−2^, 2 min), the released HMME produced ROS that destroyed tumour cells. Furthermore, the decreased of GSH level further inhibited the consumption of the produced ROS, which greatly enhanced the PDT efficacy. These results were confirmed by in vivo studies in S180 tumour model after PDT treatment (532 nm, 1.5 W∙cm^−2^, 1.5 min) with PLGA/HMME@MnO_2_ NPs with a significant decrease of tumour volume.

Kim et al. [80] developed biocompatible mesoporous silica NPs with MnFe_2_O_4_ NPs (MFMSN) and Ce6 (MFMSN-Ce6). These results demonstrated the continuous generation of O_2_ by the Fenton catalyst under physiological conditions, improving the generation of ROS under hypoxic conditions. In vitro PDT efficiency in human glioblastoma cancer (U-87 MG) cells in both normoxic and hypoxic conditions was investigated. A significant cell death was observed in both conditions after incubation of cells with MFMSN-Ce6 and irradiated at 670 nm (0.5 W∙cm^−2^, 30 s), contrary to cells incubated with Ce6 alone and irradiated, which highlighted a low cell death in hypoxic conditions. In vivo in U87 MG-bearing mouse xenograft model, PDT treatment (670 nm, 0.88 W∙cm^−2^, 5 min) with MFMSN-Ce6 showed the best efficiency. 

Liu et al. [81] developed a bovine serum albumin (BSA) stabilized MnO_2_ NPs on which TCC (HPPH) was physisorbed (hydrodynamic size 60.52±25.61 nm). In vitro studies in U87MG cells after 630 nm laser irradiation showed that the cells incubated with the NPs exhibited more reduced viability than those incubated with the PS alone (10 mW∙cm^−2^, 1 min). In vivo (630 nm, 10 mW∙cm^−2^, 10 min) in U87MG tumour bearing mice, a complete eradication of the tumour was observed after treatment with the NPs whereas no diminution was observed with the NP without HPPH and only a low decrease with HPPH. Thanks to pimonidazole hypoxyprobe kit, they proved the decrease of tumour hypoxia.

Ma Z. et al. [82] in 2017 developed a H_2_O_2_-activatable nanostructure (SiO_2_-MB@MnO_2_) composed by a SiO_2-_MB core coated to a MnO_2_ shell. Under acidic hypoxic conditions, these NPs could release MB and generate O_2_ by decomposition of H_2_O_2_ of the TME. SiO_2_-MB@MnO_2_ exhibited a significant PDT efficiency (650 nm, 100 mW∙cm^−2^,15 min) in vitro in HeLa cells with 92% of cell death (42% for MB alone and 64% for SiO_2_-MB). Moreover, in TME conditions (pH = 5.5), an efficient release of MB by SiO_2_-MB@MnO_2_ was observed after 5h of incubation (87%). The dramatic increase of PDT effect was also proven In vivo in U14 tumour-bearing mice with an important decrease of tumour volume in comparison with irradiation of MB alone or SiO_2_-MB and a decrease of HIF-1α showing an improvement of hypoxia.

Ai et al. [83] developed upconversion nanoconjugate (UCN) loaded with Ce6 and integrating MnO_2_ NPs and HA biopolymer (UCNs-MnO_2_-Ce6-HA). Under NIR 808 nm light irradiation (0.4 W∙cm^−2^, 60 min), UCNs-MnO_2_-Ce6-HA produced high level of O_2_ by reaction of MnO_2_ with H_2_O_2_ of the microenvironment and thus led to an increase of the production of ^1^O_2_ in hypoxic conditions. In vitro studies in murine melanoma cells (B16F10) confirmed this with an increase of cell death (49%) by treatment with UCNs-MnO_2_-Ce6-HA under NIR irradiation (0.4 W∙cm^−2^, 60 min) in hypoxic conditions.

Gu et al. [84] synthesized nanocomposites (CaF_2_:Yb,Er@silica/MnO_2_-Ce6 = C@MSN-Ce6) constituted of CaF_2_:Yb,Er crystals incorporated in mesoporous silica nanospheres (MSNs) coated with a thin layer of MnO_2_ and loaded with Ce6. The MnO_2_ coating modulated a hypoxic TME by generating in situ O_2_ through a reaction with an endogenous H_2_O_2_ tumour. In vitro tests in 4T1 cells highlighted that C@MSN-Ce6 presented under light irradiation (980 nm, 0.5 W∙cm^−2^, 10 min) an improvement of PDT efficiency, in comparison with NPs without MnO_2_, in both normoxic and hypoxic conditions confirmed by an elevation of intracellular ROS level. In 4T1 tumours on Balb/c mice, in vivo studies showed a decrease of hypoxia in tumour after injection of C@MSN-Ce6 by using pimonidazole probe. The highest efficiency of PDT treatment (980 nm, 0.5 W∙cm^−2^, 30 min) was observed for C@MSN-Ce6 with a significant decrease of tumour growth.

Chen et al. [85] prepared successfully pH/H_2_O_2_-responsive carbon dots CDs/MnO_2_-PEG presenting interesting characteristics such as a low cyto toxicity and a complete clearance from the body and an increase of fluorescence and ^1^O_2_ production in TME conditons. In vitro, in HeLa cells and in vivo, in 4T1 tumour-bearing nude mice, analyses revealed that the low-toxic CDs/MnO_2_-PEG nanohybrids could be applied as pH/H_2_O_2_-driven, turn-on nanotheranostics for the concurrent bimodal MR/FL imaging and O_2_-elevated PDT of solid tumours. In vitro PDT (635 nm, 100 mW∙cm^−2^, 30 min) effect was investigated in HeLa cells under hypoxic and acidic conditions and CDs/MnO_2_-PEG exhibited high efficiency to kill cancer cells. In vivo studies, under PDT treatment (635 nm, 100 mW∙cm^−2^, 10 min) with CDs/MnO_2_-PEG, conduction a significant inhibition of tumour growth. 

He et al. [86] synthesized large pore silica nanocomposites (LPMSNs) coupled to Ce6, AS1411 aptamer for the selectivity and MnO_2_, denoted as AS1411/Ce6-LPMSNs-MnO_2_. Due to the high reactivity of MnO_2_ with endogenous acidic H_2_O_2_, massive O_2_ molecules were formed, improving PDT efficiency (660 nm, 100 mW∙cm^−2^, 10 min). 

Kapri and Bhattacharyya [87] developed a p-n heterojunction nanofilm containing p-type MoS_2_ nanoplates and nitrogen-doped reduced graphene oxide (n-rGO) with a thickness of ~ 5 nm. Illumination at 980 nm led to an effective electron-hole separation through the heterojunction. The nanoplates were modified with functionalized lipoic acid poly(ethylene glycol) to ensure better biocompatibility and colloidal stability and surface-decorated 3–5 nm MnO_2_ NPs to finally obtain pMoS_2_/n-rGO-MnO_2_-PEG nanosheets. MnO_2_ increased intracellular O_2_ by reacting with endogenous H_2_O_2_. In vitro studies in HeLa and HEK 293 (human embryonic kidney 293 cells) cells were performed highlighted an efficient PDT (980 nm, 0.4 W∙cm^−2^, 5 min) effect of the nanosheets. Compared to p-MoS_2_/n-rGO-PEG and MoS_2_/rGO-PEG, p-MoS_2_/n-rGO-MnO_2_-PEG nanoplates exhibit enhanced DCF fluorescence and they detected an increase of hypoxia due to increased apoptosis under the influence of NIR light. 

**Figure 16:**
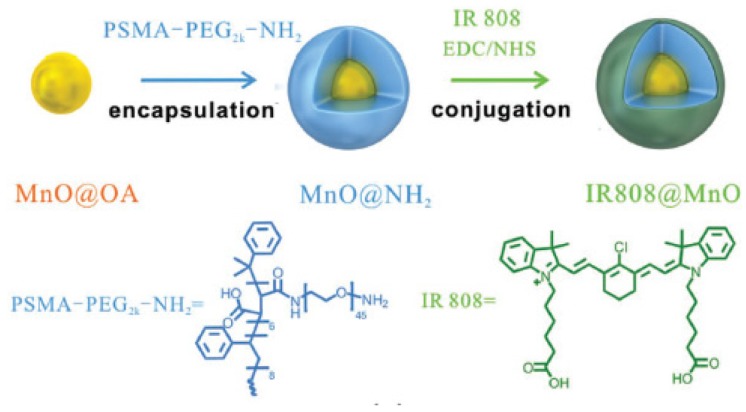
Synthetic route of MnO@OA, MnO@NH_2_, and IR808@MnO. Reprinted from [89] with permission from John Wiley and Sons, Copyright 2018.

Liang et al. [88] synthesized gold-coated NPs coupled to MnO_2_ NPs (AuNC@MnO_2_, AM). Sufficient oxygenation in the tumour site reduced local hypoxia. In addition, the O_2_-enhanced effect of PDT AM not only effectively destroyed the primary tumour in vivo, but also caused the death of immunogenic cells (ICD) with the release of damage-related molecular patterns (DAMP), which subsequently caused maturation of DC and activation of effector cells, thereby causing a systematic inducing antitumour immune responses against mTNBC. In vitro analyses in 4T1 cells showed a high increase of ROS production and cell apoptosis after treatment with AM under NIR irradiation (808 nm, 0.8 W∙cm^−2^, 3 min). In vivo studies in 4T1 tumour-bearing mice showed a high inhibition of tumour growth after AM-PDT treatment. Moreover, they observed that AM could also destroy tumour metastasis. To enhance cancer PDT, a mitochondrial-directed multifunctional MnO_2_ NP with a dye (IR808) was developed (IR808 @ MnO NP) by Zhou et al. [89] (Figure 16).

In this nanoplatform, IR808 acted as targeting ligand, eventually moving into mitochondria. Large amounts of ROS were formed during the reaction between H_2_O_2_ and MnO_2_ NPs. The results, in MCF-7 cancer cells, showed that IR808@MnO NPs improved the hypoxia environment of the tumour, generating a large amount of ROS. Moreover, a high in vitro PTT/PDT efficiency (808 nm, 0.5 W∙cm^−2^, 5 min) was observed by treatment with IR808@MnO NPs with a significant decrease of cell viability (6%) under acidic conditions. In vivo in MCF-7 tumour-bearing mice, IR808@MnO NPs highlighted a significant phototoxicity with no tumour recurrence for all treated-mice and an improvement of hypoxia in tumour.

HSA-MnO_2_-Ce6 woofers were prepared by Lin et al. [90]. HSA-MnO_2_-Ce6 NPs generated O_2_ when reacting with H_2_O_2_ at endogenous levels. In addition, ^1^O_2_ generation was doubled due to the use of HSA-MnO_2_-Ce6 NPs instead of HSA-Ce6 NPs in the presence of H_2_O_2_ with 660 nm laser illumination. In vitro mouse bladder cancer cells (MB-49) viability tests showed that HSA-MnO_2_-Ce6 NPs themselves were non-toxic, but significantly enhanced the effects of PDT on bladder cancer cells (MB-49) during laser irradiation (660 nm, 5 mW∙cm^−2^, 30 min). O_2_ content in orthotopic bladder cancer increased by 3.5 times after the introduction of HSA-MnO_2_-Ce6 NPs compared with the previous introduction of HSA-Ce6 NPs without MnO_2_. Given the superior tumour targeting and low toxicity, HSA-MnO_2_-Ce6 NPs were then used for the PDT treatment of orthotopic bladder cancer (660 nm, 200 mW∙cm^−2^, 15 min). PDA with HS of HSA-MnO_2_-Ce6 NPs showed significantly improved therapeutic efficacy and significantly increased the lifespan of mice compared with the control group.

Wang et al. [91] developed pH/H_2_O_2_ responsive nanocomplexes composed of Si QDs linked by electrostatic interaction to BSA-Ce6 to form BSA-Ce6-Si QDs (BCS) coated with MnO_2_ on the surface of BSA to obtain blocked-cell shared-memory (BCSM NPs). In the same conditions as TME (H_2_O_2_, pH = 6.5), BCSM NPs showed a high production of O_2_ and thus of ^1^O_2_ which was higher than Ce6 alone. PDT treatment (638 nm, 5 mW∙cm^−2^, 30 min) with BCSM NPs in vitro in HeLa cells conducted to a significant decrease of cell viability (20 %) and a high production of ^1^O_2_ was observed. In vivo PDT experiments in HeLa tumour-bearing mice showed that the growth of tumours was effectively inhibited under light exposure (638 nm, 5 mW∙cm^−2^, 5 min). 

Zhu et al. [92] synthesized hybrid semiconductor NPs (SPN-Ms) composed of semiconducting polymer NPs (SPN) as a core and MnO_2_ nanosheets as a shell. The SPN core served as the NIR agent for fluorescence imaging and PDT, while the MnO_2_ nanoribbons as O_2_ formation agent. SPNs were coated with different amounts of MnO_2_ and the SPN-M1 (1 *_w_*_/*w*_% of MnO_2_) generated 2.68 times more ^1^O_2_ than uncoated SPN (SPN-0) under hypoxic conditions under NIR laser irradiation (0.44 W∙cm^−2^). In vitro and in vivo in 4T1 cells model, SPN-M1 exhibited a high PDT (808 nm, 5 min, in vitro: 0.44 W∙cm^−2^ and in vivo: 0.3 W∙cm^−2^) efficiency.

Zhu et al. [93] designed a multifunctional theranostic NP (NMOFs@BSA/SDs@MnO_2_) with nanoscale metal organic frameworks (NMOFs, composed of TCPP as ligand and Fe^3+^), bovine serum albumin (BSA) and sulfadiazines (SDs), to provide actively targeting for the over-expressed carbonic anhydrase IX (CA IX) in tumour cells, and MnO_2_. NPs presented an average hydrodynamic diameter of 122 nm and its ability to produce O_2_ was also proved. In vitro in 4T1 cells, NMOFs@BSA/SDs@MnO_2_ under laser irradiation (660 nm, 50 mW∙cm^−2^, 15 min) induced a high decrease of cell viability with a production of ROS due to H_2_O_2_ decomposition. In vivo in 4T1 bearing mice highlighted a significant inhibition of tumour growth by PDT treatment (660 nm, 50 mW∙cm^−2^, 15 min) with NMOFs@BSA/SDs@MnO_2_.

Liu et al. [94] developed NPs based on black phosphorus nanocomposite as PS. The NPs (R-MnO_2_-FBP) were composed by assembly of MnO_2_ encapsulating in Rhodamine B (RhB) (R-MnO_2_) for O_2_ generator and fluorescein isothiocyanate (FITC)-labelled peptide-functionalized black phosphorus (FBP) as PS. It was shown that the O_2_ release was proportional to the liberation of Mn^2+^ and RhB in the R-MnO_2_-FBP system. Under irradiation (660 nm), R-MnO_2_-FBP presented ϕ_Δ_ of 9.9% which remained stable in hypoxic conditions. After delivery of R-MnO_2_-FBP into cancer cells in acidic and H_2_O_2_-rich environment, the generation of O_2_ was observed and PDT treatment in hypoxic cells conducted to 51.6% of cell (HeLa cells) apoptosis under laser irradiation (660 nm, 150 mW∙cm^−2^, 3 min). In vivo experiments in HeLa-bearing mice with R-MnO_2_-FBP highlighted the ability of R-MnO_2_-FBP to enhance PDT treatment (660 nm, 150 mW∙cm^−2^, 10 min).

Several teams have studied the use of MnO_2_ NPs to improve combined therapies by designing self-oxygenated systems. We report three teams who described the interest of MnO_2_ to improve PTT/PDT synergistic therapy.

Cao et al. [95] developed intelligent NPs to overcome limitations of dual PDT and PTT treatment. The NPs were constituted by a MnO_2_ nanosheet decorating at the surface with Cu_2-x_S (MnO_2_/Cu_2-x_S) and then loaded with siRNA (siRNA-small interfering RNA) to finally formed MnO_2_/Cu_2-x_S-siRNA. In an acidic microenvironment, the MnO_2_ tumour was reduced to the Mn^2+^ ion and triggered the decomposition of H_2_O_2_ to O_2_ to facilitate tumour hypoxia. Reduced Mn^2+^ ions significantly enhanced the contrast of MRI, and Cu_2-x_S acted as a powerful PA and PT imaging agent, which led to accurate trimodal tumour-specific imaging and detection. In vitro studies in MCF-7 and A549 cells were performed. Under NIR irradiation (980 nm, 0.72 W∙cm^−2^, 5 min), MnO_2_/Cu_2-x_S NPs showed a high phototoxicity with a cell viability of only 29.68% due an effective synergistic PTT/PDT effect. In vivo studies in B16 tumour-bearing mice highlighted a high inhibition of tumour growth for mice treated with MnO_2_/Cu_2-x_S-siRNA and NIR irradiation ((980 nm, 0.72 W∙cm^−2^, 5 min) proving an enhancement of PTT/PDT treatment with a high increase of ^1^O_2_ due to H_2_O_2_ decomposition by MnO_2_.

Yin et al. [96] coated uniformly carbon nanotubes (CNTs), as PTT agent with cross-linked MnO_2_ flakes, as O_2_ generator, MnO_2_/CNTs (MCs) and incubated with Ce6 to give Ce6-MnO_2_/CNTs (CMCs) for PTT/PDT therapy. This system could, in the TME, release Ce6 and CNTs while generating O_2_ by reaction of MnO_2_ with endogenous H_2_O_2_ and H^+^. In TME-mimetic conditions, the photothermal effect of CMCs and ^1^O_2_ generation under laser irradiation (PTT: 808 nm, 1.0 W∙cm^−2^ and PDT: 660 nm, 0.5 mW∙cm^−2^) was observed. In vitro in HeLa cells, CMCs showed a high phototoxicity and a strong production of ^1^O_2_ under irradiation (660 nm, 1.0 W∙cm^−2^, 5 min). The synergistic PTT/PDT effect of CMCs was highlighted in vivo in HeLa tumour-bearing mice irradiated with 808 + 660 nm (1.0 W∙cm^−2^, 10 min) with eradication of tumours. 

In 2019 Liu et al. proposed [97] new MnO_2_-based nanotherapeutic agents (honeycomb MnO_2_/IR780/BSA NPs, HMIB NPs) for NIR-activatable and self-oxygenated PTT/PDT. HMIB NPs exhibited efficient ^1^O_2_ production under NIR irradiation (785 nm, 20 mW∙cm^−2^) and PT effect (785 nm, 0.3 and 1.0 W∙cm^−2^, 6 min). In vitro in HepG2 and 3T3 cells, the authors showed that the irradiation of cells with a higher power (785 nm, 1 W∙cm^−2^) after incubation with HMIB NPs led to more cell death than irradiation at 785 nm and 50 mW∙cm^−2^ due to the PT effect. In vivo, HepG2 tumours could be completely eliminated when increasing the laser power density from 0.3 to 1 W∙cm^−2^, which could be attributed to the synergy of PDT and PTT. 

We report two studies about the use of MnO_2_ to improve chemo-PDT synergistic therapy. Chen et al. [98] synthesized HSA-MnO_2_-Ce6 & Pt NPs (HMCP) constituted by MnO_2_ nanoclusters coated to HSA modified with Ce6 or with a prodrug of *cis*-Pt (c,t,c-[Pt(NH_3_)_2_-(O_2_CCH_2_CH_2_COOH)(OH)Cl_2_] (*cis*-Pt(IV)SA)). These NPs were sensitive to pH/H_2_O_2_ using a simple one-step albumin-based biomineralization method for effective combination therapy. Once inside the tumour, MnO_2_ nanoclusters in HMCP NPs decreased hypoxia. Meanwhile, the reaction of MnO_2_ with H^+^ and H_2_O_2_ in TME led to the gradual decomposition of these albumin-MnO_2_ NPs into separate therapeutic albumin complexes with sizes less than 10 nm in order to achieve significantly improved intra-tumour interstitial diffusion for optimized treatment of tumours. As a result of these effects, large in vitro antitumour therapeutic results in 4T1 cells were achieved after combined PDT (660 nm, 5 mW∙cm^−2^, 30 min) and chemotherapy using HMCP NPs by a single treatment at a rather low dose. In vivo studies were performed in 4T1 tumours-bearing mice and a significant decrease of hypoxia was also observed in presence HMCP NPs by using pimonidazole. In vivo studies of the efficiency of HMCP combined to light irradiation (660 nm, 5 mW∙cm^−2^, 1 h) highlighted an increase of tumour growth inhibition.

**Figure 17:**
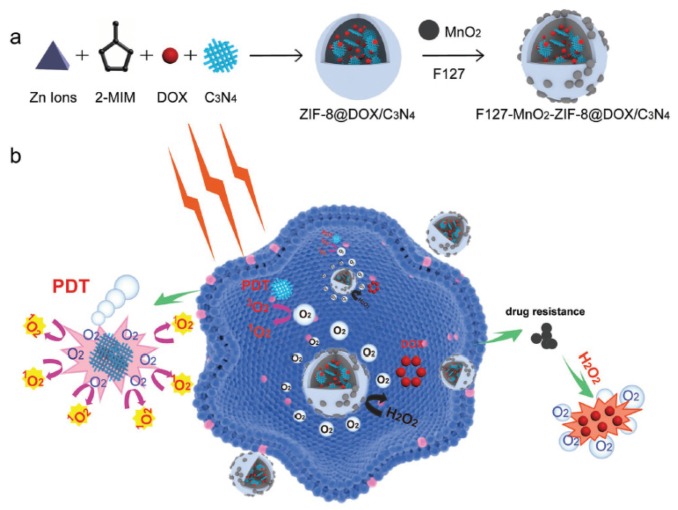
(**a**) Schematic illustration of the fabrication of FMZ/DC nanocomposites. The diagram is not drawn to scale. (**b**) Schematic illustration of FMZ/DC with O_2_ generation enhancing the chemo-PDT under 660 nm light irradiation. Reprinted from [99] with permission from John Wiley and Sons, Copyright 2018.

Zhang et al. [99] developed an O_2_-generating and pH-sensitive nanoplatform, loading MnO_2_ for O_2_ generation, g-C_3_N_4_ for PDT, ZIF-8 (zeolitic imidazolate frameworks) for drug encapsulation and doxorubicin hydrochloride for chemotherapy (Figure 17).

In vitro experiments in 4T1 cells showed a higher therapeutic effect using the NPs compared to therapy without MnO_2_ nanodot in hypoxic conditions or only with chemical and PDT (660 nm, 5 mW∙cm^−2^, 30 min) with the presence of MnO_2_ nanodot. In vivo experiments also showed that NPs could reduce tumour hypoxia and improve the effect of photodynamic and chemotherapeutic treatment. 4T1 tumours could be very effectively eliminated using the developed NPs when irradiated with light (660 nm, 5 mW∙cm^−2^, 30 min). 

Two others studies about the use of MnO_2_ to improve combined therapies have been described.

**Figure 18:**
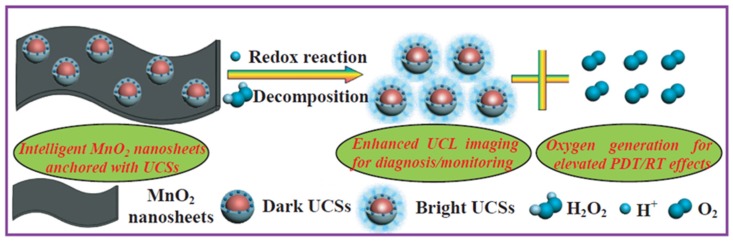
Schematic illustration of the decomposition of MnO_2_ nanosheets arising from the redox reaction between UCSMs and acidic H_2_O_2_, which led to the enhanced UCL imaging for diagnosis/monitoring as well as the massive O_2_ generation for improving the synergetic PDT/RT effects. Reprinted from [100] with permission from John Wiley and Sons, Copyright 2015.

Fan et al. [100] were the first to propose intelligent MnO_2_ nanosheets, anchored with UCNPs, to simultaneously visualize and treat solid tumours after X-ray/light excitation. After NIR illumination of the quenched UCNP, luminescence of the UCNPs was enhanced after the decomposition of MnO_2_ into Mn^2+^ by acidic H_2_O_2_ in the tumours (Figure 18). 

Hypoxic murine breast cancer, hc-4T1 cells, were used. No killing could be observed after X-ray (5 Gy, 5 min) or light irradiation (NIR, 1.5 W∙cm^−2^, 5 min). After addition of the compound, cell death could be observed after PDT (UCSMs + NIR) and RT (UCSMs + RT) due to the O_2_ generation. Moreover, after NIR and X-ray, the lowest cell viability would be detected showing the synergetic PDT/RT effects. The same effect was detected in vivo in 4T1 solid tumour (PDT: NIR, 2 W∙cm^−2^, 10 min and RT: 8 Gy, 5 min). In vivo PA imaging was performed to evaluate the vascular saturated O_2_ by evaluating oxygenated and deoxygenated Hb. Injection of UCSMs increased the signal intensity of oxygenated/deoxygenated Hb but also enhanced O_2_ by about 7%. Moreover, addition of UCSMs induced a down-expression of HIF-1α, due to the decreased hypoxia and increased oxygenation. 

He et al. [101] proposed a glutathione-activatable and O_2_/Mn^2+^-Evolving NPs for both PDT and gene-silencing therapy (GAOME N). This system consisted of the MnO_2_ cellular nanocarrier (hMnO_2_) loaded with catalase, Ce6, and foliar-tag DNAzyme. After endocytosis, hMnO_2_ carriers were reduced by overexpressed GSH to Mn^2 +^ ions, which led to a decrease of GSH level and the breakdown of GAOME NC. Then, the released catalases triggered the cleavage of endogenous H_2_O_2_ with the formation of O_2_. The formation of O_2_ and the consumption of GSH effectively increased the efficiency of PDT. In addition, the DNAzyme was released to silence the genes in the presence of self-generated Mn^2 +^ ions as cofactors. Rational synergy of enhanced PDT and gene therapy led to a significant increase in the effectiveness of cancer treatment in vitro (MCF-7 cell line, 635 nm, 30 mW∙cm^−2^, 5 min) and in vivo (MCF-7 tumour-bearing mice, 635 nm, 100 mW∙cm^−2^, 5 min). 

Another type of NP was able to catalyse H_2_O_2_ to produce O_2_ as well as being a contrast agent for both MRI and fluorescence imaging: carbon dot [102]. Jia et al. synthesized Mn-carbon dots (CDs) by a solvothermal approach with Mn(II) phthalocyanines (Mn-Pc) and cooperative self-assembly with DSPE-PEG. The hydrodynamic diameter of this Mn-CD assembly was around 162 nm. Mn(II) could be oxidized to MnO_2_ that led to the catalytic decomposition of H_2_O_2_. Formation of O_2_ was observed when Mn-CD was in acidic H_2_O_2_ solution whereas no O_2_ was produced in the absence of H_2_O_2_ or Mn-CD. In acidic media, the O_2_ bubbles were even more abundant. Mn-CD assembly totally disassembled in acidic H_2_O_2_ solution forming free Mn-CD. By ESR technique in solution and using SOSG probe in vitro in HeLa cells, they proved the formation of ^1^O_2_ even in hypoxic conditions. For PDT experiments (635 nm, 50 mW∙cm^−2^, 10 min) the cells were cultured in an acid medium (pH = 6.5) with addition of H_2_O_2_. 99% mortality rate could be observed whereas with CD without Mn, no PDT effect could be observed (no PS). In vivo PDT efficiency was evaluated in subcutaneous 4T1 tumour-bearing female nude mice (635 nm, 50 mW∙cm^−2^, 10 min). With Mn-CD the tumour was completely inhibited and no recurrence could be observed. All the mice survived over 60 days (Table 6).

#### 3.1.2. Catalase

Catalase is a natural enzyme. Catalase has a specific catalytic behaviour and is able to decompose endogenous H_2_O_2_ to produce O_2_ bubbles in the tumour tissue. It can then be used to regulate tumour hypoxia. However, it is not very stable in the presence of proteases in the blood circulation and systemic administration is compromised. In order to protect catalase, one solution can be its encapsulation or coupling to inorganic or organic NPs. That will be the aim of the next paragraph. 

As far as we know, the first paper developing this strategy was written by Chen et al. [103]. They synthesized multifunctional NPs composed of catalase (encapsulation efficiency 13.1%) into a PLGA (poly(D,L-lactic-co-glycolic acid) NP with MB as a PS (encapsulation efficiency 19.51%), a black hole quencher-3 (BHQ-3) as an energy quencher of excited PS and a targeting unit (RGDfK) for α_v_β_3_ integrin targeting. The average hydrodynamic diameter of the HAOP NP was 205 nm measured by DLS. In the presence of H_2_O_2_, formation of O_2_ bubbles induced the destruction of the HAOP NPs shell and the release of MB. Using SOSG, they also demonstrated the formation of ^1^O_2_ in the presence of H_2_O_2_ after 635 nm excitation (100 mW∙cm^−2^, 5 min) of the HAOP NPs_,_ which was negligible for HAOP NPs without H_2_O_2_ and NPs without catalase. PDT efficacy was performed with light of 635 nm (100 mW∙cm^−2^, 5 min) on U87-MG cell line. After illumination, the apoptotic cells were 98.2% for cells treated with HAOP NPs and only 15.8 % for cells treated with HAOP NPs without catalase. A lysosome-associated pathway could be concluded using acridine orange as a marker of lysosome integrity. A commercially available O_2_ probe ([Ru(dpp)_3_]Cl_2_) allowed to detect intracellular O_2_ which increased after incubation with HAOP NPs. The group treated with HAOP NPs without catalase showed high level of HIF-1α whereas the group treated by HAOP NPs showed very week level expression due to generated O_2_ that overcame the hypoxia. In vivo PDT was realized on BALB/c mice bearing U87-MG tumour. As expected after HAOP NPs injection, the tumour growth was totally inhibited which was not the case for all the control groups. After release from the NPs, catalase could generate high level of O_2_.

Zhang et al. in 2016 [104] designed core-shell gold nanorods (APMECR@MB NC) (diameter of 75 nm) covered with phenyl mesoporous silica loaded with [Eu(THA)_3_(phen)] to trigger MB via luminescence resonance transfer, a catalase was covalently coupled to the surface via its amino group and conjugated to a targeting peptide (cRGD). Thanks to the overlap between emission spectrum of Eu(THA)_3_(phen) and absorption spectrum of MB, high energy transfer could happen upon 808 nm excitation between Eu(THA)_3_(phen) and MB estimated to be 96.1%. With DPBF, it was possible to conclude to the formation of ^1^O_2_. APMECR@MB NC produced O_2_ bubbles in the presence of H_2_O_2_. Prostatic cancer cell lines (PC-3) were cultured in hypoxic incubator. Using DCFH-DA, ^1^O_2_ could be detected after incubation with APMECR@MB NC whereas little or no ^1^O_2_ could be detected after incubation with free MB or APME@MB without catalase. Pork tissues of various thickness were used and placed between the excitation source (808 nm, 1000 mW∙cm^−2^ for 5 min) and the PC-3 cells. The authors proved that there was a synergistic PTT/PDT of PC-3 cells with the use of APMECR@MB.

**Figure 19:**
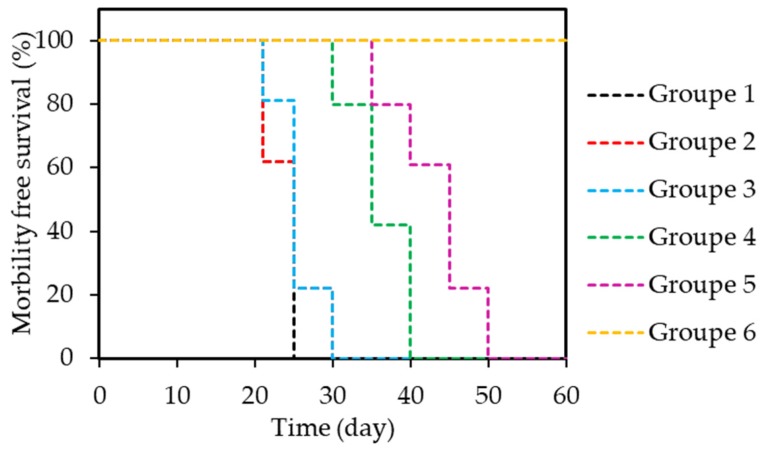
In vivo PDT in xenograft mice models with SW1990 PC. Survival rates of mice bearing SW1990 tumours after different treatments. (*) *p* < 0.05, (**) *p* < 0.01. Group 1-PBS, Group 2-laser, Group 3-ZCM nanocapsule, Group 4-free MBlaser, Group 5-zeolite-MB-laser, and Group 6-ZCM nanocapsule-laser (n = 5 per group). Adapted from Hu et al. [105].

Hu et al. [105] used a zeolite with mesoporous structure (531 nm) as a nanocarrier to immobilize catalase and MB (ZCM NPs). The catalase loaded in the zeolite was still efficient and kept 90% of enzymatic activity compared to free catalase. As expected, in closed tubes, it was possible to detect O_2_ formation in the presence of ZCM NPs and H_2_O_2_. In vitro detection of ^1^O_2_ with SOSG revealed that the ^1^O_2_ formation was 3.7 higher for the ZCM treated group than the group’s zeolite or zeolite-MB. Formation of O_2_ upon decomposition of in situ H_2_O_2_ thanks to the presence of the NPs was 4.8-fold higher than without NPs and allowed the release of MB. The PDT experiments were performed on athymic nude mice with SW1990 with laser at 635 nm, 50 mW∙cm^−2^ for 10 min. They could observe a total inhibition of tumour growth at 18 days, which was not possible under other conditions (control, light only, ZCM only) (Figure 19).

Cheng et al. [106] described catalase incorporated into a nanoscale metal-organic framework (NMOF) composed of a cancer cell membrane and a cytoskeleton-like porous zeolitic imidazole framework with AlPcS_4_ (Al(III) phthalocyanine chloride tetrasulfonic acid) (10.6 %). The core-shell structure had size of about 110 nm and an outer lipid bilayer shell of 7.2 nm. After irradiation at 660 nm (30 mW∙cm^−2^, 1 min) of HeLa cells in presence of CAT-PS-ZIF@Mem, 75.6 % of the cells died whereas only 12.28% died when exposed to PS-ZIF@Mem without catalase. BALB/c-nu mice with HeLa cells were used as tumour model. HIF-1α staining assay was used to evaluate the tumour hypoxic conditions. As expected the group treated with CAT-PS-ZIF@Mem presented low signal (low hypoxia) whereas the group treated with PS-ZIF@Mem without catalase presented high signal. For PDT experiments (660 nm, 220 mW∙cm^−2^, 5 min) the tumour growth was significantly inhibited showing that the CAT-PS-ZIF@Mem NPs have a high phototoxicity and that the formation of O_2_ thanks to decomposition of H_2_O_2_ improved PDT efficiency.

Combination therapy is also possible. Chen et al. [72] described self-assembly of HSA in which they covalently coupled Ce6 (2.6 %) and encapsulated catalase and a chemotherapeutic drug the paclitaxel (PTX) (6.5 %). The NPs HSA-Ce6-Cat-PTX showed spherical structure of around 100 nm of diameter. They proved that HSA-Ce6-Cat-PTX NP produced more O_2_ in the presence of H_2_O_2_ than HSA-Ce6-Cat may be due to some loss of activity of the catalase in HSA-Ce6-Cat. In HSA-Ce6-Cat-PTX NPs, the entrapped catalase maintained around 70% of its initial enzymatic activity. Moreover, the generation of ^1^O_2_ by HSA-Ce6-Cat-PTX detected with SOSG was higher in the presence of H_2_O_2_ due to the additional O_2_ supply. In vitro experiments were performed in 4T1 cells with 660 nm light (5 mW∙cm^−2^, 30 min). Compared with PDT alone, chemotherapy alone or both, the combination treatment under light irradiation proved to be the best. After light illumination, the release of PTX into the cytoplasm improved the PDT effect. Under nitrogen atmosphere and addition of HSA-Ce-cat was not able to induce PDT effect whereas HSA-Ce6-Cat-PTX had a very good cancer cell killing efficiency. Female nude mice bearing 4T1 tumours were used for in vivo tests. To check the hypoxia into the tumour, (HIF)-1α and pimonidazole were used. Both HSA-Ce6-Cat-PTX and HSA-Ce6-Cat showed significant decreased hypoxia signals compared to the control. After PDT (660 nm, 5 mW∙cm^−2^, 60 min) the most effective tumour growth inhibition was observed for mice treated with HSA-Ce6-Cat-PTX compared to PDT alone or chemotherapy alone. To sum up, HSA-Ce6-Cat-PTX NPs accumulated into the tumour then decomposed, PTX was released, catalase decomposed endogenous H_2_O_2_ to produce O_2_, PDT induced the disruption of lysosomes and the PTX distributed into cytoplasm.

Yang et al. [107] also designed a very smart multifunctional NPs in which they encapsulated catalase in hollow silica NPs, with covalently coupled Ce6, covalently coupled CTPP (3-carboxypropyl)triphenylphosphonium bromide) to target mitochondria and modified with an acidic pH responsive charge-convertible DPEG polymer (in acidic conditions, the DPEG polymer had positives charges that enhance tumour cell internalization) and programmed death-ligand 1 (PD-L1) antibody to promote the infiltration of T lymphocytes in metastasis. The obtained CAT@S/Ce6-CTPP/DPEG had a size of around 100 nm with a shell thickness of 12 nm. Comparing O_2_ formation in solution in the presence of H_2_O_2_, they could observe a rapid O_2_ formation for CAT@S/Ce6-CTPP/DPEG compared to the control BSA@S/Ce6-CTPP/DPEG. CAT@S/ Ce6-CTPP/DPEG kept 70% of its initial activity compared to free catalase in the presence of protease. Thanks to SOSG, they also observed the increase in ^1^O_2_ formation in solution with CAT@S/Ce6-CTPP/DPEG excited at 660 nm in the presence of H_2_O_2_ compared to the absence of H_2_O_2_. 4T1 cells were used for in vitro tests (660 nm, 5 mW∙cm^−2^, 60 min). The cell killing efficiency was higher after incubation with CAT@S/Ce6-CTPP/DPEG compared to BSA@S/Ce6-CTPP/DPEG. Confocal microscopy imaging revealed a specific intracellular targeting of mitochondria. Oxygenation level was increased with CAT@S/Ce6-CTPP/DPEG compared to the control and the use of pimonidazole proved the decrease of hypoxia. PDT efficiency was also the best for CAT@S/Ce6-CTPP/DPEG (660 nm, 5 mW. cm^−2^, 60 min). Very interestingly, thanks to the programmed death-ligand 1 (PD-L1) antibody, they showed that PDT plus anti-PD-L1 treatment was able to stop the progression of tumours that were not irradiated (1–2 cm away).

Shen et al. [108] synthesized pH-responsive aerobic NPs made of interaction of negatively charged catalase with positively charged chitosan. The resulting micelles (C&C) were used to encapsulate Ce6 and the size of the C&C-Ce6 was 68.5 nm. In acidic environment, the disassembly of the NP led to the release of Ce6 (75% after 80 h) as well as the release of catalase. They showed that the formation of ^1^O_2_ after addition of H_2_O_2_ was higher in acidic media due to the release of both Ce6 and catalase in contact of H_2_O_2_. In vitro phototoxicity was evaluated in squamous cell carcinoma CAL-27 (650 nm, 100 mW∙cm^−2^, 10 min). IC_50_ was found to be 15.33 mg·mL^−1^ for free Ce6 and 12.02 mg∙mL^−1^ for catalase + Ce6 mixture and only 4.03 mg∙mL^−1^ for C&C-Ce6. The percentage of apoptotic cell after PDT treatment for PBS, Ce6, chitosan + Ce6, catalase + Ce6 and C&C-Ce6 NPs were respectively 9.38%, 78.57%, 76.7%, 86.09% and 94.31% showing the important role of catalase that induced higher formation of ^1^O_2_. Female BALB/c mice bearing CAL-27 tumour (650 nm, 100 mW∙cm^−2^, 10 min) were chosen for in vivo experiments. After 14 days after the treatment, the tumour volumes for PBS, Ce6, chitosan + Ce6, catalase + Ce6 and C&C-Ce6 NPs were respectively 1.87±0.14 cm^3^, 1.04±0.09 cm^3^, 0.99±0.15 cm^3^, 0.96±0.12 cm^3^ and 0.52±0.11 cm^3^, in good agreement with in vitro results. The lowest amount of hypoxia-inducible factor HIF-1α was found as expected for the C&C-Ce6 NPs treated mice.

Zhao et al. [109] described graphitic-phase mesoporous carbon nitride nanosheet (mpg-C_3_N_4,_ MCNs) in which hematoporphyrin monomethyl ether (HMME) and catalase were wrapped into the 5 nm pores. HA was covalently coupled to the nanosheet. They checked that catalase still kept its activity when it was encapsulated. The best HMME loading was obtained with a feed rate HMME/MCNs of 3/1. A strong incorporation of HA@mgp-C_3_N_4_-HMME/CAT into murine melanoma B16-F10 cell lines could be observed by confocal microscopy and the apoptosis rate was higher than all the others conditions ((HA@mgp-C_3_N_4_ + LED_,_ HMME, HA@mgp-C_3_N_4_-HMME, HA@mgp-C_3_N_4_-HMME/CAT) after light illumination (LED, 3000 mW∙cm^−2^ for 2 min). In female C57 mice, the in vivo results were in good agreement with the in vitro ones, the authors could detect enhanced inhibition of tumour growth after injection (i.v.) of HA@mgp-C_3_N_4_-HMME/CAT, and light illumination compared to all the others groups. Using pimonidazole, reduced hypoxia could be detected in tumours from mice injected with HA@mgp-C_3_N_4_-HMME/CAT thanks to the formation of O_2_ through the decomposition of H_2_O_2_ compared to mice injected with HA@mgp-C_3_N_4_-HMME and irradiated.

Another idea is to target proinflammatory-activated macrophages that are involved in chronic inflammation and also known to generate H_2_O_2_ to destroy undesirable invaders [110]. Lu et al. [111] developed selenium NPs (SeNPs) coated with a first layer of chitosan(CS)-RB-GSH, a thiolate catalase (CAT) covalently coupled to this first layer and a second layer of HA-ethylene diamine(EDA)-folic acid(FA). HA and FA were respectively CD44 and FR-b targeting agent over-expressed on cell surfaces of activated macrophages. The size of the NPs was 148.4±7.2 nm. ^1^O_2_ detection was performed by electronic paramagnetic resonance (EPR) and using 9,10-diphenylanthracene (DPA) and was more important in the presence of H_2_O_2_. The NPs presented high specificity for activated macrophages compared to normal macrophage. In RAW 264.7 (mouse macrophages) and under light illumination (green LED array, 40 mW∙cm^−2^, 10 min) a decrease of cell viability could be observed (43.3% cell viability) with SeNPs and 32.8% with CAT/SeNPs. Using CAT/SeNPs led to H_2_O_2_ depletion and inhibition of activation and proliferation of macrophages.

Ouyang et al. [112] proposed the use of thylakoid membrane which was able to catalycally decompose H_2_O_2_ to produce O_2_ thanks to its membrane-related catalase [113]. With mechanical grinding, chloroplasts were extracted from spinach leaves. They underwent hypotonic lysis to release thylakoids. After extrusion, nanovesicle biomimetic nanothylakoids (NT) were obtained with a membrane thickness of around 9 nm and a size of around 50 nm. In hypoxic conditions and in the presence of H_2_O_2_ the dissolved O_2_ concentration increased in solution and H_2_O_2_ was catalytically decomposed. Using SOSG, after 660 nm light excitation (1 W∙cm^−2^, 5 min), 7.8 more ^1^O_2_ was produced within 5 min. NP without catalase (LP) was synthesized as a control. In hypoxic environment, NPs produced larger amount of ^1^O_2_ than LP. In 4T1 cancer cells, [Ru(dpp)_3_]Cl_2_ quenched fluorescence in hypoxic environment proved the formation of O_2_ and SOSG the formation of ^1^O_2_. In vivo studies on tumour-bearing mice injected with NT or LP were performed. The expression of HIF1-a decreased after NT treatment which was not the case with LP injection, suggesting that tumour hypoxia disappeared after NT treatment. After PDT (660 nm, 1 W∙cm^−2^, 10 min) the tumour growth was totally inhibited after injection of NT whereas with LPs, the tumour growth was only delayed within the first 4 days and even recurred later.

**Figure 20:**
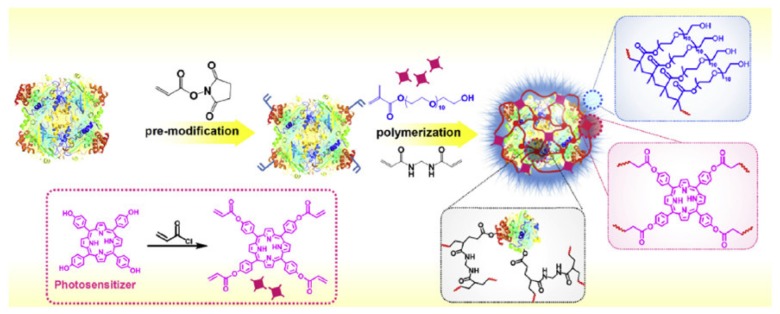
Schematic illustration of the preparation and structure of CAT-THPP-PEG. Hydrodynamic diameters of catalase, BSA-THPP-PEG and CAT-THPP-PEG in water, PBS and FBS. Reprinted [114] with permission from Elsevier Ltd, Copyright 2018.

An original strategy was developed by Wang et al. [114]. Catalase could lose a part of its activity when encapsulated into NPs. In this paper, they used the acrylate onto the surface of the protein to enable free radical polymerization of a small PEG as the drafting moiety and *Meso*-tetra(*p*-hydroxyphenyl)porphine (THPP) as a cross linker. The CAT-THPP-PEG nanocapsules showed spherical morphology with a size of around 25 nm and 30 nm by DLS (Figure 20).

Using SOSG, after light illumination (660 nm, 5 mW∙cm^−2^, 30 min) they could detect in solution a high production of ^1^O_2_ in the presence of H_2_O_2_ both in normoxic and hypoxic media due to the formation of extra O_2_. In vitro experiments in 4T1 cells were performed (660 nm, 5 mW∙cm^−2^, 30 min). As expected, cell killing increased under hypoxic conditions in presence of CAT-THPP-PEG and not in presence of the control BAS-THPP-PEG. Antipimonidazole antibody was used to detect hypoxia zones on tumour slices and revealed a large decrease of tumour hypoxia after CAT-THPP-PEG treatment compared to BSA-THPP-PEG treatment. In vivo studies in BALB/c mice bearing 4T1 tumours (660 nm, 5 mW∙cm^−2^, 60 min) confirmed the in vitro studies with a large inhibitory effect of the tumour growth after CAT-THPP-PEG injection.

Another strategy is not to use natural enzyme but nanomaterial-based artificial enzyme. They are called nanozyms. The advantage is that the requirement of enzyme encapsulation is not needed neither the controllable release.

In 2017 Liu et al. [115] developed peroxidase amine-terminated PAPAM dendrimer-encapsulated gold NP with catalase like activity (AuNCs-NH_2_) and coupled to Protoporphyrin IX (PpIX). They proved that the AuNCs-NH_2_ NPs possessed catalase-like activity at pH 4.8–7.4 that could allow H_2_O_2_ consumption to form O_2_ in cytoplasm (pH = 7.4), endosomes (pH = 5.5) or lysosomes (pH = 4) Evidence of O_2_ formation was obtained in lung cancer H 460 cells. PDT efficiency (532 nm, 200 mW∙cm^−2^, 5 J per well) in hypoxic and normoxic conditions with AuNCs-NH_2_ and PPIX showed that AuNCs-NH_2_ overcome cancer cell hypoxia and induced a better PDT efficacy.

Yang et al. [116] in 2018 described a new kind of catalase-mimeting DNAzyme function NP with AN1411 DNA G-quadruplex/hemin (iron-containing porphyrin)-Ce6 coordinated to calcium ions and surrounding by poly(L-histidine)polyethylene glycol. AS1411/Ce6 (AC) and AS1411/hemin (AH) complexes had a size of 10 nm. Thanks to Ca^2+^ coordination, and poly(L-histidine)- polyethylene glycol (pHis-PEG) as stabilizing agent, the CACH-PEG NP was elaborated with a size of 70 nm. CACH and CACH-PEG NP were more efficient in producing ^1^O_2_ in the presence of H_2_O_2_ under 660 nm light irradiation than free Ce6. At acidic pH (5.5), the hydrodynamic size of CACH-PEG NP decreased to 12 nm showing the formation of smaller G-quadruplex. In 4T1 cells, the CACH-PEG NP internalized via endocytosis and Ce6 localized in the nuclei. PDT treatment (660 nm, 5 mW∙cm^−2^, 30 min) with or without H_2_O_2_ was conducted and as expected, the level of ^1^O_2_ produced was higher in the presence of H_2_O_2_. In BALB/c mice bearing 4T1 tumours, ^99m^Tc CACH-PEG NPs showed tumour retention (5.4% of injected dose per gram of tissue (%ID/g)). Using pimonidazole proved that hypoxia was reduced in the mice that have been injected with PEG CACH-PEG NPs. After PDT (660 nm, 5 mW∙cm^−2^, 60 min) the best inhibition of tumour growth was observed for mice treated with CACH-PEG NP.

Ruan et al. [117] synthesized manganese-iron layered double oxide (MnFe-LDH) that possessed catalase-like activity and was loaded with MB. The particle size was about 200 nm and thickness of 2.8 nm with 2–3 layers. Under 808 nm laser with high irradiance (1000 mW∙cm^−2^) for 10 min, the temperature increased rapidly. In HeLa cells incubated with MnFe-LDH, they detected the formation of O_2_ by checking the quenched fluorescence of [Ru(dpp)_3_]Cl_2._ In solution using DPBF, ^1^O_2_ formation after 650 nm light excitation of MnFe-LDH/MB could be detected and addition of H_2_O_2_ in the solutions increased ^1^O_2_ formation. With SOSG in HeLa cells, the same results were obtained. The irradiation of HeLa cells (650 nm, 100 mW∙cm^−2^ for 10 min) with free MB and MnFe-LDH/MB led to respectively 15% and 40% of cell death. After irradiation at 808 nm, 51 % cell death was observed. After 650 nm and 808 nm irradiation, cells treated with MnFe-LDH/MB all died showing the synergistic effect of PTT and PDT. In female Kunming mice with murine cervical cancer cells (U14) (650 nm, 100 mW∙cm^−2^ for 15 min and 808 nm 1000 mW∙cm^−2^ 10 min) tumour growth inhibition was moderate after PTT or PDT, whereas the dual treatment induced an almost total destruction of the tumour. HIF-1α was very low. 

Two papers utilized Prussian Blue (PB) NPs that are known for their catalase-like activity and were FDA approved for clinical application [118,119]. In the first one, Yang et al. [119] described periodic mesoporous organosilica coated with PB NPs and loaded with Ce6. TESPTS (1,4-bis(triethoxysily)propane tetrasulfide was used as organosilica molecule to coat PB NPs. The hydrodynamic diameter of PB@PMO-Ce6 NPs was about 170 nm. In solution, the mixing of PB@PMO-Ce6 and H_2_O_2_ resulted in the formation of O_2_ bubbles showing the catalase-like activity of PB@PMO-Ce6 NPs. In U87MG cells, after irradiation at 660 nm (1000 mW∙cm^−2^, 5 min), total amount of formed ^1^O_2_ was higher for PB@PMO-Ce6 in the presence of H_2_O_2_ than with PB@PMO-Ce6 alone. PDT in vivo was investigated in U87MG tumour-bearing mice by intratumoural injection of PB@PMO-Ce6 or Ce6 alone and 660 nm light (1000 mW∙cm^−2^, 2 min). Higher formation of ^1^O_2_ and apoptotic cells were observed after PB@PMO-Ce6 treatment than Ce6 treatment. Tumour growth presented a slower rate after Ce6 treatment and was totally broken after PB@PMO-Ce6 treatment.

**Figure 21:**
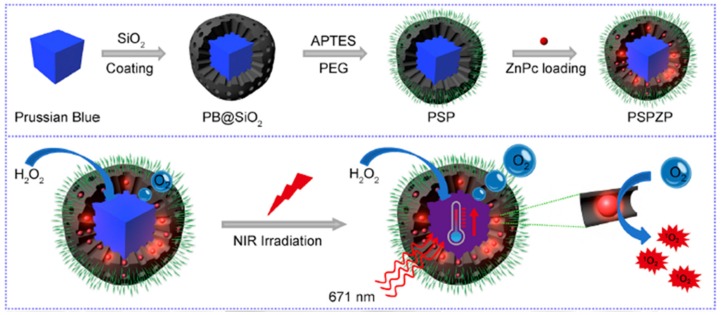
Schematic of the synthetic procedure and photo-enhanced therapy of the PSPZP NCs. Reprinted from [118] with permission from Elsevier Ltd, Copyright 2018.

The second paper concerns Wang’s work [118] who used mesoporous silica to coat PB, coated by PEG and loaded by Zinc phthalocyanine (ZnPc) (Figure 21). The NPs with a PB core and SiO_2_ shell surrounding by PEG had a hydrodynamic diameter of around 140 nm (PSP NCs). More than 50% H_2_O_2_ was degraded in the presence of PSP NCs within 1 h at 25 °C with the apparition of O_2_ bubble and increased at 43 °C. The concentration of O_2_ formed was 1.95 time higher at 43 °C than at 25 °C. 

For each PSP particle, it was possible to load 18,800 ZnPc (PSPZP). Using DPBF in hypoxic condition a significant increase of ^1^O_2_ formation was observed after addition of H_2_O_2_. 4T1 cells were incubated under hypoxia conditions with ([Ru(dpp)_3_]Cl_2_. After being treated with PSP NCs, the decrease of the fluorescence of ([Ru(dpp)_3_]Cl_2_ proved the formation of O_2_. HeLa, A549 (Human lung cancer cells) and 4T1 cells were selected for PDT experiments (671 nm, 400 mW∙cm^−2^ for 5 min). After incubation with PSPZP, the cytotoxicity was higher in normoxic than hypoxic conditions. Addition of H_2_O_2_ in hydroxic condition increased cytotoxicity that was even enhanced at 43 °C. In vivo experiments were performed in 4T1 tumour-bearing mice (671 nm, 400 mW∙cm^−2^, 5 min) after intravenous injection of PSPZP. The growth of the tumour stopped which was not the case for PSPZP without light, light, ZnPc + light, PSP + light. The reduced pimonidazole fluorescence in tumour slices extracted from mice treated with PSPZP showed that the tumour hypoxia disappeared. The drawbacks were the low accumulation and the lack of targeting unit.

Two papers have described the use of Fe(III) for its catalase like-activity. Ma et al. showed that Fe(III) could have catalase-like activity and could decompose H_2_O_2_ to create O_2_ [120]. They elaborated Fe(III) doped Graphitic-phase C_3_N_4_ nanosheet covalently coupled to a mitochondria- targeting moiety—(4-carboxybutyl)triphenylphosphonium bromide (TPP)—and loaded with MB on the surface. The C_3_N_4_-Fe-TPP NF/MB NPs in water had a size of 110 nm that moved to 116 nm after 5 days. Fluorescence of [Ru(dpp)_3_]Cl_2_ after addition of H_2_O_2_ to C_3_N_4_-Fe-TPP NF/MB NPs solution suggested an increase of local O_2_ concentration. With DPBF, formation of ^1^O_2_ was proven under 650 nm light illumination (100 mW∙cm^−2^) and in vivo in HeLa cells (650 nm, 100 mW∙cm^−2^, 15 min) with DCHF-DA. PDT experiments in vitro in HeLa cells showed a slight decrease of cell viability with free MB and C_3_N_4_-NS/MB NPs without Fe(III) whereas a strong decrease of cell viability was observed after incubation with C_3_N_4_-Fe NF/MB NPs and C_3_N_4_-Fe-TPP NF/MB NPs. The in vivo efficiency was determined in U14 cervical cancer in Kunming mice (650 nm, 100 mW∙cm^−2^, 15 min each day, 2 days interval). C_3_N_4_-Fe-TPP NF/MB NPs proved to be very efficient and led to almost complete elimination of the tumour with no regrowth during the 15 days of the experiment. HIF-1α indicated a higher O_2_ concentration in the group treated with C_3_N_4_-Fe NF/MB and C_3_N_4_-Fe-TPP NF/MB NPs.

Few months later, Zhen et al. [121] also used the catalase-like activity of Fe(III) and developed BSA that coordinated with Fe^3+^ through different active groups such as carboxyl, thiol amino. MB was then added and loaded in BSA through amide bond and/or hydrophobic interaction. The monodispersed MB-BSA-Fe(III)NP had a hydrodynamic size of 28.21 nm. In solution, the production of O_2_ was measured in the presence of MB-BSA-Fe(III)NP and H_2_O_2_ suggesting that MB-BSA-Fe(III) possessed catalase-like activity. In solution under light illumination (650 nm, 100 mW∙cm^−2^ 10 min) the absorbance of DPBF (proportional to ^1^O_2_ formation) decreased to 79% with H_2_O_2_ and only 60% without H_2_O_2_. In HeLa cells, the fluorescence of Ru(dpp)_3_]Cl_2_ disappeared after 24 h in cells treated with MB-BSA-Fe(III)NP suggesting that NPs could overcome the hypoxia. After PDT (650 nm, 100 mW∙cm^−2^, 15 min) viability was 83.3% after MB incorporation whereas a high decrease of 33.7% was observed with MB-BSA-Fe(III)NP. 

Metal-organic frameworks (MOFs) are self-assembled molecules that have nanometric size and present excellent properties such as high surface-to-volume ratio, large pore size and pore windows. Two papers in 2018 used MOFs. Hu et al. [122] used iron(III) carboxylate MOF (MIL-100 (Fe); MIL stands for Materials Institutes Lavoisier) as a carrier for three kinds of PS: Ce6, 2-((4’-(2,2-bis(4-methoxy- phenyl)-1-phenylvinyl)-1,1’-biphenyl-4-yl)(phenyl)methylene)malonitrile (TPEDC) and (*E*)-2-(4-(4-(2,2-bis(4-methoxyphenyl)-1-phenylvinyl)styryl)-3-cyano-5,5-dimethyl- furan- 2(5*H*)-ylidene)malonitrile (TPETCF). To improve the water dispersibility, they coated the NPs with a nonionic amphiphilic block copolymer F127. The average hydrodynamic size was 140 nm (size distribution 90–400 nm). In solution and in the presence of H_2_O_2_, a catalytic reaction occurred thanks to Fe(III) leading to the release of free Fe(III) that will decomposed rapidly H_2_O_2_ to produce O_2_. 9,10-Diphenylanthracenediyl-bis(methylene) dimalonic acid (ABDA) was used to prove that PSs did not produce ^1^O_2_ when trapped into the MOF, but were still able to produce ^1^O_2_ when they escaped. F127-TPETCF@MIL-100 was chosen to study the activatable PDT effect in vitro on 4T1 cells. By confocal microscopy with DCHF-DA in the presence of H_2_O_2_ the photosensitization capability of F127-TPETCF@MIL-100 was demonstrated. After light excitation (white light excitation, 50 mW∙cm^−2^, 5 min) in the presence of H_2_O_2_ cell death was observed, which was not the case without H_2_O_2_. If TPETCF could induce nonspecifically apoptosis in both normal and tumour cells, F127-TPETCF@MIL-100 specifically induced cell death in the tumour. In vivo experiments in subcutaneous 4T1 tumour-bearing mice both TPETCF and F127-TPETCF@MIL-100 injected into the tumour could inhibit tumour growth after light illumination (50 mW∙cm^−2^, 10 min) but the tumour treated with F127-TPETCF@MIL-100 had a slower growing rate. The hydroxyprobe-1 kit was used to show that the hypoxia was weaker in the tumour treated with F127-TPETCF@MIL-100. After intravenous injection, F127-TPETCF@MIL-100 localized mainly in liver and tumour and could still inhibit the tumour growth.

The same year, Lan et al. [123] proposed also a nanoscale MOF elaborated from Fe_3_O clusters and 5,10,15,20-tetra(p-benzoato)porphyrin (TBP). The Fe-TBP had a length of 100 nm and was able to catalyze the decomposition of H_2_O_2_ to produce O_2_. Incubation in CT26 cells under low O_2_ conditions led to the accumulation of HIF-1α, but when heated with Fe-TBP, the signal decreased significantly showing the decrease of hypoxia thanks to the production of O_2_. After irradiation (650 nm, 20 mW∙cm^−2^, 15 min) under hypoxic conditions and using SOSG in solution, they proved that after addition of H_2_O_2_ Fe-TBP was able to produce ^1^O_2_ similar than the production in normoxic conditions whereas the control H_4_TBP or Hf-TBP without Fe(III) generated only traces of ^1^O_2_. IC_50_ in normoxic conditions were 2.60±1.59, 11.33±6.75, 25.13±6.83 mM for Fe-TBP, Hf-TBP and H_4_TBP. In hypoxic conditions, Fe-TBP presented an IC_50_ of 3.10±1.66 whereas H_4_TBP or Hf-TBP were totally ineffective (IC_50_ superior to 50 mM). In vivo experiments were performed in mouse model of CT26 colorectal adenocarcinoma (650 nm, 100 mW∙cm^−2^, 7.5 min) and PDT was effective in both normoxic and hypoxic conditions. Interestingly, they studied the effect of PDT in immunotherapy. A bilateral model of CT26 tumours was built.

One of the tumours was treated with Fe-TBP and illuminated and the other tumour did not receive any treatment. Then an α-PD-L1 treatment was performed. Both tumours decreased (more than 90%) with a very low dose of Fe-TBP (0.2 mM and 45 J∙cm^−2^). They also demonstrated that PDT together with α-PD-L1 treatment induced formation of cytotoxic T cells that could infiltrate in distant tumour.

A very sophisticated system has been developed by Liu et al. [124]. They designed black phosphorus nanosheeta (BPNSa) on which they coupled FA by non-covalent interaction to target FA receptor as well as a DNA duplex made of Cy5-aptamer-heme (H1) and 3’-heme-labelled oligo-nucleotide (H2) (Cy5-dHeme-BPNS-FA). The idea was that after endocytosis the strong affinity between intracellular ATP and the aptamer would induce the release of H1 and H2 that would be able to concert H_2_O_2_ to O_2_. In solution in presence of Cy5-dHeme-BPNS-FA and ATP, the fluorescence of Cy5 increased proving the release of Cy5 labelled H1. When H_2_O_2_ was added, the formation of O_2_ was detected. Using DPBF, Cy5-dHeme-BPNS-FA produced ^1^O_2_ mainly due to BPNS. In normal air atmosphere, ^1^O_2_ formation of Cy5-dHeme-BPNS-Fa in presence of ATP was 44.6 % higher than without ATP. Under hypoxic conditions, after 20 min irradiation (660 nm, 150 mW∙cm^−2^) in vitro, ^1^O_2_ formation was even 16.0- and 6.8-fold higher with and without ATP. In HeLa cells, [Ru(dpp)_3_]Cl_2_ was used to show that activated heme was essential in O_2_ formation in hydroxic and normoxic HeLa cells. Thanks to SOSG, it was also possible to demonstrate in vitro production of ^1^O_2_. Cell death after PDT (660 nm, 150 mW∙cm^−2^, 3 min) in normoxic conditions treated with Cy5-BPNS-FA was 35.8 % whereas it was 79.1 % for cells treated with Cy5-dHeme-BPNS-FA due to higher concentration of O_2_ after decomposition of H_2_O_2_. In vivo in subcutaneous 4T1 tumour-bearing female nude mice (660 nm, 150 mW∙cm^−2^, 10 min), the tumour volume decreased to 6% for the group treatment with Cy5-BPNS-FA and 45% for the group treatment with Cy5-dHeme-BPNS-FA in good agreement with in vitro experiments (Table 7).

#### 3.1.3. Platinum (Pt) NPs

Pt NPs are other types of artificial catalases. They can also be used as catalysts and are well-known to possess good peroxidase-like properties. They can indeed catalyze in situ the intracellular H_2_O_2_ to produce large amounts of O_2_ thanks to the decomposition of H_2_O_2_. In the literature, we found five publications describing the use of Pt NPs all in 2018 and four of them reported in vivo experiments. In all of them, Pt NPs are associated with others NPs. Pt NP converts H_2_O_2_ into O_2_.

Zhang et al. [125] developed Pt NP (approximately 2 nm) in a metal-organic framework (average diameter of about 90 nm) coated with PEG. The dynamic diameter of the NP was 160.1 nm. They chose the TCPP as the PS. Thanks to dichlorodihydrofluorescein diacetate (DCFH-DA) and DPBF they proved that the NP could efficiently produce ^1^O_2_ in hypoxic conditions in the presence of H_2_O_2_. After intravenous injection in hepatoma 22 (H22) tumour-bearing mice after light illumination (638 nm, 1 W∙cm^−2^, 10 min), the tumour growth of the group treated with the NP and light was significantly different from the groups with only light and only NP (around six times less).

Wei et al. [126] decided to develop 2D Pd-based nanoplates as a carrier. They coupled SH-PEG-COOH to improve the stability and coupled Ce6 onto the carboxylic acid group (10 wt%). The resulting Pd@Pt-PEG-Ce6 nanoplates have a hydrodynamic size of about 80 nm. Using DPBF, they proved that Pd@Pt-PEG-Ce6 and Ce6 produced the same amount of ^1^O_2_. Adding H_2_O_2_, only Pd@Pt-PEG-Ce6 was able to increase the production of ^1^O_2_. In vitro experiments in A4T1 cell lines, in N_2_ atmosphere addition of exogenous H_2_O_2_ under 660 nm laser irradiation (5 min, 150 mW∙cm^−2^) produced a diminution of PDT effect with Ce6 but not with Pd@Pt-PEG-Ce6 compared that in air atmosphere, proving the production of O_2_. Under 808 nm irradiation (0.5 W∙cm^−2^, 5 min), Pd@Pt-PEG-Ce6 exhibited a moderate photothermal effect, which could lead to an enhancement of PDT treatment. In vivo studies in Balb/v mice with 4T1 tumour showed that Pd@Pt-PEG-Ce6 under both 660 nm and 808 nm illumination led to the best tumour growth inhibition compared to the others groups (Ce6 alone, light alone, only Pd@Pt-PEG-Ce6).

Wang et al. [127] synthesized hybrid core-shell NPs composed of a polydopamine (pda) core, Pt NP interlayer, zirconium-TCPP (PCN) porphyrin shell and a coordination between Zr_6_ and the carboxyl group of folic acid. The size of Pda-Pt@PCN was 250 nm with a thickness of the shell of about 30 nm. With ADPA and Pda-Pt@PCN, in solution saturated with N_2_, formation of ^1^O_2_ was higher after addition of H_2_O_2_ revealing the formation of O_2_ via the decomposition of H_2_O_2_. The incorporation of Pda-Pt@PCN-FA into FAR over-expressed CT26 cells was higher than that into FAR-negative COS7 cell line demonstrating the FA targeting effect. PDT efficacy was investigated under both normoxia and hypoxia conditions. In normoxic conditions, no difference could be observed between Pda-Pt@PCN-FA and Pda@PCN-FA whereas in hypoxia conditions, Pda@PCN-FA presented higher phototoxicity (nearly 80%). Using a wound-healing test, they also proved that cells treated with Pda-Pt@PCN-FA were less capable of moving compared with cells without treatment demonstrating the advantage of these NPs for the metastatic process. In a CT26-cell-bearing mice modeltumour hypoxia had been improved (hypoxia-inducible factors staining assay), 660 nm illumination (220 mW∙cm^−2^, 5 min) induced a significant tumour suppression effect as well as an inhibition of tumour metastasis.

Cai et al. [128] designed 3D-dendritic mesoporous silica NP (3D-dentritic MSNs DMSNs) in which were incorporated Pt NPs. They coupled NH_2_-PEG-NH_2_ to improve the biocompatibility, triphenylphosphine (TPP) to target mitochondria and loaded Ce6 as a PS (13% (*w*/*w*)). Pt-DMSNs- TPP/Ce6 had an average diameter of around 113.5 nm. An important increase of ^1^O_2_ production by Pt-DMSNs-TPP/Ce6 was noticed with addition of H_2_O_2_ using ADBA_._ A549 cells treated with free Ce6, DMSNs/Ce6, Pt-DMSNs/Ce6 and Pt-DMSNs-TPP/Ce6. As expecting, mitochondria-triggered apoptosis and therapeutic PDT (660 nm, 50 mW∙cm^−2^, 5 min) in vivo efficacy was higher in the cells treated with Pt-DMSNs-TPP/Ce6 than the others conditions.

Ouyang et al. [129] used 2D semiconductor NPs namely BPNS with an average diameter of 200 nm on which Pt NP of 4.2 nm were uniformly placed. BP nanosheet did not induce decomposition of H_2_O_2_ whereas Pt@BP nanohydrids did. Formation of ^1^O_2_ was observed thanks to DPBF whose absorption decreased less than 10% under NIR illumination for Pt@BP but by 35% after adding H_2_O_2_. In vitro antitumour studies were performed on 4T1 cells; after incorporation and light illumination (660 nm, 1 W∙cm^−2^, 10 min), the cell viability decreased of 26% with BP and 65 % with Pt@BP, with a dysfunction of mitochondria. In vivo in mice bearing subcutaneous 4T1 tumours, PDT treatment led to total regression of the tumour (Table 8).

#### 3.1.4. Others

In order to produce O_2_ inside the hypoxic zones of the tumours, three others strategies have been developed. Three studies focused on the decomposition of H_2_O_2_ but not endogenous H_2_O_2_ of tumour cells. In these strategies, the authors encapsulated H_2_O_2_ in nanostructures.

Li et al. [130] designed light-triggered polymeric vesicle coupled to Ce6 and encapsulating H_2_O_2_. Briefly, PAMAM dendrimer coupled to Ce6/cypate and H_2_O_2_ were co-assembled with a block copolymer that could be cleaved upon ROS production into polymeric vesicles (HC@P1-vesicle) (155.2±21.5 nm). Upon 808 nm excitation, H_2_O_2_ was decomposed in O_2_. Upon 660 nm illumination, excitation of Ce6 induced the formation of ^1^O_2_ that disrupted the copolymer and destabilized the vesicle leading to the release of Ce6/cypate into the tumour. One cycle of 660/805 nm illumination induced the fast release of H_2_O_2_ due to cleavage of the copolymer and disruption of the vesicle. 808 nm excitation of HC@P1-vesicle did not lead to high formation of ^1^O_2_ but 660 nm excitation did. One cycle of 660/805 nm illumination induced an increased amount of ^1^O_2_. In human pancreatic BxPC-3 cancer cells, the cellular uptake of CC-PAPAM was five times higher with light irradiation of HC@P1-vesicle than without light. A higher PDT effect (805 nm, 1000 mW∙cm^−2^, 3 min - 660 nm, 100 mW∙cm^−2^, 10 min) was observed for HC@P1-vesicle than for the same vesicles without H_2_O_2_. In vivo experiments in BxPC-3 tumour-bearing mice (805 nm, 1000 mW∙cm^−2^, 3 min - 660 nm, 100 mW∙cm^−2^, 10 min) showed that HC@P1-vesicle preferably accumulated into the tumour compared to CC-PAMAM. After one cycle of 660/805 nm illumination, CC-PAPAM released from the vesicle deeply distributed into the tumour. 

Thanks to pimonidazole, after treatment with HC@P1-vesicle and one cycle of 660/805 nm illumination it was proved that the hypoxia decreased in the tumour. Multiple cycles of 660/805 nm illumination were then performed. After five cycles, in vivo, tumour treated with HC@P1-vesicle had less than 20% hypoxic areas compared to more than 70% for tumour treated with only 660 nm light. A total ablation of hypoxic hypopermeable pancreatic tumour was observed.

**Figure 22:**
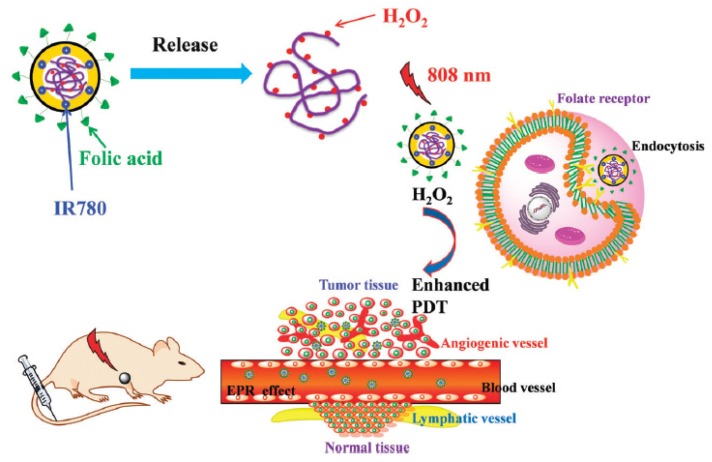
Illustrations of O_2_ self-enriched PLGA–FA/IR780–H_2_O_2_ NPs and their application for PTT and enhanced PDT against tumours. Republished from [131] with permission of the Royal Society of Chemistry, Copyright 2018.

Wang et al. [131] developed NPs (PLGA–FA/IR780–H_2_O_2_ NPs) composed of a core-shell (poly(lactic-co-glycolic acid) (PLGA) and H_2_O_2_/poly(vinylpyrrolidone) complex (O_2_ source)) convalently conjugating folic acid (targeting) and entrapping in the shell IR780 diodide (PTT/PDT agent) (Figure 22). The authors proved the photothermal effect of these NPs as well as their ability to produce ROS to kill cancer cells in vitro in HepG2 cell line after laser irradiation (808 nm, 0.5 W∙cm^−2^, 60 s) with a decrease of cell viability of 80%. The phothermal effect conducted to an efficient release of H_2_O_2_ to increase the O_2_ concentration. These observations were confirmed in vivo in Hep-G2 tumour-bearing mice with a significant inhibition of tumour growth.

In 2011, Manifold et al. [132] described a preliminary study on the influence of topical application of H_2_O_2_ cream on the potential photodynamic reaction (PDR) by 5-ALA methyl ester (MAL) on skin diseases. In this study, the aim was to investigate the possible side effects of H_2_O_2_ cream (Crystacide^®^), of MAL cream under different pO_2_. Forty healthy volunteers participated in the protocol and each of them wore a patch on the right forearm. The patch was constituted of four sites: i.e. (a) normal skin without any treatment, (b) a positive control with MAL cream and further illumination after removal of excess of PS, (c) same as (b) in hypoxic condition, and (d) same of (b) with H_2_O_2_ cream. Another patch on the left forearm was devoted to control the different factors taken alone (light, MAL cream, Crystacide^®^). An evaluation by the naked eye was carried out to highlight the presence of erythema or oedema on the cutaneous sites. Laser Doppler perfusion imaging quantified the change in cutaneous blood flow in vivo. The first results indicated a great variability in terms of appearance of erythema as a function of time and 25% of volunteers showed strong response. Regarding oedema, this side effect was much less problematic. In summary, further investigation should be necessary before topical administration of Crystacide^®^ for cutaneous PDR applications.

Two others studies have described two other systems to decompose endogenous H_2_O_2_. Zhen et al. synthesized a BiOI/BiOIO_3_ nanocomposite (BB NCs) (108 nm) [133]. The photogenerated electrons (e^−^) under 650 nm light irradiation reacted with H_2_O_2_ to produce ^●^OH and slit H_2_O to produce O_2_ then ^1^O_2_ both in hypoxic and normoxic conditions. ^●^OH was detected thanks to hydroxyphenyl fluorescein probe (HPF) after 650 nm illumination and its concentration was even higher after addition of H_2_O_2_. ^1^O_2_ was detected thanks to SOSG probe even in hypoxic conditions. In HeLa cells, the use of SOSG probe also proved the formation of ^1^O_2_ even in hypoxic cells. DCHF-DA presented the same fluorescence in normoxic or hypoxic cells showing the equal amount of ROS production. Fluorescence of ([Ru(dpp)_3_]Cl_2_ was quenched even in hypoxic conditions in the presence of irradiated BB NCs. In vivo (650 nm, 500 mW∙cm^−2^, 15 min), the tumour in the BB NCs/laser group was totally destroyed and a low level of HIF-1α was detected.

**Figure 23:**
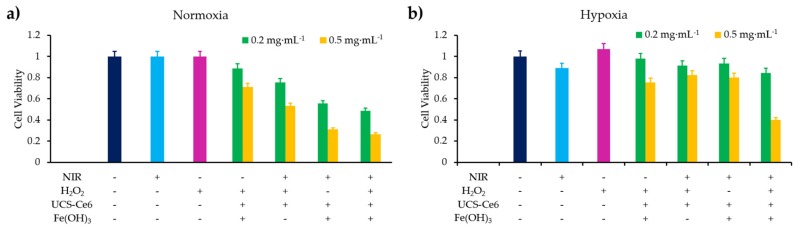
(**a**,**b**) CCK-8 assay of 4T1 cells after incubating in a normoxia/hypoxia environment and treated by different methods. Adapted from Wu et al. [134]

Wu et al. [134] synthesized an UCNP containing Ce6 and Ferric Hydroxide Fe(OH)_3_, biocompatible and acting as a synergetic agent between PDT and chemotherapy. When irradiated at 808 nm in presence of H_2_O_2_, these UCNPs (176 nm diameter) excited Ce6 which generated ^1^O_2_, using O_2_ produced by the Fenton-like reaction of Fe(OH)_3_ with H_2_O_2_. In vitro, 4T1 and 293T human embryonic kidney cells viability was tested in normoxia or hypoxia, in presence or in absence of H_2_O_2_ with or without irradiation (808 nm, 1 W∙cm^−2^, 10 min). They clearly showed the amplified effect of using these UCNPs in presence of H_2_O_2_ and irradiation (Figure 23).

In vivo, the 4T1 mouse tumour model was irradiated at 808 nm (1 W∙cm^−2^, 30 min) and 14 days after treatment with UCPs, not only the growth of tumour dramatically decreased, but also the tumour nearly disappeared (Table 9). 

### 3.2. Water Splitting

Until 2010, water splitting was essentially studied for H_2_ production as it appears a potentiate solution to the energy and environmental issues. Numerous active photocatalysts such as metaloxides, sulfides, nitrides, nanocomposites, and doped materials have been proposed for the generation of H_2_ (and concomitant O_2_ generation) [135]. However, it is well known that nature (and especially plants) is able to use light to catalyse the synthesis of O_2_ from H_2_O in chloroplasts with high yields. Inspired by this and, as water is the most abundant element in the human body, scientists have thought of using light-driven water splitting for in situ O_2_ formation at the tumour site and thus decrease hypoxia. For this, some teams have developed various O_2_ generators based on oxides (CaO, ZnO, Bi_2_WO_6_ …), carbon nitride NPs and other materials such as nanorods or nanosheets.

CaO_2_ is an O_2_-generating material that can be used to suppress hypoxia. It has many advantages such as highly compatible, efficient O_2_ generator and rapidly metabolized in cells. When it reacts with water or weak acid, it can release O_2_ and can alleviate tumour hypoxia:

2CaO_2_ + 4H_2_O → 2Ca(OH)_2_ + 2 H_2_O_2_ → 2Ca(OH)_2_ + 2H_2_O + O_2_ (1)

Hu et al. [136] developed an « optical battery » based on UCNP associated with RB by electrostatic interactions in biocompatible PDMS (polydimethylsiloxane) which was able to produce ^1^O_2_ after NIR irradiation (980 nm, 2 W∙cm^−2^, 5 s). The persistent luminescence of the battery allowed to irradiate during a short time (5 s) and to induce a PDT effect without continuous irradiation. This avoided photothermal effects. 

**Figure 24:**
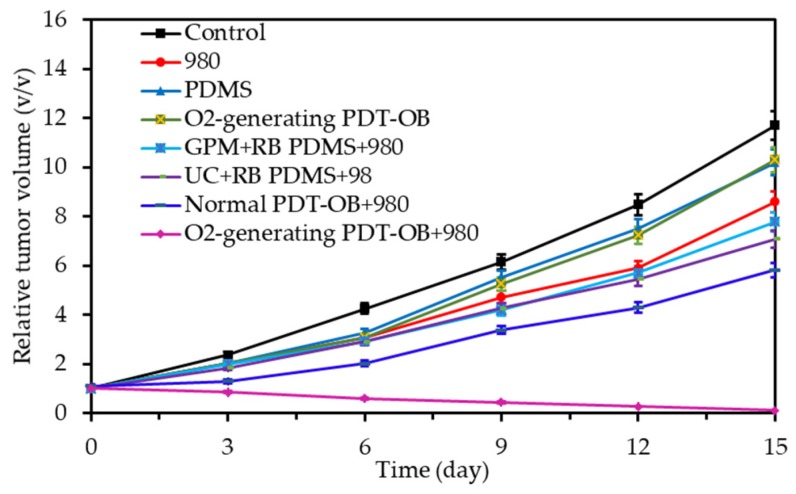
Tumour volumes evolution in different treatments groups. PDT-OB = O_2_ generating optical battery; GPM: Green luminescent material Adapted from Hu et al. [136].

The battery was implemented under different thicknesses of pork tissue. The NIR irradiation (980 nm) induced ^1^O_2_ production at a thickness of 4 mm, whereas UV light (365 nm) was efficient at only 1 mm. The battery was biocompatible in a human colon adenocarcinoma cell line HT29 and presented phototoxicity under light illumination. CaO_2_ was encapsulated in the battery and a higher molecular O_2_ concentration and hypoxia inhibition was observed. Finally, the device was implanted subcutaneously onto a HT29 solid tumour. The PDT effect of the battery after NIR irradiation (660 nm, 30 mW∙cm^−2^, 30 + 60 s) during 15 days (two times each day) led to a better oxygenation and a tumour volume decrease (Figure 24). 

**Figure 25:**
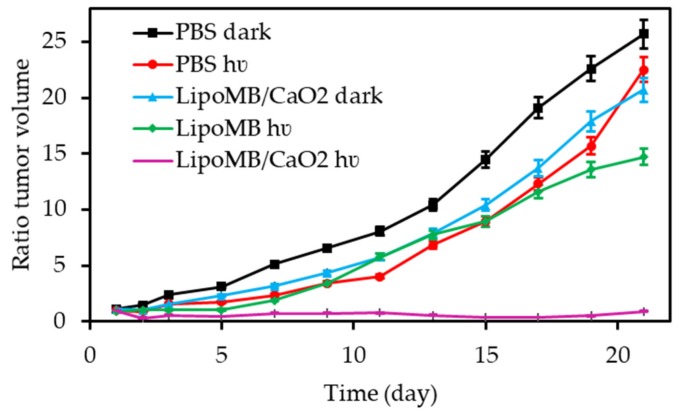
Relative tumour volume after different treatments. Adapted from Liu et al. [137].

A liposome-based NP containing MB and the O_2_ supplier CaO_2_ were synthetized by Liu et al. [137]. After a short irradiation time (30 s), photobleaching of the NPs occurred inducing the formation of O_2_ by release of CaO_2_ in water. After a long irradiation time (60 s), hypoxia in the tumour environment was regulated and a PDT effect was observed. The NPs were tested on 4T1 cells. The regulation of hypoxia was demonstrated using a ROS detection kit. Dual-stage irradiation (660 nm, 30 mW∙cm^−2^, 1 min and 5 min) led to improved phototoxicity compared to single irradiation. The NPs were tested on subcutaneously 4T1 solid tumour bearing mouse. Tumour inhibition was observed after light illumination (658 nm, 280 mW∙cm^−2^, 2+8 min) whereas negligible effect appeared in the dark group (Figure 25).

Sheng et al. [138] synthesized a core shell NP with a CaO_2_ core coated with a pH sensitive methacrylate based copolymer that could undergo a dissolution at pH lower than 7.4, leading to the exposure of the CaCO_2_ to aqueous medium. The authors prepared a de-oxygenated PBS solution containing SOSG and RB and added CaO_2_ NPs and irradiated (white light, 5 min). The results revealed a significant increase (324.8%, *p* < 0.001) of SOSG fluorescence compared to the control experiments, indicating the ability of the NPs to provide O_2_ during PDT, to enhance the formation of ^1^O_2_. The injection of NP in a BxPC-3 human pancreatic cancer cell line demonstrated an enhancement of the PDT effect. In vivo experiments were performed in Mia-PaCa 2 xenograft mice, (3*3 min Fenix LD01 LED50 mW, 205 J∙cm^−2^) which is a model known to form hypoxic tumours. No significant difference in tumour volume for mice treated with PDT alone or CaO_2_ NPs alone 5 days after treatment was observed whereas a significant reduction of 70.5% was observed for animals treated with the CaO_2_ NPs and PDT.

C_3_N_4_ is known as a water splitting material biocompatible but presents limit efficiency in the red light region. Carbon-dot (CD)-doped carbon nitride (C_3_N_4_) NPs were designed by Zheng et al. [139] to enhance red light absorption and allow O_2_ production via water splitting. To overcome the limit efficiency in red light region, carbon dots have been added. PpIX-PEG-RGD was grafted on the NP. The association showed an enhanced O_2_ production compared to C_3_N_4_ alone, allowing the decrease of hypoxia. ROS production after light illumination (630 nm, 80 mW∙cm^−2^, 10 min) was measured in a 4T1 cell line. An enhancement could be observed with the NP, and not for PpIX or C_3_N_4_ without CDs. After laser irradiation at 630 nm, NP displayed the same phototoxicity in hypoxic and normoxic medium, contrary to PpIX. The NPs were tested in subcutaneously 4T1 solid tumour bearing mouse. NP injection and irradiation showed remarkable tumour growth inhibition contrary to PpIX or C_3_N_4_ without CDs.

Li et al. [140] associated Fe-C_3_N_4_ NPs with tris(bipyridyl)cationic Ru complexes (Ru(bpy)_3_^2+^) which enhance photocatalytic activity and act as PS. Ru complex absorption of near infrared light enhanced the charge transfer to C_3_N_4_ and photocatalytic water splitting. The NPs irradiated at 800 nm (2.7 W, 5 min) allowed more O_2_ generation than C_3_N_4_ or (Ru(bpy)_3_^2+^) alone. The ROS production was also confirmed by using DCFH-DA and ESR. The NP biocompatibility, cellular uptake and phototoxicity (800 nm, 2.7 W, 3 min) were demonstrated on a 4T1 cell line. Experiments on 4T1 tumour bearing mice after intravenous injection of NPs and light irradiation (800 nm, 2.7 W, 5 min) showed significant tumour inhibition. 

**Figure 26:**
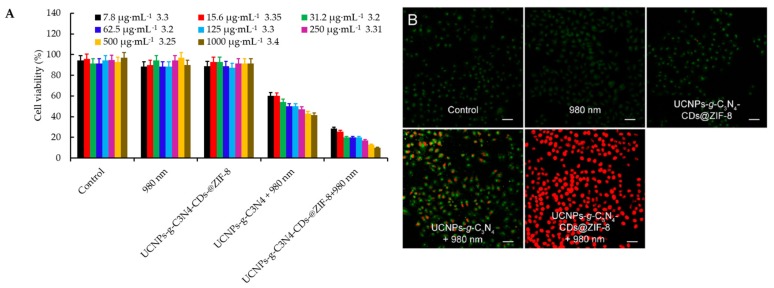
(**A**) In vitro cell viabilities of HeLa cells incubated with cell culture (control), 980 nm light, UCNPs-g-C3N4 with 980 nm laser irradiation, and UCNPs-g-C3N4−CDs@ZIF-8 at varied concentrations with and without 980 nm laser irradiation. Adapted from Yang et al. [141] (**B**) CLSM images of HeLa cells incubated with different conditions corresponding to the toxicity test in vitro, and all the cells are marked with calcein AM and PI. Reprinted from [141] with permission from the American Chemical Society, Copyright 2017.

Yang et al. [141] developed a theranostic platform that associated UCNPs (NaGdF_4_:Yb,Tm@NaGdF_4_) graphic-C_3_N_4_ nanosheets (one PS) and carbon dots (another PS) incorporated in a ZIF-8 metal−organic framework (MOF) shell. The combination of g-C_3_N_4_ and CDs could induce stepwise water splitting: (1) _2_H_2_O → H_2_O_2_+H_2_; (2) 2H_2_O_2_ → 2H_2_O + O_2_. The phototoxicity was observed on HeLa cells after NIR irradiation (980 nm, 500 mW∙cm-^2^, 5 min) (Figure 26). The NPs injected in U14 tumour-bearing mice irradiated at 980 nm (0.5 W∙cm^−2^, 10 min) induced a delayed tumour growth. 

Zheng et al. [142] designed a platform assembling an O_2_-generated thylakoid membrane from plants (spinach, lettuce, carbage) fused with synthetic NP (Ag, SiO_2_, ZnO). This idea come from the fact that in nature, plants possess a very oxygenic photosynthesis system that allow the photocatalyzed formation of O_2_. These platforms exhibited good O_2_ generation that could reverse the tumour hypoxia. This O_2_ production proved to be higher compared to CaO_2_ or MnO_2_. The in vivo generation of O_2_ was proven in CT26 tumour bearing Balb/C mice. The authors observed enhanced PDT when they added the platform to MB (660 nm, 155 mW∙cm^−2^, 2 min). 

**Figure 27:**
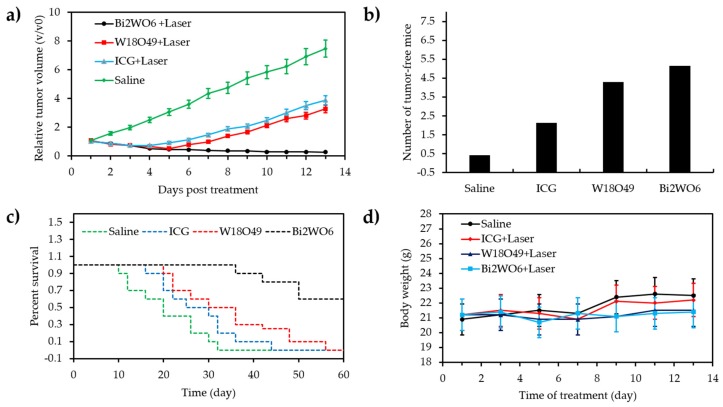
(**a**) Tumour volumes of HeLa-tumour-bearing mice that received different treatments as displayed. (**b**) Number of tumour-free mice after treatment during the observation. (**c**) Survival curves of HeLa-tumour bearing mice that received different treatments as displayed. (**d**) Body weight of HeLa-tumour-bearing mice that received different treatments as indicated. Adapted from Zhang et al. [144].

In 2015, Zhou et al. [143] synthesized of a monolayer of Bi_2_WO_6_ with highly active surface that could generate photocatalytic oxidation reactions, leading to the oxidation of H_2_O (or OH^−^ ) to ^•^OH upon laser irradiation. Indeed, Bi_2_WO_6_ could produce ROS without the need of O_2_. Zhang et al. [144] demonstrated that small Bi_2_WO_6_ NPs (5 nm) grafted with carboxylic acid groups could efficiently generate ^●^OH after irradiation in the NIR grange (808 nm, 1 W∙cm^−2^, 5 to 20 min). The in vivo injection of the NP in HeLa tumour bearing nude mice showed a photothermal effect until 47 °C after 8 min irradiation (808 nm, 1 W∙cm^−2^) because of their strong Plasmon resonance. The ROS production was evaluated both by the SOSG for ^1^O_2_ and by EPR for ^●^OH radicals. Formation of ^●^OH played a dominant role. The in vivo PDT (HeLa tumour bearing mouse model, 808 nm, 1 W∙cm^−2^, 8 min) showed that Bi_2_WO_6_ NPs induced photodynamic killing in a hypoxia-free manner resulting in tumour continuous decrease of the tumour volume (Figure 27). It was not the case for ICG and W_18_O_49_ NPs, which could react, with O_2_ absorbed onto the NP to generate ROS in an O_2_ independent manner.

**Figure 28:**
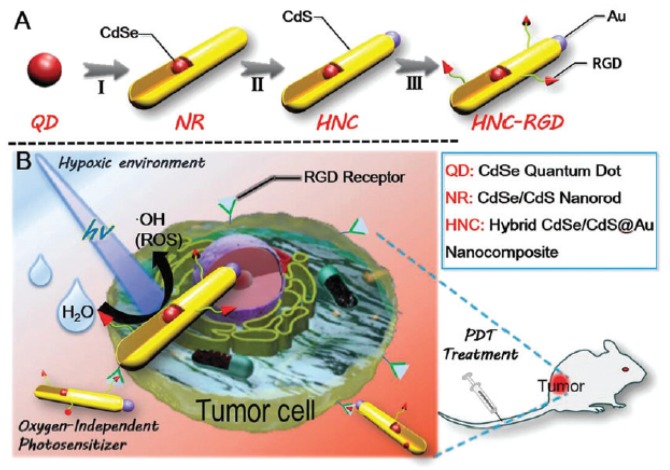
(**A**) Structure of HNCs: (I) anisotropic growth of NRs; (II) gold deposition on top of NRs; (III) RGD modification of HNCs; (**B**) schematic diagram of visible light driven water splitting to generate ROS for PDT treatment. Republished from [145] with permission from the Royal Society of Chemistry, Copyright 2017.

Fan et al. in 2017 [145] developed semiconductor materials with broadband light response in UV and visible region. Usually photocatalytic reaction produce O_2_ by splitting water, in vivo, but not enough to produce ROS in sufficient quantity by PDT. To improve the efficiency, such nanorods (CdSe-seeded/Cds) were doped with deposited Au, forming a hybrid nanocomposite (HNC) (Figure 28). RGD peptide was coupled to target α_v_β_3_ receptors. Under visible excitation, the nanorods induced charge separation and delocalized electrons, which were transferred to Au, increasing the ROS generation by water splitting.

In vitro, excitation was performed with a blue LED (450 nm, 500 mW∙cm^−2^, 10 min). A significant increase of intracellular ROS was observed in COS-7 and HeLa cells with HNC in normoxic conditions. Moreover, cytotoxicity assays have been performed on 4T1 cells, with irradiation by blue LED during 1, 2 and 3 min. 20% viability of cells was observed in normoxic and hypoxic conditions, to be compared to the 80% obtained with non-modified nanorods (CdSe-seeded/Cds). In vivo model was the murine 4T1 breast-adenocarcinoma mouse on female balb/c mice. Obvious tumour growth inhibition was observed when NCNs-RGD were used under blue light irradiation (30 min), indicating the excellent accumulation of these nanorods in the tumour and improvement of the PDT effect in hypoxic media (Table 10).

### 3.3. Destruction of Tumour Extracellular Matrix (ECM)

Proteins such as collagen, glycoproteins, elastin, and proteoglycans are constituents of normal connective tissues. The tumour-associated proteases are likely to destroy some of these proteins inducing, by the way, the invasive process and thus metastasis. However, destruction of the extracellular matrix may be useful if it is cleverly applied. Indeed, it can lead to better vascularization and therefore to an excess supply of O_2_, thus reducing hypoxia.

In 2016, Gong et al. [146] investigated the effect of the administration of hyaluronidase (HAase) before PDT with nanomicelles containing Ce6 (NM-Ce6). The HAase treatment could degrade ECM by breaking down hyaluronan leading to an increase of the blood vessel density and the perfusion of the tumour. The accumulation of NM-Ce6, with an average size of 33 nm, in tumours was studied after administration of different doses of HAase (0, 375, 750, 1500 and 3000 U) and a significant increase in the tumour uptake of NM-Ce6 (~ 2-fold) was observed with the HAase treatment with an optimal dose at 1500 U. The significant decrease of HIF-1 α after this HAase administration proved an improvement of hypoxia conditions in the tumour. In vivo PDT studies were performed in balb/c mice bearing 4T1 tumours. No effect on the tumour growth was observed with the administration of HAase alone but in combination with PDT treatment with NM-Ce6 under light irradiation at 660 nm (2 mW∙cm^−2^, 1 h), there was an almost total inhibition of the tumour growth. The authors also investigated the effect of HAase administration on PDT efficiency against lymphatic metastasis since HAase presented the ability to move in drainage lymph nodes and thus increased the EPR effect of NM-Ce6 and relieved the hypoxia in both the tumour and the metastases sites. To reduce invasiveness of local injection of HAase, this last one was PEGylated to form HAase-PEG for systemic local injection and the same EPR and hypoxia effects were observed.

**Figure 29:**
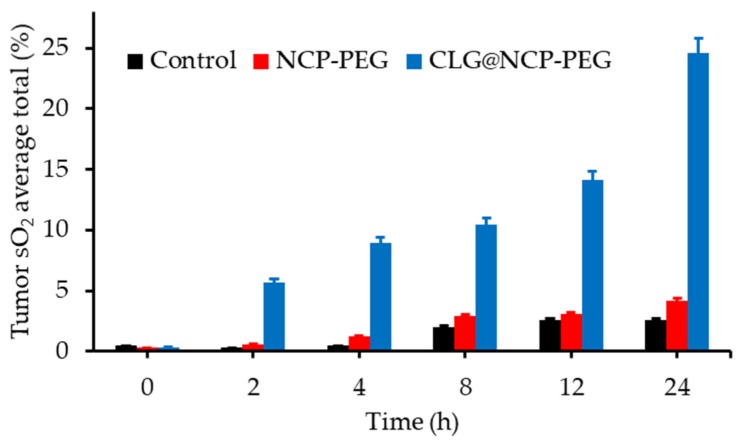
Quantification of the oxyhemoglobin saturation in the tumour from different groups over time. Adapted from Liu et al. [147].

Liu et al. [147] described another strategy, based on two steps to enhance PDT treatment: a first injection of NPs containing collagenase (CLG) to destroy tumour ECM by degradation of collagen and a second injection of liposomes containing Ce6 for PDT treatment. The pH-sensitive NPs were composed of Mn^2+^ coordinated with benzoic-imine (BI)-linker to form nanoscale coordination polymers (NCPs) encapsulating CLG (CLG@NCP). CLG@NCP NPs were surrounded by a lipid PEG shell to finally formed CLG@NCP-PEG NPs possessing a hydrodynamic size of 106 nm. The pH-sensitive relapse of CLG by CLG@NCP-PEG NPs was investigated by changing the pH of a solution containing CLG@NCP-PEG NPs, from 7.4 to 6.5 and after 24 h of incubation a release of ~90% was observed. Moreover, a recovery of ~80% of CLG enzyme activity was highlighted for a pH 6.5. In vivo in 4T1 tumour bearing mice, a high tumour uptake was observed 24 h after injection of CLG@NCP-PEG NPs as well as a significant decrease of the collagen level in the tumours leading to an ECM degradation and thus an enhancement of blood flow perfusion. As a consequence of these effects of the level of tumour oxygenation, a significant increase over time was observed after treatment with CLG@NCP-PEG NPs (Figure 29) as well as a high decrease of hypoxia signals. 

The effect of a pretreatment with CLG@NCP-PEG NPs before PDT treatment with liposomes containing Ce6 (liposome@Ce6) was finally studied. The therapeutic PDT effect was dramatically improved with an effective tumour growth inhibition when mice where pretreated with CLG@NCP-PEG NPs 24 h before injection of liposome@Ce6 and irradiated (660 nm, 5mW∙cm^−2^, 60 min) after again 24 h. Moreover, CLG@NCP-PEG NPs showed no cytotoxicity over time (14 days) (Table 11).

### 3.4. Decrease of Tumour O_2_ Consumption

A recent strategy consists to decrease the tumour cells’ demand for O_2_ by inhibiting mitochondrial respiration ((*via* NADH dehydrogenase). Several mitochondrial receptors can be targeted such as papaverine’s or metformin’s complexes and others. The expected reduced O_2_ consumption induces an increased oxygenation concentration. Since 2015, some drugs with potential ability to decrease the hypoxic fraction have been developed in the field of PDT and/or PTT. Nevertheless, some requirements must be taken into account to obtain a significant improvement in the treatment of cancer. Normally oxygenated tissues should not be affected, uptake by tumour cells should be rapid, minimal side effects and optimal clearance should be observed. Two teams have reported the use of metformin (Met) which is known to reduce tumour O_2_ consumption and thus increase tumour oxygenation. 

In 2017, Song et al. [148] incorporated in PEG liposomes, HCe6 (hydrophobic Ce6) and a hypoglycemic agent, Met. The resulting NPs exhibited a ^1^O_2_ generation under 660 nm light excitation. In vitro studies were performed in 4T1 cells to evaluate the PDT efficiency of Met-HCe6-liposomes in comparison with HCe6-liposomes and Met or Ce6 alone. After a 660 nm light irradiation for 10 min, all Ce6 formulations showed almost the same PDT effect in normoxic conditions. In vivo tests were performed in mice bearing 4T1 tumours injected with Met-HCe6-liposomes. The authors used PA to evaluate the tumour oxygenation in presence of Met alone or Met-liposomes by following the level of oxygenated Hb (oxyhemoglobin) in tissues. A high increase was observed for both after 2 h with a decrease over time, after 2 h, for Met alone. However, for Met-liposomes, the increase of oxyhemoglobin subsists over time due to a continuous release of Met. After injection of Met-HCe6-liposomes, a significant reduction of hypoxia was observed 2h and 24h after injection (57% to 27% and 15% respectively), by using pimonidazole hydrochloride. The improvement of tumour oxygenation with this compound was also proved in two other tumour models (CT26 colon cancer and SCC-7). Finally, the improvement of PDT efficiency was confirmed by in vivo studies in a 4T1 tumour model. After injection of Met-HCe6-liposomes or HCe6-liposomes 24h before a 660 nm light irradiation (0.035 W∙cm^−2^, 30 min), the tumour growth was highly decreased for both in comparison with PDT treatment with Ce6 alone. Moreover, the most significant inhibition of tumour growth was observed for group treated with Met-HCe6-liposomes-mediated PDT (Table 12).

Zuo et al. [149] designed NPs (PM-W_18_O_49_-Met NPs) composed by W_18_O_49_ NPs (PDT and PTT application) and Met encapsulated in platelet membranes (PM) for a better biocompatibility. They first evaluated the properties of W_18_O_49_ NPs and PM-W_18_O_49_ NPs without Met and found respective mean diameter of 5 nm and 115 nm. Under an 808 nm laser irradiation (1 W∙cm^−2^, 10 min), a solution of PM-W_18_O_49_ NPs showed an increase of temperature from 24.2 to 61.6 °C over time. Moreover, they observed ^1^O_2_ production and of the ability to transform laser energy to heat for PM-W_18_O_49_ NPs contrary to W_18_O_49_ NPs alone indicating a protecting effect of PM. The introduction of Met in PM-W_18_O_49_ NPs did not affect its characteristics (size, morphologic, optical) and PM-W_18_O_49_-Met NPs allowed an effective release of Met in the tumour. The efficiency of PM-W_18_O_49_-Met NPs to reduce tumour O_2_ consumption was evaluated in Raji cells and compared to that of Met alone. For both compounds, a significant decrease (70%) of O_2_ consumption rate was observed after 24h incubation. An induction of hypoxia simultaneously to increase of ROS production was observed for PM-W_18_O_49_ NPs whereas a higher level of ROS production was observed for PM-W_18_O_49_-Met NPs with a decrease of hypoxia. In vitro PTT/PDT studies were performed in Raji cells incubated with PM-W_18_O_49_-Met NPs, PM-W_18_O_49_ NPs or W_18_O_49_ NPs and irradiated at 808 nm (1 W∙cm^−2^, 10 min). A highly decrease of cell viability was observed for all these compounds but PM-W_18_O_49_-Met NPs presented the highest cytotoxicity with a level of viability inferior at 20%. The antitumour efficacy was confirmed by in vivo tests in mice bearing Raji-lymphoma xenografts injected with the same compounds and irradiated at 808 nm (10 min). 

Two others systems based on two different compounds (tamoxifen, TAM, and atovaquone, Ato) able to reduce O_2_ consumption have been described. In 2018, Yang et al. [150] developed a system based on the use of TAM. They combined in solution Ce6-modified HSA (HSA-Ce6) with TAM to induce a self-assembly of NPs which could be dissociated under acidic pH. They also synthesized as a control covalently cross-linked HSA-Ce6 NPs (C-HSA-Ce6 NPs). The NPs formed: HSA-Ce6/TAM NPs and C-HSA-Ce6 NPs presented a hydrodynamic size of 130 nm and 80 nm respectively. When the environment became acidic, the TAM molecules were protonated, which could induce NPs dissociation. They found that the presence of TAM and HSA did not affect the ^1^O_2_ generation induce by Ce6. In vitro PDT efficiency was evaluated in 4T1 cells incubated with HSA-Ce6/TAM NPs 6 h before 660 nm light irradiation (5 mW∙cm^−2^, 30 min). The HSA-Ce6/TAM NPs showed a photocytotoxicity similar as that observed for C-HSA-Ce6 NPs or Ce6 alone. By performing in vivo studies, in mice bearing 4T1 tumours, the authors observed a tumour uptake of HSA-Ce6/TAM NPs significantly higher than C-HSA-Ce6 NPs or Ce6 alone due to the pH-induced dissociation of HSA-Ce6/TAM NPs in HSA-Ce6 allowing an intratumoural penetration more efficient. Moreover, they investigated the evolution of tumour oxygenation after injection of HSA-Ce6/TAM NPs by using PA imaging and observed an increase of tumour oxygenation from 0.91% to 10.9%. By using pimonidazole they found a reduction of hypoxia from 21.6% to 2.04% for mice treated with HSA-Ce6/TAM NPs. The in vivo PDT efficiency was evaluated by injection in mice of HSA-Ce6/TAM NPs and C-HSA-Ce6 NPs following by 660 nm light irradiation. A high phototoxic effect was highlighted for mice treated HSA-Ce6/TAM NPs and light with some complete eradication of the tumour whereas for the control groups (C-HSA-Ce6 + Light or HSA-Ce6/TAM NPs alone), any inhibition of tumour growth was observed. 

**Figure 30:**
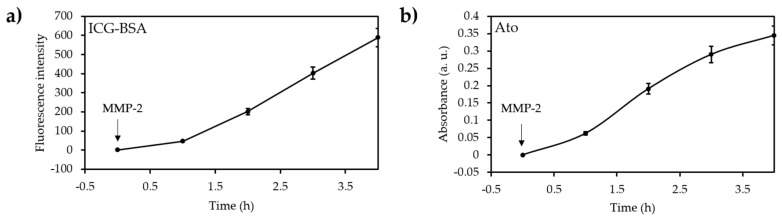
MMP-2-triggered shape remodelling and cell uptake behaviour of Ato-ICG-GNPs. (**a**) Fluorescence intensity of ICG-BSA (795 nm) in the supernatant. (**b**) Absorbance of Ato (490 nm) in the supernatant. Adapted from Xia et al. [151].

Xia et al. [151] described another vehicle with a reducible size based on gelatin for a better tumour uptake encapsulating a PS (ICG-BSA nanocomplex) and Ato. The gelatin NPs thus formed were denoted Ato-ICG-GNPs (437±52 nm). These NPs could be reduced in the tumour by enzymes (matrix metallopeptidase, MMP-2) and thus highly release in the tumour site Ato and ICG-BSA after only 2 h (Figure 30).

**Figure 31:**
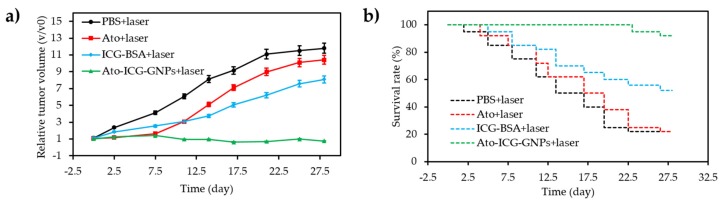
In vivo therapeutic outcome. (**a**) Relative tumour growth curves of mice received different treatments within a total of 28 days. Control (0.1 mL per mouse, PBS); Ato (0.1 mL per mouse, containing 330.15 μg∙mL^−1^ Ato); ICG-BSA (0.1 mL per mouse, containing 37.44 μg∙mL^−1^ ICG); Ato-ICG-GNPs (0.1 mL per mouse, containing 330.15 μg∙mL^−1^ Ato and 37.44 μg∙mL^−1^ ICG). The tumour region was irradiated by a 808 nm laser with a power density of 1 W∙cm^−2^ for a duration of 5 min at 4 h post injection. (n = 18 in each group). (**b**) Survival profiles as represented by the calculated rate of survival. Adapted from Xia et al. [151].

The ability of Ato-ICG-GNPs to reduce tumour O_2_ consumption was investigated in HeLa cells and compare to Ato alone. The O_2_ consumption rate was significantly reduced by 50%, after incubation of cells with 2µM of Ato or Ato-ICG-GNPs and no difference was observed between the two compounds. The evaluation of OCR was done in both normoxic (21% of O_2_) and hypoxic (2% of O_2_) conditions and any influence of O_2_ on Ato efficiency was highlighted. It was also observed that in presence of Ato-ICG-GNPs, the cell proliferation slowed down (Figure 31 and Table 12). 

The PDT efficiency of Ato-ICG-GNPs in comparison with ICG-BSA was investigated in vitro in HeLa cells incubated with Ato-ICG-GNPs or ICG-BSA and irradiated with an 808 nm laser (1.0 W∙cm^−2^, 5 min). A high decrease of cell viability was observed for both with a significantly better phototoxicity for Ato-ICG-GNPs. In vivo studies in mice bearing HeLa-xenograft confirmed the results observed in vitro with a high decrease of tumour growth for mice treated with Ato-ICG-GNPs and laser irradiation (treatment repeated four times) (Figure 31a) and a survival rate of 90% after 3 weeks of treatment (Figure 31b). 

### 3.5. Others

Two others ways to modify the TME have been reported. Gui et al. [152] explored a new strategy consisting in finding a way to deplete ATP in hypoxic cells. They synthesized a metal-organic framework NP with Cu^2+^ and ZnPc-(COOH)_8_ leading to a 3D architecture ((Cu_8_(ZnPc-(COOH)_8_)_n_, ZPCN). ZnPc-(COOH)_8_ was aggregated into the NPs and ROS production was not efficient. The authors showed that addition of ATP led to the formation of ATP-Cu complex and free ZnPc-(COOH)_8_ could then produce again ^1^O_2_. In A549 cells after PDT (665 nm) incubation with Cu^2+^ only did not decrease the survival, whereas with the PS survival was 55.4 % and with ZPCN 23.7 %. They showed also that cells treated with ZPCN caused an important decrease in ATP concentration, arrested in the S phase, suggesting that DNS replication was stopped in the cells, a decrease in GSH level and high production of ROS and induced mitochondrial transmembrane potential depolarization of 68.72%. In vivo in A549 tumour xenografts in nude mice, PDT (665 nm) induced the suppression of tumour growth.

**Figure 32:**
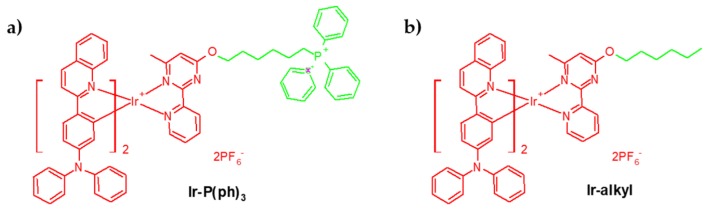
Chemical structures of (**a**) Ir-P(ph)_3_ and (**b**) Ir-alkyl. Adapted from Lv et al. [153].

In 2016, Lv et al. [153] designed two PS based on iridium (III) complexes: Ir(P(ph)_3_ and Ir-alkyl (Figure 32) for the specific targeting of mitochondria and lysosomes, respectively, to enhance the PDT effect. 

The authors treated HeLa cells with both complexes at different O_2_ levels and found that even under hypoxic conditions, the intracellular O_2_ concentration remains high for Ir(P(ph)_3_. They attributed this finding to an inhibition of respiration in mitochondria due to the PS. The PSs exhibited ϕ_Δ_ of 0.17 for Ir(P(ph)_3_ and 0.21 for Ir-alkyl. After incubation with Ir(P(ph)_3_ or Ir-alkyl, under hypoxic conditions, a high intracellular O_2_ concentration was observed with Ir(P(ph)_3_ (11% against 3% for Ir-alkyl). Under a 475 nm irradiation (22 mW∙cm^−2^, 30 min), for both compounds, cell death occurred in 4 h whereas in hypoxic conditions, Ir(P(ph)_3_ was more efficient to kill cells. Moreover, the irradiation of Ir(P(ph)_3_ induced more ROS generation than Ir-alkyl under both normoxic and hypoxic conditions. 

## 4. Combined Therapies

In many diseases, a single treatment is often not effective or selective enough. One solution is to add a second therapy to the usual treatment. As a result, there are many opportunities for clinicians to improve the healing and comfort of patients. The combination of two or more simultaneous or consecutive modes of action may result in an additive or synergistic effects, i.e. creative cooperation. For example, one often speaks of "cocktail effect" in the case of complex mixtures of chemicals. In the field of cancer, various modalities can be applied [154,155] and combining several interventions can minimize drug resistance, or fight against expected resistance.

In this section, recent advancements of PDT associated with various other modalities (chemo-, antiangiogenic-, immuno-, or photothermal therapies) will be addressed.

### 4.1. Chemo-PDT

Chemotherapy is still the major treatment for many cancers. However, cancer cells can develop drug resistance, decreasing the effectiveness of the protocol, or even generating recurrence of cancer. The combination of chemotherapy and PDT as an adjuvant therapy can bring a synergistic effect that can lead to a decrease in drug doses and reduced systemic toxicity.

#### 4.1.1. Tirapazamine (TPZ)

TPZ belongs to the class of benzotiazine-di-N-oxides of hypoxic cytotoxin. Thanks to a one-electron reduction of the molecule, free radical species are formed and induce single and double-strand breaks. Hypoxic microenvironment triggers TPZ. It was the first molecule not based on nitro or quinone functionalities to induce ROS in hypoxic conditions.

Liu et al. [156] designed double silica-shelled UCNP able of co-delivering silicon phthalocyanine dihydroxide (SPCD) and TPZ to afford TPZ-UC/SPCD for the NIR-induced synergetic therapy of tumours, by combining PDT and hypoxia-activated chemotherapy. Under 980 nm laser irradiation (1.4 W∙cm^−2^, 5 min), TPZ-UC/SPCD generated a large amount of ROS, and therefore high PDT efficacy against HeLa cells was achieved, resulting in a severe hypoxia which would further facilitate the activation of TPZ. Furthermore, according to the in vivo studies performed on tumour-bearing nude mice under 980 nm laser light (1.4 W∙cm^−2^, 15 min), TPZ-UC/SPCD showed remarkably suppressed tumour growth compared to UC-PDT alone, which confirmed that the combined TPZ-PDT treatment could lead to marked cell apoptosis, further demonstrating the synergistic effects (Table 13).

**Figure 33:**
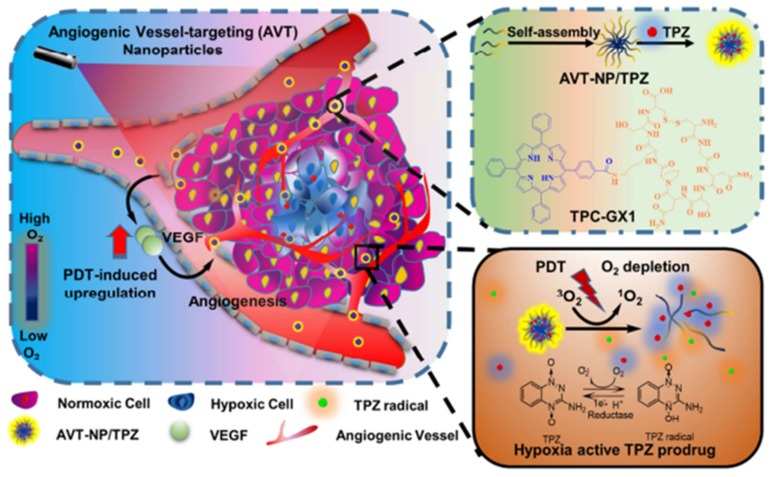
Schematic illustration and characterizations of AVT-NPs. The formation of AVT-NP, generation of cytotoxic TPZ radical under hypoxic conditions in cancer cells, and illustration of AVT-NP/TPZ based PDT that induces a local hypoxic environment and promoted angiogenesis for targeted drug delivery and synergistic chemo-photo therapy. Reprinted from [157] with permission from Elsevier Ltd, Copyright 2017.

Guo et al. [157] designed an angiogenesis vessel-targeting NPs (AVT-NPs) that consisted of a PS (5-(4-carboxyphenyl)-10, 15, 20- tris(3-hydroxyphenyl)chlorin, TPC), angiogenic vessel-targeting peptide (GX1 cyclopeptides), and TPZ (Figure 33). 

The designed AVT-NPs showed high accumulation efficiency at the tumour site, resulting in a large production of ROS under laser (650 nm, 1.2 W∙cm^−2^, 10 min) irradiation, and therefore high PDT efficacy was achieved which resulted in a severe hypoxia and increased angiogenesis. In the meantime, the exaggerated hypoxia further activated the bioreductive prodrug TPZ to also release highly cytotoxic radicals, leading to an enhanced antitumour efficacy both in vitro and in vivo (MCF-7 cells, 650 nm, 1.2 W∙cm^−2^, 10 min) compared to free drug or non-targeted nanodrugs.

Li et al. [158] demonstrated a novel biomimetic nanoplatform (TPZ@PCN@Mem) for tumour- targeted combination therapy. TPZ@PCN@Mem was elaborated by loading the hypoxia-activated prodrug TPZ in a PCN-224 porphyrinic metal organic framework and then coating with the homotypic cancer cell membranes. The authors found that PCN-224 present in the nanoplatform generated large amounts of cytotoxic ROS once exposed to laser irradiation (660 nm, 30 mW∙cm^−2^, 30 s) and the resulting hypoxia in the tumour aggravated by the photochemical O_2_ depletion further facilitated the activation of TPZ for successive bioreductive chemotherapy. Thus, according to the both in vitro (660 nm, 30 mW∙cm^−2^, 5 min) and in vivo (660 nm, 220 mW∙cm^−2^, 10 min) investigations, TPZ@PCN@Mem exhibited highly efficient therapeutic effect against a 4T1 tumour model with negligible side effects. 

Wang et al. [159] reported the development of hybrid PLGA/lipid NPs able of codelivering ICG and TPZ to solid tumours by combining PDT and hypoxia-activated chemotherapy against metastatic breast cancer. Further conjugation of the NPs to iRGD (CRGDKGPDC) peptide provided NPs (iNP/IT) studied in both 3D tumour spheroids in vitro and orthotopic breast tumours in vivo. Upon near-IR laser (808 nm, 2 W∙cm^−2^, 3 min), the NPs showed a high ROS production and therefore an important antitumour efficacy against metastatic 4T1 tumour model under normal O_2_ conditions. Meanwhile, the hypoxic microenvironment in tumours triggered TPZ for synergistic cell-killing effect. Furthermore, according to the in vivo results (4T1 tumour-bearing mice, 808 nm, 2 W∙cm^−2^, 5 min), iNP/IT could inhibit both primary tumour growth and metastasis with minimal side effects contrary to a mixture of NPs containing individual drugs

Chen et al. [160] have successfully developed photolabile HSA-based NPs modified with diazirine (DA) and loaded with ICG and TPZ (ICG/TPZ@HSA dNMs). Such photoresponsive ICG/TPZ@HSA dNMs were able to form aggregates via crosslinking of surface DA groups upon 405 nm (1.0 W∙cm^−2^) laser irradiation, thus causing enhanced tumour site accumulation and prolonged retention time. A successive laser exposure (808 nm, 1.0 W∙cm^−2^, 7 min) of the ICG/TPZ@HSA dNMs at the tumour area enabled to trigger a cascade of synergistic therapeutic events by generation of ROS, hyperthermia, and consequent hypoxia microenvironment, which activated the initially nontoxic TPZ. Following systemic administration to mice bearing 4T1 tumours, ICG/TPZ@HSA dNMs eradicated efficiently the tumours by sequential irradiation of lasers (405 nm, 0.75 W∙cm^−2^, 5 min and 808 nm, 1.0 W∙cm^−2^, 10 min). 

Liu et al. [161] designed a multifunctional (Hf/TCPP) NMOF platform, denoted Hf/TCPP loaded with TPZ. Thanks to their porous surface nature, the Hf/TCPP NMOFs possess a high TPZ loading capacity (80 %). In addition, further surface PEGylation with DOPA-PIMA-mPEG enhanced their dispersibility and stability in physiological media and, significantly controlled the release rate of TPZ within TPZ/Hf/TCPP/PEG. Exposure of TPZ/Hf/TCPP/PEG NMOFs to laser irradiation (635 nm, 12 mW∙cm^−2^, 9 min) provoked an efficient ROS production, triggered the activation of TPZ and as consequently a great cytotoxic effect against both HeLa and 4T1 cells. Finally, the in vivo studies (635 nm, 0.1 W∙cm^−2^, 30 min) confirmed the prominent antitumour efficacy against 4T1 tumours.

**Figure 34:**
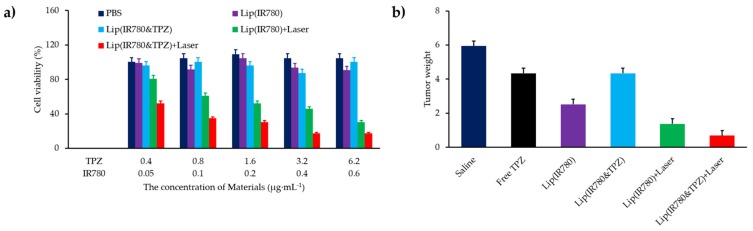
(**a**): Survival of 4T1 cells after photo-thermal treatment: Viability of cells treated with different formulations under an 808 nm laser for 3 min with a sequence of 10 s irradiation and 10 s break. (**b**): Toxicity of Lip (IR780&TPZ) in 4T1 tumour-bearing mice: Tumour weights in the different groups of mice after the indicated treatments. Adapted from Yang et al. [162].

Yang et al. [162] developed another drug delivery system for synergistic breast cancer treatment. The lipid carrier denoted Lip(IR780&TPZ) was successfully prepared by encapsulating the lipophilic IR780 in the phospholipid bilayer of liposome while TPZ was loaded in the hydrophilic core. According to both in vitro and in vivo results, (Figure 34), the lipidic carrier Lip(IR780&TPZ) could exert PDT by generating ^1^O_2_ once exposed to laser irradiation (808 nm, 1.0 W∙cm^−2^, 3 min) in 4T1 tumour, leading to the formation of a hypoxic microenvironment which activated TPZ. 

Zhang et al. [163] reported the synthesis of an innovative hypoxia-responsive 2-nitroimidazole (NI) derivative conjugated with PEG amphoteric polymer-based liposomes (PEG-NI) co-encapsulating Ce6, TPZ and a gene probe (P_miRNA_) for synergistic PDT-chemotherapy. Exposition of the multifunctional liposomes (Lip/Ce6/TPZ-P_miRNA_) to laser irradiation (670 nm, 0.48 W∙cm^−2^, 10 min) caused Ce6-mediated PDT and severe hypoxia, leading to the disassembly of the liposome and activation of the TPZ. A greatly improved anti-cancer activity compared to conventional PDT was achieved upon laser irradiation (670 nm, 0.48 W∙cm^−2^, 10 min) for both in vitro and in vivo studies in MCF-7 cell lines, indicating the benefit of the hypoxia-activated chemotherapy combined PDT of the as prepared multifunctional liposomes for synergistic treatment. 

**Figure 35:**
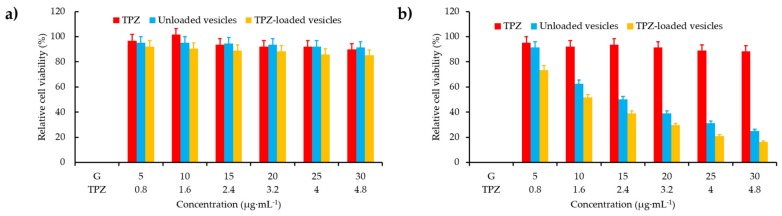
In vitro cytotoxicity of MCF-7 cancer cells incubated for 24 h with free TPZ, unloaded vesicles, and TPZ-loaded vesicles in the dark (**a**) and in light (**b**). Adapted from Wang et al. [164].

Wang et al. [164] elaborated a multifunctional supramolecular vesicles based on the recognition of water-soluble pillar [5]arene (WP5) and NIR-absorbing diketopyrrolopyrrole (DPP)-based guest (G) for combined photothermal/photodynamic/hypoxia-activated chemotherapy. These supramolecular vesicles were able to highly encapsulate TPZ. The photothermal conversion efficiency and ROS generation ability of the vesicles were investigated (660 nm, 1.5 W∙cm^−2^, 7 min). The results revealed that such vesicles could efficiently convert O_2_ to ^1^O_2_ for PDT. Meanwhile, the continuous O_2_ consumption during the PDT process resulted in hypoxic microenvironment, which triggered antitumour activity of the TPZ-loaded vesicles for synergistic enhancement of anticancer efficacy in vitro against MCF-7 cancer cells (Figure 35). 

#### 4.1.2. Doxorubicin (Dox)

**Figure 36:**
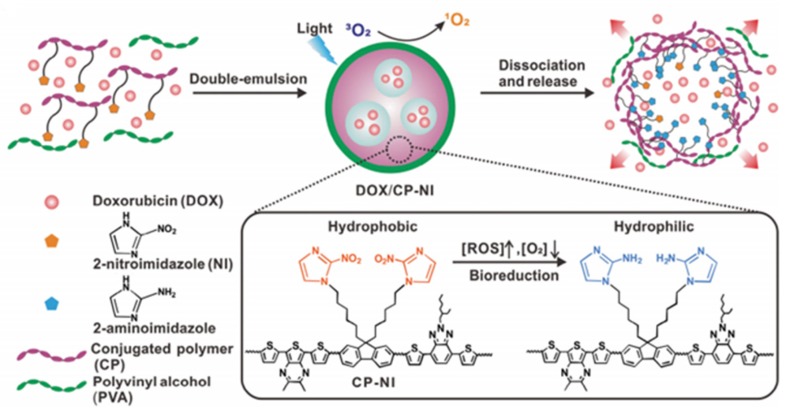
Schematic of the light-activated hypoxia-responsive drug-delivery system. Formation and mechanism of DOX/CP-NI NPs. Reprinted from [165] with permission from John Wiley and Sons, Copyright 2016.

DOX is an anthracycline that slows or stops the growth of cancer cells by poisoning TOP-2. Qian et al. [165] reported the synthesis of light-activated hypoxia-responsive NPs by combining the PDT and hypoxia responsive chemotherapy. Such a conjugated polymer-based delivery system denoted as DOX/CP-NI NPs were designed based on three components, i.e., 2-nitroimidazole-grafted conjugated polymer (CP-NI), polyvinyl alcohol (PVA) and DOX (Figure 36). 

According to both in vitro and in vivo studies on HeLa cells, DOX/CP-NI NPs could efficiently generate ROS when exposed to light irradiation (532 nm, 0.1 W∙cm^−2^, 20 min). Meanwhile, the continuous O_2_ consumption for PDT facilitate generation of hypoxic conditions which promoted the disassembly of DOX/CP-NI, and thus an efficient DOX release which resulted in enhanced anticancer effect. 

**Figure 37:**
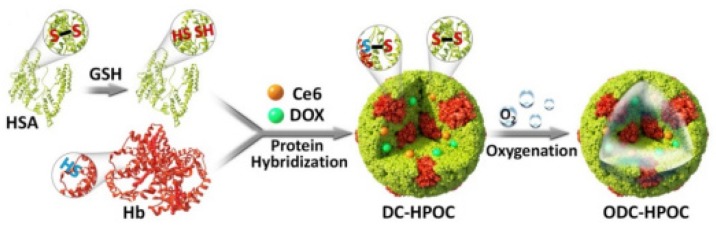
Schematic illustration of the fabrication of the ODC-HPOCs [167].

Hu et al. [166] synthesized multifunctional polymeric Ce6-DOX-MnO_2_ NPs (CDM NPs) for combined chemo and PDT enhanced by O_2_ generation. The CDM NPs were fabricated by hierarchically assembling DOX, Ce6 and colloidal MnO_2_ with poly (ε-caprolactone-co-lactide)-β- poly(ethyleneglycol)-β-poly(ε-caprolactone colactide). Once administrated through systemic injection, the CDM NPs passively accumulated in the tumour, induced decomposition of endogenous tumour H_2_O_2_ under laser irradiation (660 nm, 100 mW∙cm^−2^, 5 min) to generate O_2_ and Mn^2+^ for T1-weighted MRI. More importantly, CDM NPs dramatically improved antitumour efficiency against MCF-7 tumour-bearing mouse mode. Luo et al. [167] elaborated tumour-targeted hybrid protein O_2_ carriers loaded with DOX and Ce6 (ODC-HPOCs) (Figure 37). 

Laser irradiation (660 nm, 100 mW∙cm^−2^, 2 min) of tumour-targeted ODC-HPOCs allowed outstanding performance in tumour accumulation of O_2_, DOX and Ce6. The high O_2_ affinity of ODC-HPOCs guaranteed sufficient tumour oxygenation, which was able to break hypoxia-induced chemoresistance through inhibiting the expressions of HIF-1α, multidrug resistance 1 (MDR1) and P-glycoprotein (P-gp). Meanwhile, the abundant O_2_ enhanced the ROS generation in PDT, and the enhanced chemo-PDT (660 nm, 100 mW∙cm^−2^, 2 min) managed to offer a single-dose treatment with minimized concentration of DOX and Ce6. 

Xu et al. [168] designed mesoporous MnO_2_ (mMnO_2_)-coated UCNPs for TME-enhanced chemo-PDT and multiple imaging under NIR light excitation. The mesoporous silica shell covalently loaded with Ce6 were coated on the core–shell structured UCNPs (NaGdF_4_:Yb,Er@NaGdF_4_:Yb). Subsequently, mMnO_2_ was coated on silica shell and then modified with PEG and loaded with DOX to obtain UCNPs@Ce6@mSiO_2_@mMnO_2_-PEG-DOX (UCSM-PEG-DOX). Upon 980 nm irradiation (0.5 W∙cm^−2^, 5 min), the fast degradation of mMnO_2_ shell in TME resulted in markedly enhanced T1-contrast MRI signals, an efficient DOX release, and greatly relieved tumour hypoxia by in situ generation of O_2_. 

Xie et al. [169] developed a novel O_2_-loaded pH-responsive multifunctional nanodrug carrier UC@mSiO_2_-RB@ZIF-O2-DOX-PEGFA (URODF) for an improved chemo-PDT efficiency. NaYF_4_:Yb/Er@NaYbF_4_:Nd@NaGdF_4_ NPs (UC) were employed for dual-modal upconversion/MR imaging. Meanwhile, the core−shell structure allowed UC NPs to activate RB in the mesoporous silica shell (mSiO_2_) for PDT in 808 nm laser irradiation. Thus, under acidic conditions, the outmost O_2_ reservoir ZIF-90 shell would decompose, allowing quick release of O_2_ and DOX at low pH TME, and therefore achieving improved synergetic therapy and alleviating tumour hypoxia. Finally, according to the in vitro (808 nm, 0.5 W∙cm^−2^, 10 min) cytotoxicity against 4T1 and HeLa cells and in vivo (808 nm, 0.5 W∙cm^−2^, 5 min) tumour inhibition studies against H22 cancer cells, the URODF NPs demonstrated remarkably enhanced tumour inhibition effect. 

He et al. [170] reported a cancer-targeting vehicle characterized by cascaded reactivity to external (light) and internal (hypoxia) triggers for selective release of the cancer drug. The hypoxia- responsive drug delivery was prepared from self-assembled polyethylenimine-nitroimidazole (PEI-NI) micelles loaded with DOX that were further co-assembled with HA-conjugated Ce6 (HC) to form NPs. Upon internalization into mouse Lewis lung carcinoma (LLC) cells via CD44-mediated endocytosis, the hypoxia-responsive HC/PN/DOX NPs generated high levels of ROS under light irradiation (660 nm, 10 mW∙cm^−2^, 30 min) The continuous O_2_ consumption provoked the disassembly of the DOX-loaded PEI-NI micelles which maximized the DOX release. 

Yang et al. [171] developed a multifunctional NP composed of an iron oxide (Fe_3_O_4_) core coated to a combined shell of MnO_2_ and polypyrrole (PPy), which was both the photothermal agent and PS, to formed Fe_3_O_4_@MnO_2_@PPy. Magnetic Fe_3_O_4_ was used to increase the intracellular O_2_ concentration. DOX was loaded on the Fe_3_O_4_@MnO_2_@PPy nanocomposite to finally obtain Fe_3_O_4_@ MnO_2_@PPy-DOX NPs. The increase of O_2_ concentration was proven in presence of H_2_O_2_ with these NPs. In vitro experiments were performed on HepG2 and Chinese hamster ovary (CHO) cells to study the chemotherapeutic effect of the Fe_3_O_4_@ MnO_2_@PPy-DOX NPs for cancer and normal cells. An acidic environment-dependent of DOX release was highlighted for the NPs as well as synergistic effects of chemotherapy and PTT/PDT improved by the increase of O_2_ tumour level. An enhancement of cellular uptake and an increase of cell death was observed with the combined chemo-PTT/PDT (638 nm, 1 mW∙cm^−2^, 10 min) treatment using Fe_3_O_4_@ MnO_2_@PPy-DOX NPs.

Deng et al. [172] reported the synthesis of multifunctional nitroimidazole (NI)-bearing polymeric micelles to co-deliver DOX and Ce6 for dually hypoxia- and ^1^O_2_-responsive integration of chemotherapy and PDT. As proof of concept, in vitro and in vivo studies were investigated on 4T1 cells and 4T1 tumour-bearing mouse model, respectively. It was found, that upon 660 nm laser irradiation (100 mW∙cm^−2^, 10 min), the NCs/Dox + Ce6 produced large amount of ^1^O_2_, which caused oxidation of NI, provoking micelle collapse, triggered payload release, and the production of aldehyde which results in high PDT effect. Meanwhile, the continuous consumption of ^1^O_2_ resulted in a hypoxic environment, which triggered the micelle disassembly, DOX release, and thus caused glutathione GSH depletion that provided a supplementary anti-tumour efficacy (Table 14).

#### 4.1.3. AQ4N

**Figure 38:**
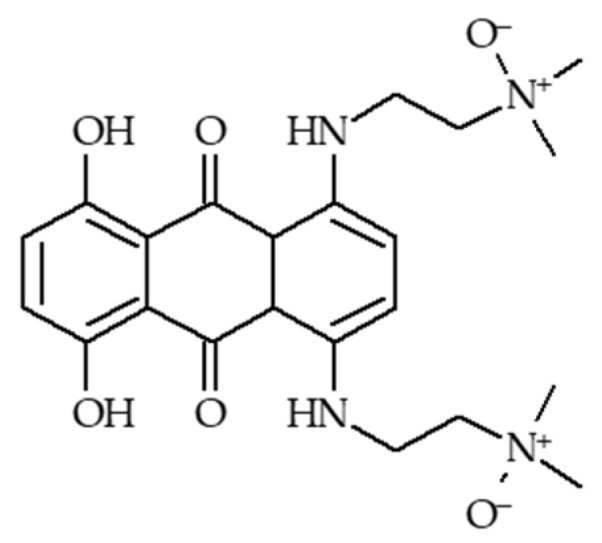
Structure of AQ4N.

AQ4N is a banoxantrone (Figure 38) and a hypoxia-activated prodrug (HAP) which can be reduced by endogenous isozymes (inducible nitric oxide synthase (iNOS) and cytochrome P450 (CYP) in hypoxia conditions.

Feng et al. [173] prepared a liposome (AQ4N-^64^Cu-*h*Ce6-liposome) encapsulating hydrophilic AQ4N and hydrophobic hexadecylamine conjugated *h*Ce6 cHeLated with a ^64^Cu isotope for in vivo positron emission tomography (PET) and a combined hypoxia-activated chemo-PDT. In vitro fluorescence imaging showed an efficient accumulation of these liposomes after intravenous injection in 4T1 cells. Moreover, an effective cell killing ability was observed after illumination (660 nm, 30 min) of cells treated with AQ4N-^64^Cu-*h*Ce6-liposome. In vivo PDT experiments (660 nm, 2 mW∙cm^−2^, 1 h) were performed in 4T1 tumour-bearing mice injected with AQ4N-^64^Cu- *h*Ce6-liposome and a severe tumour hypoxia was observed triggering the activation of AQ4N and thus improved the inhibition of tumour growth. 

A graphene oxide (GO)-based NP was designed by Luan et al. [174] for a trimodal cancer therapy. This NP was composed of verteporfin (VP) and the peptide c(RGDfK) for vascular-targeted PDT, AQ4N as hypoxia-activated prodrug (HAP), and HIF-1α siRNA (siHIF-1α) to suppress the HIF-1α expression upon hypoxia and thus increase the AQ4N activation. These NPs significantly hindered the growth of tumours in PC-3 models in vivo after illumination (690 nm, 30 mW∙cm^−2^, 10 min) compared to the control groups (Table 15). 

**Figure 39:**
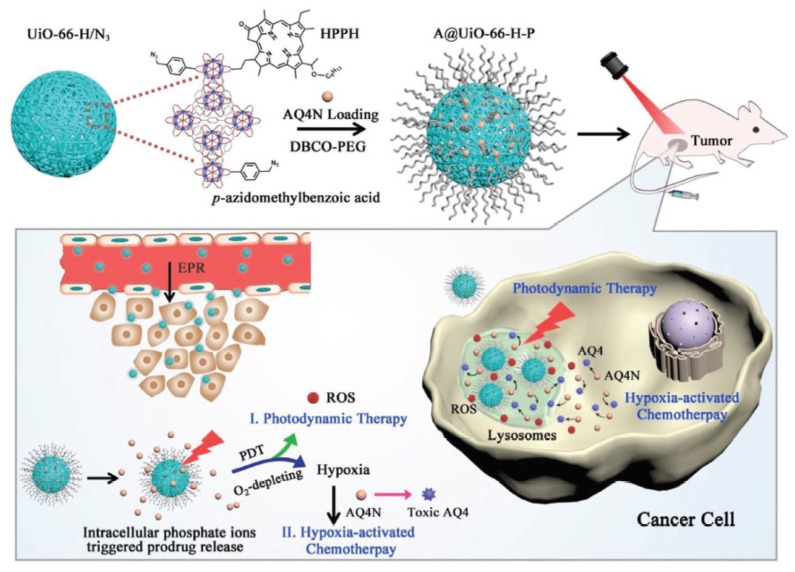
Schematic illustration showing the synthetic procedure of A@UiO-66-H-P NPs and the mechanism of PDT and hypoxia-activated cascade chemotherapy. Reprinted from [175] with permission from John Wiley and Sons, Copyright 2018.

He et al. [175] engineered NPs for hypoxia-activated chemo-PDT composed of pegylated UiO-66 NMOFs co-anchoring a PS photochlor (HPPH) and an azide group and encapsulating AQ4N (Figure 39). 

In both in vitro and in vivo studies (U87MG cells and tumour, 671 nm, 100 mW∙cm^−2^, 6 min) the authors observed that the O_2_-depleting PDT with these NPs conducted to an intracellular/tumour hypoxia leading to the activation of AQ4N and thus an efficient synergistic chemo-PDT therapy (Table 15). 

#### 4.1.4. Platinium drugs

Platinium (IV) complexes are prodrugs which can be reduced in toxic Pt (II), by UV light irradiation or reducing agents. These species act as drugs for chemotherapy. Guo et al. [176] reported a Pt(IV) complex-based photoactivatable polyprodrug able to simultaneously generate highly toxic Pt(II) species for chemotherapy and a high level of ROS. This chemo-PDT concept did not rely on the use of a PS and the consumption of O_2_. The polyprodrug was obtained by co-polymerizing Pt(IV) complex-based prodrug monomer (PPM) with 2-methacryloyloxyethyl phosphorylcholine (MPC), nanosized hydrogel-like polyprodrug. Under light exposure, a reduction of Pt(IV) moieties contained in this photoactivatable polyprodrug was observed leading to the generation of Pt(II) species. In vitro experiments were performed in A549 cancer cells and in vivo in nude mice bearing A 549 cells and IC_50_ values for polyPPM of 2.6 mM and 2.9 mM against A549 and cisplatin-resistant A549R cells respectively were observed under irradiation (396 nm, 5 mW∙cm^−2^, 5 min). In vivo A549 tumour- bearing mice were treated with polyPPM and light irradiation (0.4 W∙cm^−2^, 10 min) and resulted in the decrease in tumour growth. 

The same strategy was described by Xu et al. [177]. They developed a NP containing Pt(IV) and Ce6, loading with UCNPs for the conversion of 980 nm near-infrared light into 365 nm and 660 nm emissions. After tumour accumulation, the NPs were triggered by a 980 nm laser to generate O_2_ and could also release active Pt(II). Results suggested that NPs generated O_2_ inside HeLa and L929 cells (980 nm, 0.85 W∙cm^−2^, 5 min). In vivo in HeLa, B16 (Murine tumour cell line skin cancer), HCT116 (Human colon cancer cell line) and MDA-MB-231 tumour model, the capability of NPs to improve tumour hypoxia was demonstrated (980 nm, 0.80 W∙cm^−2^, 10 min). 

A new kind of compounds (covalent-organic polymers, COPs) which are able to cross-linked differents organic molecules by covalent bond to form organic network structures have been used in various fields. Wang et al. [178] cross-linked a PS, mesotetra(p-hydroxyphenyl) porphine (THPP), to a chemotherapeutic pro-drug, cis-Pt (IV) and conjugated to PEG to obtain THPP-Pt-PEG COPs. Cis-Pt(IV) was also used as a reduction-responsive linker. The ability of THPP-Pt-PEG COPs to kill cancer cells after PDT (660 nm, 5 mW·cm^−2^, 20 min) was observed in 4T1 cells and a reduction-responsive degradation/drug release was highlighted. After injection of THPP-Pt-PEG COPs in 4T1 tumour-bearing mice, the combined chemo-PDT (660 nm, 5 mW·cm^−2^, 45 min) of the COPs showed a n improvement of therapeutic outcome in comparison with PDT and chemotherapy taken separately (Table 16).

### 4.2. Antiangiogenic-PDT

PDT also induces expression of angiogenic and survival molecules including VEGF, cyclooxygenase-2 (COX-2), and MMPs. The founding member of the hypoxia-inducible factor (HIF) family, HIF-1α, regulates a broad array of genes in response to O_2_ deprivation.

#### 4.2.1. HIF-1α inhibitors

The expression of HIF-1α is increased after PDT treatment of cancer cells, which induces PDT resistance. The inhibition of HIF-1α combined to PDT can lead to an improvement of the PDT efficiency. Chen et al. [179] developed anisamide-targeted lipid–calcium–phosphate (LCP) NPs encapsulating HIF-1α siRNA to reduce the expression of HIF-1α prior to PDT treatment with Photosan^®^. In vitro in SCC4 and SAS cells, targeted LCP with anisamide showed an efficient release of HIF-1α siRNA 2.5 or 3.5 fold higher than observed for LCP without anisamide.

Treatment with these NPs prior to photosan-PDT (640 nm, 320 mW∙cm^−2^, 100 J∙cm^−2^) led to a significant decrease of cell viability in comparison with PDT alone. In vivo in SCC4 and SAS tumour-bearing mice, the combination of HIF-1α siRNA and PDT led to a significant decrease of tumour volume (40 %) after 10 days. 

Sun et al. [180] designed a multifunctional compound (siHIF@CpMB) constituted by cationic porphyrin lipip microbubbles (CpMBs), elaborated from cationic porphyrin lipip NPs, and loading HIF-1α siRNA (siHIF) on the surface by electronic adsorption. In vitro in MDA-MB-231 cells, the authors observed an efficient release of siHIF after ultrasound exposure (1.03 MHz, 50 % duty, 1 W∙cm^−2^, 1 min) and production of ^1^O_2_ after irradiation (650 nm, 200 mW∙cm^−2^, 10 min). Moreover, the combinaison of siHIF treatment and PDT led to significant effect to kill cancer cells in both in vitro and in vivo models. 

Broekgaarden et al. [181,182] elaborated a strategy relying on the use of acriflavine (ACF) to inhibit the expression of HIF-1α in combinaison with PDT treatment with ZnPC-ETLs (Zinc Phthalocyanine in endothelium-targeting liposomes). An increase of HIF-1α expression has been observed with PDT alone. The authors observed an increase of PDT effect (500 mW, 15 J∙cm^−2^) with the addition of ACF before PDT in both normoxic and hypoxic conditions in vitro in human perihilar cholangiocarcinoma models (SK-ChA-1 cells). They reproduced the experience in A431 cells and observed an increase of cell death in hypoxic conditions after combination of ACF and PDT (671 nm, 500 mW∙cm^−2^, 15 J∙cm^−2^) whereas no adjuvant effect of ACF was highlighted in normoxic conditions. (Table 17). 

#### 4.2.2. VEGF Inhibitors

Vascular endothelial growth-factor (VEGF) is overexpressed in tumour after PDT treatment and is involved in the neovascularisation of tumours which can induce PDT resistance. The inhibition of VEGF during PDT can lead to an enhancement of its efficiency.

Ferrario et al. [183] studied the overexpression of HIF-1α and VEGF after PDT treatment (570–650 nm, 0.35 mW∙cm^−2^) with Photofrin of BA tumours (mouse mammary carcinoma). To reduce this overexpression and thus improving the efficiency of PDT, they combined antiangiogenic therapy with PDT by treating mice with an angiogenic synthetic dipeptide (IM862) or an endothelial-activating polypeptide (EMAP-II) to inhibit VEGF production. The authors observed a decrease of VEGF level after combined antiangiogenic-PDT therapy (630 nm, 75 mW∙cm^−2^, 200 J∙cm^−2^) with IM862 or EMAP-II and Photofrin as well as an increase of the tumoricidal action of PDT. 

Zhou et al. [184] examined the influence of antiangiogenic coumpounds (VEGF inhibitors: SU5416 and SU6668) in combination with PDT with hypericin. In vivo studies in CNE2 tumour-bearing mice (poorly differentiated nasopharyngeal carcinoma) showed a significant inhibition of tumour growth for mice treated with SU5416 or SU6668 in combination with PDT (halogen light source, 47.7 J∙cm^−2^, 60 mW∙cm^−2^) with a better enhancement of the tumour response to PDT with SU6668.

Weiss et al. [185] compared the used of two types of compounds: an anti-VEGF antibody (bevatizumab) and angiostatic tyrosine kinase inhibitors (TKIs, sunitinib, sorafenib and axitinib) to inhibit VEGF receptor for an antiangiogenic treatment in combinaison with PDT using visudyne. They chose two tumour models (A278 human ovarian carcinoma cells and HCT-116 human colorectal carcinoma cells) implanted in chorioallantoic membrane of the chicken embryo. They observed the best improvement of PDT (35 mW∙cm^−2^, 5 J∙cm^−2^) by combination with TKIs especially sorafenib and axitinib, the last one leading to a complete suppression of VEGFR-2 receptors expression in the vasculature of the tumour. The use of bevacizumab did not lead to any improvement of PDT.

Lecaros et al. [186] combined PDT using Photosan^®^ with lipid-calcium–phosphate NPs (LCP NPs) delivering VEGF-A small interfering RNA (siVEGF-A) in cells to enhance the PDT effect by decreasing VEGF-A expression. In vivo studies in human oral squamous cancer cell (HOSCC), SCC4 and SAS models were performed and the combinaison of siVEGF-A and PDT (640 nm, 320 mW∙cm^−2^, 100 J∙cm^−2^, 11 min) induced a significant reduction of tumour volume in both models.

**Figure 40:**
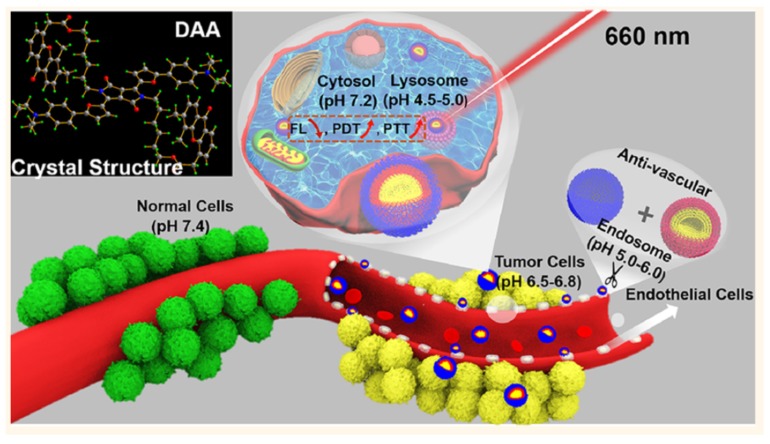
Antivascular and pH-Responsive Cancer PTT/PDT of DAA NPs at the tumour site. Reprinted from [187] with permission from the American Chemical Society, Copyright 2018.

Liang et al. [187] synthesized pH-responsive direct-acting-antiviral (DAA) NPs with an average diameter of 55 ± 2 nm and composed of dimethylxanthenone-4-acetic acid (DMXAA) as active-targeting VEGF receptor and diketopyrrolopyrrole (DPP-4) as PTT/PDT agent to improve combined PTT/PDT treatment by the destruction of the vascular region of tumours (Figure 40). Under acidic conditions of the TME, there was an effective release of DMXAA and an increase of ^1^O_2_ generation as well as a good photothermal effect in comparison with pH of 7.4. In vitro and in vivo studies in HeLa tumour model highlighted an efficient synergitic effect of antivascular activity of DMXAA and PTT/PDT (660 nm, 0.8 W∙cm^−2^, 4 min) to kill cancer cells with a complete ablation of tumour (Table 18). 

#### 4.2.3. Others 

Cyclooxygenase 2 (COX-2) expression is induced by PDT treatment and it is involved in the progression of cancer. In 2005, Ferrario et al. [188] evaluated the effect of COX-2 inhibitors (celecoxib, a COX-2 selective nonsteroidal anti-inflammatory drug or NS-398, a COX-2 inhibitor) combined to PDT with Photofrin on cancer cell death in comparison with PDT alone. In vitro in BA cells, they observed a light dose-dependent increase of apoptosis of cancer cells after combined PDT (570–650 nm, 0.35 mW∙cm^−2^, 0 to 525 J∙cm^−2^, 0 to 150 s) with COX-2 inhibitors. In vivo (630 nm, 75 mW∙cm^−2^, 0 to 200 J∙cm^−2^) injection of COX-2 inhibitors decreased angiogenesis and inflammation and increased PDT efficiency.

Tuncel et al. [189] synthesized a phthalocyanine−chalcone conjugate composed of chalcones holding properties of vascular disrupting agents (VDA) and a phthalocyanine as the PS. They observed a moderate inhibition of HUVEC (human umbilical vein endothelial cell) proliferation after treatment with the conjugate lower than those observed with chalcone alone as well as a lower ^1^O_2_ for the conjugate in comparison with the PS alone (55% against 83%). However, PDT treatment (red light, 3.6 J∙cm^−2^) with the conjugate in vitro in human colon adenocarcinoma cells (HT-29) led to the best efficiency, which could be explained by a better cellular uptake thanks to its amphiphilic character.

The activation of the nuclear factor-kappa B (NF-κB) is induced by PDT treatment and has a negative role for this last one. Li et al. [190,191] studied the combination of dihydroartemisinin (DHA), which could inactivate NF-κB and displayed an anticancer activity, with PDT using 5-ALA in comparison with PDT alone. In vitro and in vivo in human oesophageal cancer models (Eca109 and Ec9706), they showed a better tumour inhibition with a reduction of NF-κB expression for the combined treatement with DHA and PDT (630 nm, 20–25 J∙cm^−2^) as compared to each treatment alone. 

**Figure 41:**
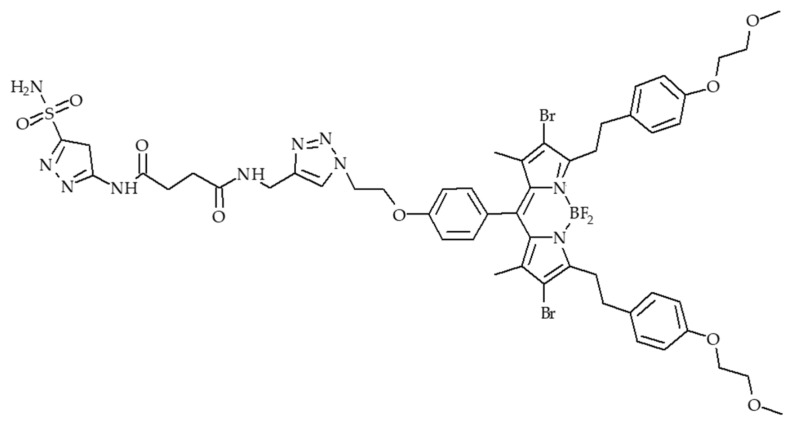
Structure of AZBPS. Adapted from Jung et al. [192].

Carbonic anhydrase IX (CAIX) is overexpressed in tumour and is involved in the tumour survival and invasion. Jung et al. [192] synthesized an acetazolamide-functionalized boron dipyrromethene PS (AZ-BPS) (Figure 41) to combine antiangiogenic therapy by inhibiting CAIX with PDT. 

In vitro in aggressive cancer MDA-MB-231 cells, they evaluated the phototoxicity (660 nm, 2 W∙cm^−2^, 30 min) of AZ-BPS in comparison with BPS alone and observed an enhancement of phototoxicity. The same observation was done in vivo in MDA-MB-231 tumour-bearing mice, which may be due to the inhibition of tumour angiogenesis combined to PDT (Table 19).

### 4.3. PTT/PDT

Photothermic therapy is a technique to destroy cancer cells by burning them selectively. This technology uses a low-power laser beam that is directed to the tumour containing small amount of molecules capable of absorbing near-infrared rays (between 800 and 1300 nm), and which efficiently convert this energy into heat. Since this technique uses irradiation, some teams considered associating PTT with PDT and proposed targeted hybrid nano-objects capable of being excited to produce both heat and ROS. This dual PTT/PDT modality offers the advantage of using only one NP for a double effect by involving only light. In contrary to PDT, PTT does not require O_2_ to be efficient.

**Figure 42:**
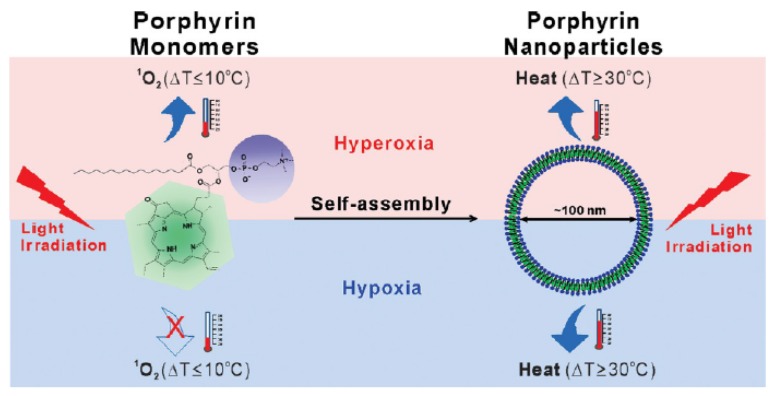
Schematic illustration of the study rationale and design. Reprinted from [193] with permission from the American Chemical Society, Copyright 2013.

Nanostructured self-quenched porphysome NPs (Figure 42) have been developed by Jin et al. [193]. PDT with Photofrin (633 nm, 200 mW with a spot size of 9 mm diameter for 318 s) in treating mice bearing KB xenograft under hyperoxic conditions induced 100% reduction of tumour volume 2 days after treatment and 100% survival over 50 days. When molecules of Photofrin were assembled in the bilayer of self-quenched porphysome NPs, no PDT effect could be observed (671 nm, 200 mW, 5 min 18 s). Porphysomes proved in fact to be photothermal enhancers that induced rapid and significant tumour temperature increase (T final > 60 °C in 85 s) upon PTT irradiation (PTT: 671 nm, 750 mW, 100 J∙cm^−2^, 85 s) for a complete KB tumour elimination regardless of cellular O_2_ amount in both hyperoxic and hypoxic conditions. 

Zhu et al. [194] designed a NP (BSA/SAs–NMOF NPs) based on NMOFs (TCPP as organic ligands and iron ions as metal center) as PTT/PDT agent, covalently conjugated to BSA/SA complexes (bovine serum albumin/sulfonamides) for biocompatibility. In vitro in 4T1 cell line, the authors observed a photothermal conversion efficiency of 40.53% (660 nm, 50 mW∙cm^−2^, 10 min) and a efficient ROS generation (660 nm, 50 mW∙cm^−2^, 10 min then 1.0 W∙cm^−2^, 5 min) in both normoxic and hypoxic conditions under laser irradiation. The synergistic effect of PTT/PDT (660 nm, 50 mW∙cm^−2^, 10 min then 1.0 W∙cm^−2^, 5 min) to kill cancer cells was desmontrated in both in vitro and in vivo 4T1 tumour models and appeared to be significantly better than PTT or PDT alone. 

In 2016, Hu et al. [195] developed an original strategy based on the increase of temperature to improve circulation of the blood, blood O_2_ concentration in tumour tissue. This strategy is different from PTT since it does not base on a conversion of energy into heat but on an external change of the temperature of mice. They synthesized a human serum albumin loaded with Ce6 cHeLated with Mn^2+^ (HSA-Ce6 NAs) with an average hydrodynamic diameter of 100±2.4 nm. Reduced GSH could induce the cleavage of intermolecular disulfide bonds of HSA-Ce6 NAs and the release of Ce6. In vitro in 4T1 cells, they showed that PDT (660 nm, 50 mW∙cm^−2^, 5 min) led to cell viability of 25.7 % at 37 °C and 5.8 % at 43 °C. In vivo in 4T1 tumour-bearing mice, PDT was also performed (660 nm, 200 mW∙cm^−2^, 20 min). The designed a warm water bath to simulate « hot spring » bath. By changing the temperature from 37 °C to 43 °C, blood flow velocity increased from 17.3 cm∙s^−1^ to 32.4 cm∙s^−1^, O_2_ saturation in tumour tissue also increased from 52% to 79%. After PDT, mice treated with Ce6 at 37 °C showed a tumour growth delay, with HSA-Ce6 NAs at 37 °C a partly inhibition of tumour growth whereas with HSA-Ce6 NAs at 43 °C they could observe significant tumour regression with no tumour recurrence.

**Figure 43:**
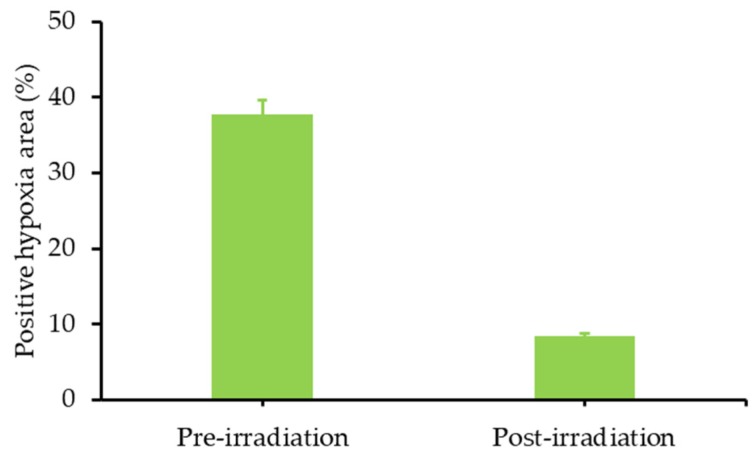
In vivo activation of DiR-hCe6-liposome by NIR laser irradiation and the followed tumour Oxygenation. Semi-quantitative analysis of the percentage of positive hypoxia region before and after laser irradiation. Adapted from Feng et al. [196].

In 2017, Feng et al. [196] described liposomes activatable by NIR light to relieve tumour hypoxia and to be an efficient PDT-agent while protecting skin. The liposomes (DiR-*h*Ce6-liposomes) were composed by a PEG shell and encapsulated *h*exylamine conjugated Ce6 (*h*Ce6) and 1,1′-dioctadecyl-3,3,30,3′-tetramethylindotricarbocyanine iodide (DiR) as a NIR dye quenching the photophysical properties of *h*Ce6. In the absence of NIR light, *h*Ce6 contained in DiR-*h*Ce6-liposome presented a quenching of its photophysical properties and under a 785 nm NIR irradiation, a photobleaching of DiR induced a recovery of *h*Ce6 properties but also a photothermal heating leading to a reduction of tumour hypoxia. A quenching of 97% of *h*Ce6 fluorescence in DiR-hCe6-liposomes was observed and a recovery of fluorescence was observed after NIR irradiation (785 nm, 1 W∙cm^−2^, 10 min). The ^1^O_2_ generation was also evaluated under light excitation (660 nm, 2 mW∙cm^−2^, 30 min) and the production by DiR-*h*Ce6-liposomes was significantly lower than Ce6 alone and *h*Ce6-liposomes but under NIR irradiation followed by 660 nm irradiation, DiR-*h*Ce6-liposomes recovered this ability. In vitro PDT efficiency was investigated in 4T1 cells. Under light irradiation (660 nm, 2 mW∙cm^−2^, 30 min), DiR-*h*Ce6-liposomes showed a slight phototoxicity but a succession of NIR and 660 nm irradiation, a high phototoxic effect was observed similar to that observed for *h*Ce6-liposomes. In vivo studies in balb/c mice bearing 4T1 tumours under NIR irradiation, an increase of temperature over time was observed corresponding to a photothermal heating. A significant reduction of tumour hypoxia from 38% to 12% was also observed after NIR irradiation (Figure 43).

**Figure 44:**
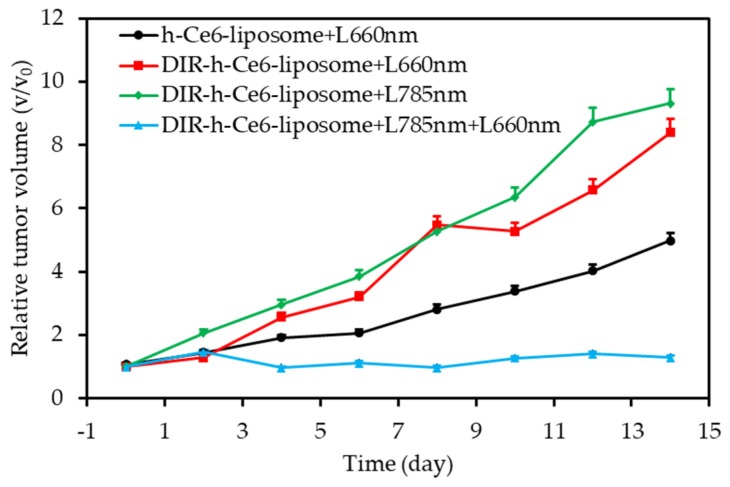
In vivo NIR light activated synergistic cancer phototherapy. Relative tumour volume (V/V_0_) changing curves of mice after various different treatments at indicated for 14 days. V and V_0_ stood for the tumour volumes after and before the treatment, respectively. Adapted from Feng et al. [196].

Finally, the in vivo PDT effect of DiR-hCe6-liposomes after successive irradiations (785 nm, 0.7 W∙cm^−2^ for 20 min followed by 660 nm, 2 mW∙cm^−2^ for 1h) was studied and a high inhibition of tumour growth was observed whereas for mice injected with *h*Ce6 and irradiated at 660 nm, only a moderate effect was observed (Figure 44 and Table 20). 

### 4.4. Imuno-PDT

Cancer cells undergo profound genetic rearrangements that allow them to acquire their malignant properties. Thus, they begin to express on their surface specific molecules (tumoural antigens) that distinguish them from healthy cells and are able to induce immune reactions. Immunotherapy is a therapeutic approach that acts on the immune system of a patient to fight against his disease. This technique relies on the injection of specific proteins to the targeted tumour, the goal being to teach the patient’s immune cells to identify tumour and eliminate it. PDT may be a synergistic adjunct to this type of treatment as shown by the following example.

**Figure 45:**
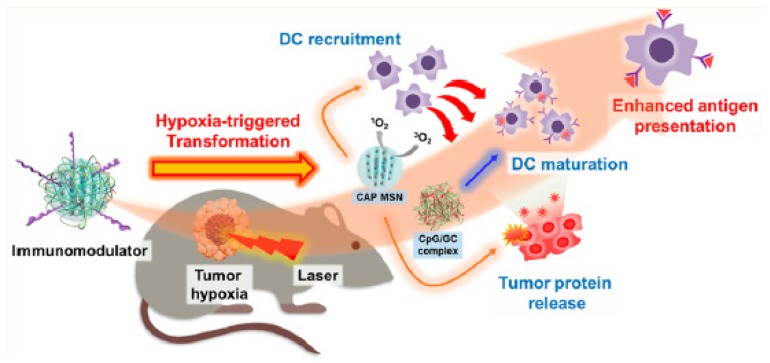
Schematic representation of Immuno-PDT concept of the study. Reprinted from [197] with permission from the American Chemical Society, Copyright 2019.

Im et al. [197] developed hypoxia-responsive Ce6-doped-azobenzene-glycol chitosan(GC)-PEG mesoporous silica NPs (CAGE) to enhance cancer immunotherapy (CIT) with PDT by modulation of dendritic cells and destruction of cancer cells (Figure 45). The effective generation of ROS by CAGE under laser irradiation was confirmed using SOSG. Biocompatibility and photoinduced cytotoxicity (660 nm, 150 mW∙cm^−2^, 15 min) of carriers was validated in vitro in a murine melanoma cell line (B16.F1). In vivo studies in B16.F1 tumour bearing mice showed an efficient inhibition of tumour growth for mice treated with CAGE and irradiated at 660 nm (200 mW∙cm^−2^, 15 min).

## 5. Hypoxia-Independent PDT 

Intratumoural hypoxia is one of the major limitations in clinical use of PDT owing to insufficient generation of ^1^O_2_. This hypoxic environment is further aggravated by consumption of oxygen during PDT limiting the therapeutic outcome [193,198]. To overcome that problem, various oxygen-sufficient materials or oxygen-independent PSs to produce ROS are developed and presented in this section.

### 5.1. PDT Type I

The principle of PDT is based on a PS excitation at a specific wavelength enabling PS to move to its triplet excited state and leading to the formation of cytotoxic ROS. These ROS can be produced by Type I (i.e. free radicals such as ^●^OH) or Type II (i.e. ^1^O_2_) photochemical reactions simultaneously. Most of the clinical applications relating to PDT are based on Type II PDT and suffering badly from the insufficient O_2_ supply in TME. One of the approaches being explored to circumvent this problem is the use of Type I PDT for more cytotoxic free radicals’ generation to reduce dependence on intracellular oxygen content.

**Figure 46:**
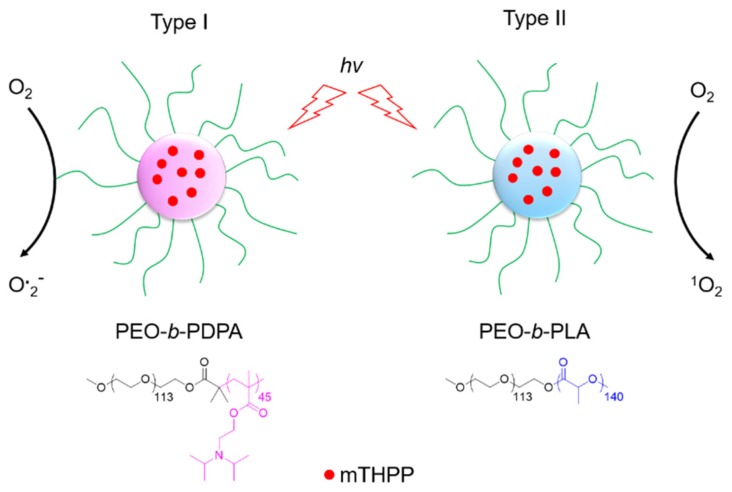
Modulation of photoactivation mechanism of a model PS, mTHPP, by the micelle microenvironment. Electron-rich PDPA micelles led to increased type I reactions producing superoxide radical anions, while electron-deficient PLA micelles generated ^1^O_2_ as predominant species by type II reactions. Adapted from Ding et al. [199].

Ding et al. (2011) [199] designed micelles with 5,10,15,20-tetrakis-(*meso*-hydroxy- phenyl)porphyrin) (mTHPP) effective under both normoxic (50 mm Hg O_2_) and hypoxic (less than 20 mm Hg O_2_) conditions by modulation of photoactivation mechanism. Diameter of such micelles were 57 nm (electron rich: β-poly(2-(diisopropylamino)ethyl methacrylate (mTHPP-PDPA) micelles), 49 nm (electron deficient: poly(D,L-lactide)) micelles (mTHPP-(PEG-b)PLA) (Figure 46).

In vivo experiments were performed with various cancer cells (H2009, A549 and PC-3) in hypoxic conditions, irradiated at 532 nm (20 mW∙cm^−2^, 10 min) and cell survivals was observed 2 days after treatment. PDPA micelles generate less ^1^O_2_ (relative quantum yield of 0.46) than PLA micelles in air saturated solution but about three time more others ROS. As example, survival of H2009 cells were two folds lower with PDPA micelles than with PLA micelles and three folds lower than with free mTHPP.

In 2014, Usacheva et al. [16] designed MB-loaded alginate NPs (275±30 nm) to study the PDT efficiency under normoxic and hypoxic conditions. The authors compared the ROS and ^1^O_2_ production with free MB and MB NPs after irradiation under different concentration of O_2_. The ^1^O_2_ production and fluorescence were systematically higher for MB NPs than free MB even at low O_2_ concentration. More interesting was a higher production of ROS with the NPs than free MB. Nitroblue tetrazolium (NBT) assay clearly indicated a high level of O_2_^−●^. Cytotoxicity of MB and MB NP-mediated PDT was studied in various breast cancer cells (MDAMB231, 4T1, SKBR3, and MCF7 cells) under ambient conditions and NPs showed a better efficacy for the MB. A similar study in normoxia and hypoxia led to the same result. The authors investigated the MB-mediated PDT (660 nm, 6 mW∙cm^−2^) on mammospheres induced by MCF-7 cancer stem cells (CSCs) and showed that MB NPs effectively disrupted the formation of mammospheres as well colonies of cells under hypoxic conditions. 

**Figure 47:**
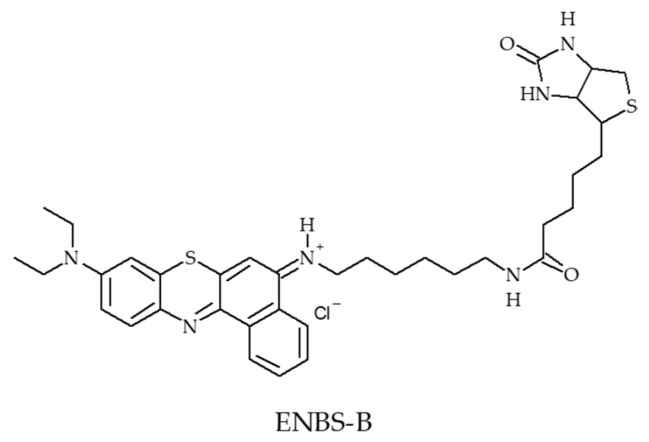
Structure of ENBS-B. Adapted from Li et al. [200].

**Figure 48:**
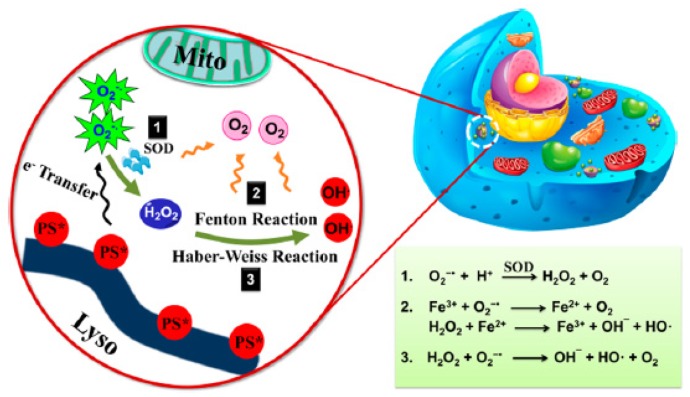
Schematic Illustration of Photo-Induced Radical Generation Mechanism of ENBS-B. Reprinted from [200] with permission from the American Chemical Society, Copyright 2018.

Li et al. (2018) [200] developed a molecular superoxide radical generator (ENBS-B) (Figure 47) that excited by near infrared light produced through type I reaction O_2_^−●^. A significant part of this O_2_^−●^ was transformed in ^●^OH after formation of H_2_O_2_ (Figure 48).

In vitro cytotoxicity of HepG2 cells under normoxic and hypoxic conditions was increased at equal time up to 94 % at low concentration of ENBS-B. In vivo studies were performed on tumour bearing BALB/c mice with light irradiation (660 nm, 15 min, 14.4 J∙cm^−2^) after injection of ENBS-B A They could observed the suppression of tumour growth. This was probably due to a better targeting on biotin receptors and also to the overcoming of traditional PDT involving O_2_ consumption.

**Figure 49:**
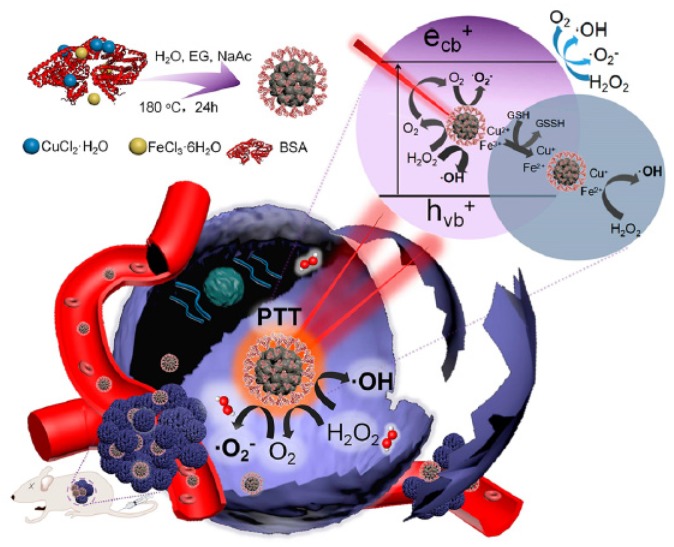
Schematic illustration of synthetic process and therapeutic mechanism of CFNs. Reprinted from [201] with permission from the American Chemical Society, Copyright 2018.

Liu et al. (2018) [201] irradiated copper ferrite nanosphere (CFNs) (Figure 49) with 650 nm laser, in synergy with PTT. PDT and Fenton reaction together allowed the theranostic nanoplatform to generate cytotoxic ^●^OH and O_2_^−●^ by electron/hole pairs of CFN. This platform was synthesized using BSA as biocompatible surfactant and had a diameter of 70 nm. 

In vitro in HeLa cells treated with CFNs and light illumination (650 nm, 15 min, 0.469 W∙cm^−2^) of CFNs induced ROS formation, with higher concentration than with light alone or CFNs alone. The CFNs were proven to regulate the TME via the catalysis of H_2_O_2_ producing O_2_ and consuming GSH. The authors observed the decrease of tumour hypoxia using ([Ru(dpp)_3_]Cl_2_. Cell viability decreased nearly to zero when concentration of CFNs increased after two different illuminations (650 nm, 15 min, 0.469 W∙cm^−2^ and 808 nm, 10 min, 1.3 W∙cm^−2^). In vivo experiments were performed on U14 tumour bearing mice, leading to a complete ablation of tumour when both PDT and PTT were in synergy.

**Figure 50:**
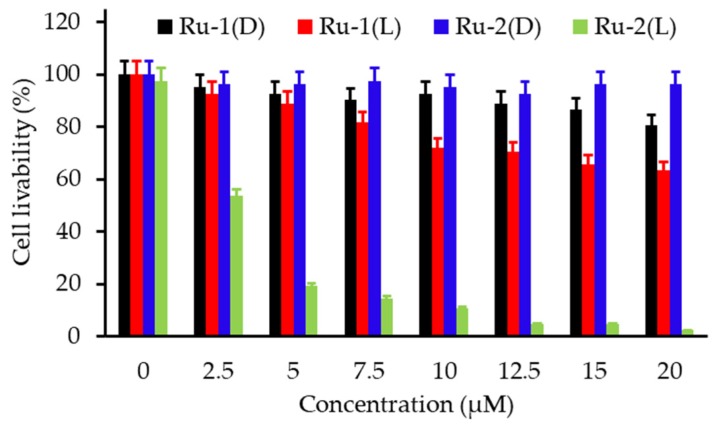
Dose–dependent curves for cell viability of HeLa cells treated with Ru1 and Ru2 using a typical MTT assay under light irradiation (L) or in the dark (D). Cells were irradiated with white light (400–800 nm, 30 mW∙cm^−2^, 10 min). Adapted from Lv et al. [202].

Lv et al. [202] synthetized type I PSs based on ruthenium (II) complexes with different cyclometaled ligands, one with 2,4-difluorophenylpyridine (Ru1) and the other with a coumarin (Ru2). In both normoxic and hypoxic conditions, experiments showed a strong effect attributed to charge transfer followed by type I reaction, leading to ROS production. It was found that ROS production (475 nm, 10 mW∙cm^−2^) by Ru2 was 6.7 time larger than by Ru1. In vitro PDT effect was evaluated in HeLa cells irradiated in the visible region (400–800 nm, 30 mW∙cm^−2^) during 10 min. Ru2 was clearly more aggressive than Ru1 even at low concentration (Figure 50). Moreover, under hypoxia, the quantity of apoptotic cells after treatment was 2.83 % for Ru1 and 54 % for Ru2.

In vivo PDT treatment (Xe lamp) on HeLa tumour bearing mice using Ru2 as PS indicate a tumour weight going from about 2 g to less than 0.1 g after 14 days.

**Figure 51:**
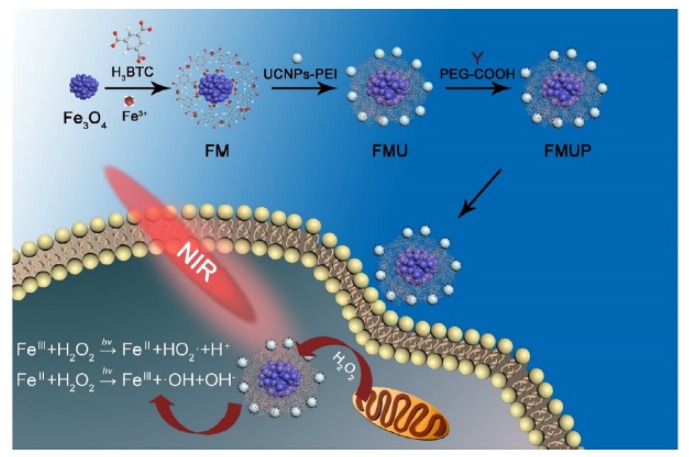
Schematic illustration of the synthesis process of FMUP, and the intracellular photon-Fenton reaction of FMUP with intracellular H_2_O_2_ under the irradiation of 980 nm. Reprinted from [203] with permission from Elsevier Ltd, Copyright 2018.

Wang et al. (2018) [203] designed another nanoplatform Fe_3_O_4_@MIL-100(Fe)-UCNP (FMU) (MIL, Material of Institute Lavoisier) which generate ^●^OH species in large amount due to a photo Fenton reaction. PEGylated FMU were also synthesized (FMUP) (Figure 51). In vitro cytotoxicity was measured on HeLa cells excited by 980 nm laser (0.9 W∙cm^−2^, 10 min) and the cell viability was about 10 %.

In vivo experiments were performed on mice transplanted with carcinoma cells U14. Starting from a tumour of about 6 mm of diameter, illumination during 15 min at 980 nm (0.9 W∙cm^−2^) was followed after 14 days of treatment by a significant inhibition of growth and even a small decrease of the initial tumour diameter. Synergy between PDT and Fenton photoreaction was clearly the reason of such a result.

**Figure 52:**
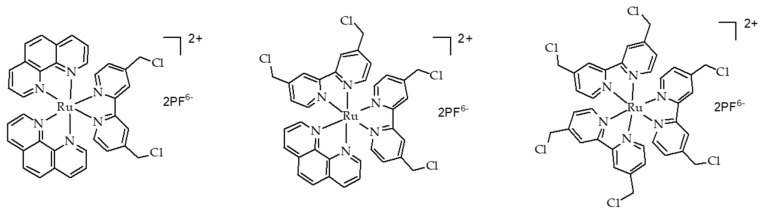
Chemical structures of complexes Ru (II) complexes. Adapted from Tian et al. [204].

Tian et al. (2018) [204] integrated chloromethyl group into a Ru(II)-bipyridine complex leading to three chemical structures showed in Figure 52. Known to accumulate easily into mitochondria these complexes generate radicals in presence of nicotinamide adenine dinucleotide (NADH) abundant in such organelles. These radicals induced strong damages on DNA even in hypoxic conditions, leading to apoptosis. In vitro, the cytotoxicity toward SKOV-3 was evaluated, after incubation at different concentrations and irradiation at 470 nm (LED) during 30 min. Complex 3 induced the highest cytotoxicity (between 50 and 10 % of viability). Under hypoxic condition (3% O_2_), this complex induced 21.1 % of apoptotic cells after 30 min of irradiation (470 nm, 22.5 mW∙cm^−2^). A disadvantage of this method is clearly that complexes were consumed during the PDT treatment, which could be solved by integration of Ru complexes into a drug delivery platform.

**Figure 53:**
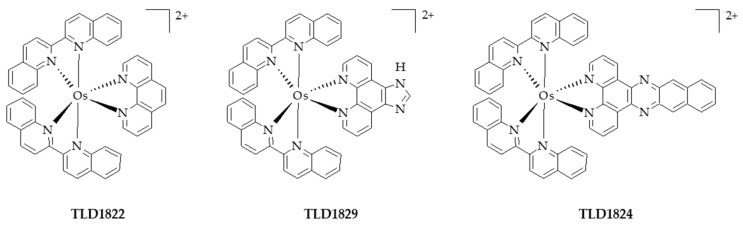
Molecular structures of the osmium-based PSs. Adapted from Lazic et al. [205].

Lazic et al. [205] developed three panchromatic PSs (TLD1822, TLD1829 and TLD1824) based on osmium complexes containing different 2,2’-biquinoline (biq) ligands (Figure 53) for PDT applications in both normoxic and hypoxic environment. These compounds presented the particularity to be activatable from 200 to 900 nm and the evaluation of ^1^O_2_ generation by these compounds showed very low ϕ_Δ_ (~ 4%) for TLD1822 and TLD1829 and no production of ^1^O_2_ was observed for TLD1824 indicating PDT effect with these molecules was not mediated by ^1^O_2_ but by other ROS. 

In vitro PDT efficiency was evaluated in both HT1376 (human bladder cells) and U87 (human glioblastoma cells) under NIR (808 nm, 900 mW∙cm^−2^ for 667 s) or red (625 nm, 450 mW∙cm^−2^ for 200 s) light irradiation. The workers first evaluated PDT efficiency in normoxic conditions and observed that all PSs under both irradiations, TLD1829 displayed the highest cytotoxicity in U87 cells whereas for TLD1822 highest cytotoxicity was found in HT1376 cells. Moreover, no significant influence of the type of irradiation was highlighted. In hypoxic conditions, the cytoxicity of TLD1822 under irradiation was similar to that observed in normoxic conditions contrary to a PS of reference (PPIX) and TLD1829, which losted all their cytotoxicity in hypoxic media. In vivo studies were performed in a subcutaneously colon carcinoma tumour model and the PDT efficiency was evaluated for TLD1829 only under both red (192 or 266 J∙cm^−2^) and NIR irradiation (600 J∙cm^−2^). Under red light illumination (266 J∙cm^−2^), a significant tumour regression was observed with a majority of complete regression. Under NIR light, similar observations could be done (Table 21)

### 5.2. O_2_ independent Cytotoxic Compounds

**Figure 54:**
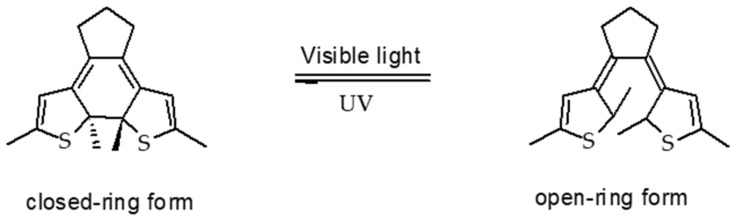
Reversible photoisomerization of the diarylethene core with UV and visible light. Adapted from Babii et al. [206].

Some other teams have developed compounds which could be photoactivated and do not need O_2_ to be cytotoxic. Babii et al. (2016) [206] used a light sensitive diarylethene-derived peptidomimetic (Figure 54), which did not need O_2_ to be cytotoxic since it was itself a toxic molecule in its isomeric form, appearing after irradiation by UV or visible light. A new photoswitchable block of peptidomimetics was designed with diarethylene connected to the peptide by a keto group. Absorption of this new compound was shifted to the red for about 50 nm.

**Figure 55:**
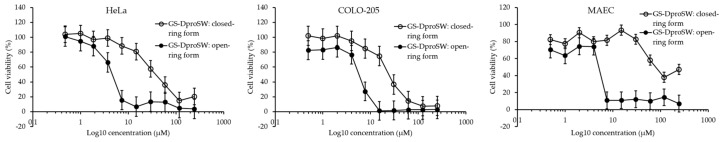
In vitro cytotoxic activities of the two GS-^DPRo^Sw photoforms as measured in the MTT test. Adapted from Babii et al. [206].

Cytotoxicity on HeLa cells and COLO-205 cells showed that the closed ring had always less toxicity (5.5 to 8-fold lower) than the open one (Figure 55). Excitation with 664 nm light led to about 50% transformation from closed-ring form to open-ring form.

In vivo, the authors used LLC model in C57B1/6 mice and PDT with visible light (100 mW∙cm^−2^, 20 min), inducing above 60 % more viability of mice after 20 days of therapy.

**Figure 56:**
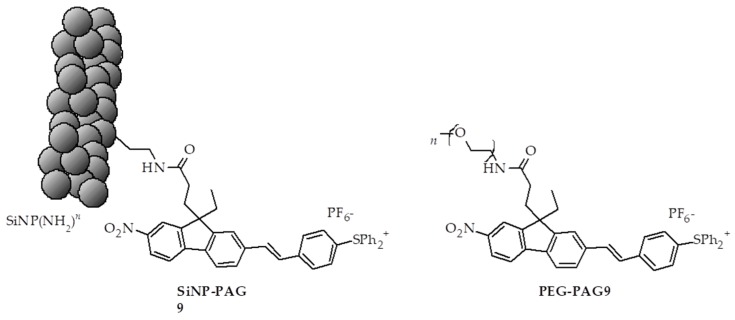
Structure of SiNP-PAG9 and PEG-PAG9. Adapted from Fadhel et al. [207].

Fadhel et al. (2016) [207,208] developed in the same work PEG-functionalized and hydrophilic silica NPs-enriched photacid generator (PAG) (Figure 56). These NPs were used to kill HCT-116 cells via an O_2_ independent mechanism, involving one or two NIR photon excitation. Under illumination, PAG incorporated in the silica NP functionalized with amines, induced the decrease of the pH inside the cells, leading to their destruction. 

Intrinsic toxicity in cells was evaluated after exposure with light at 377 nm (5.4 mW∙cm^−2^, 10 min). A viability of 64% for PEG-PAG9 and 42 % for SiNP-PAG9 was found (Table 22).

### 5.3. NO Donors

Several strategies relying on the use of NO donors have been developed to overcome the problem of hypoxia for PDT treatment. Since NO is known to inhibit cell respiration by competition with O_2_ by binding on mitochondrion, O_2_ consumption in cancer cells is inhibited and the spared O_2_ can be used for PDT. Some teams proposed an alternative to the O_2_-supply strategy i.e. an O_2_-economizer for the PDT treatment of hypoxic tumour.

Recently, Yu et al. [209], synthesized poly(D,L-lactide-co-glycolide) nanovesicles (PV) in which either sodium nitroprusside (SNP), tetraphenylporphyrin as PS or both were loaded. SNP was a precursor of nitric oxide NO in the presence of thiol compounds such as glutathione or cysteine. The thiols concentration in tumour cells being much higher than in normal cells, NO production was then higher. At the same time, the higher O_2_ contain was also evidenced by the use of HIF-1α and immunofluorescence assay. Thus, NO could be considered as O_2_-economizer in hypoxic tumours. This effect was verified in vitro by pO_2_ measures and NO release in normal or hypoxic conditions and in the dark or under irradiation (660 nm, 20 mW∙cm^−2^). As expected, hypoxic conditions led to lower cell viability than in normoxic ones after 3 min irradiation. However, prolonged irradiation time from 3 min to 10 min induced the death of about 90 % cells. The nature of oxidizing agent(s) was not clearly explicit. In vivo experiments were conducted on 4T1 tumour bearing female BALB/c mice and the first results exhibited a very low tumour growth in the group of PV-TS illuminated by NIR (660 nm, 150 mW∙cm^−2^, 5 min) and a progressive shrinkage of tumour until eradication.

**Figure 57:**
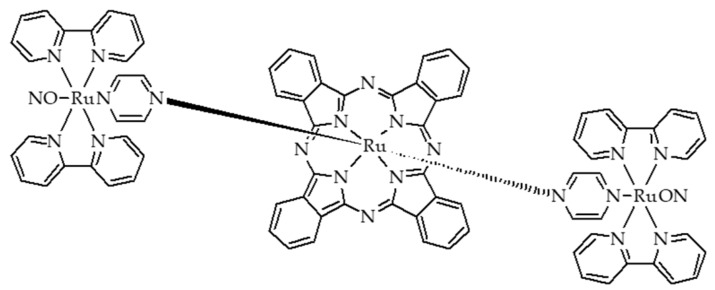
Molecular structure of [Ru(phthalocyanine)(pz)2{Ru(bpy)2NO}2]6+. Adapted from Heinrich et al. [210].

Heinrich et al. (2013) [210] studied the synergy between ROS and NO. A Ru complex (Ru(phthalocyanine)(pyrazine)_2_-(Ru(bpy)_2_(NO))_2_)(PF_6_)_6_ (Figure 57) was synthetized and produced both NO and ^1^O_2_ under 660 nm irradiation. NO production depended on excitation wavelength and pH, and reached a maximum value for 377 nm and pH3.0. In vitro, viability of B16F10 cells was found to be minimal at about 22 %, 4 h after irradiation by 660 nm laser (5 J∙cm^−2^). A proposed mechanism was the inhibition of mitochondrial respiration due to NO allowing an increased sparing of O_2_.

Wan et al. (2018) [211] also designed a NP taking benefit from the synergy between PDT and NO production. Starting from a porous coordination network (porphyrinic metal-organic framework), containing L-arginine (a NO donor), they coated it with a cancer cell membrane (4T1 cells) and obtained the so-called (L-Arg@PCN@Mem) NPs. Under NIR (640 nm and 720 nm) irradiation, L-Arg reacted with ROS) to produce great amount of NO. In vitro, 4T1 cells were incubated under normoxic and hypoxic conditions. The cell death reached up to 71 % in normoxia and 18 % in hypoxia after PDT treatment (660 nm, 30 mW∙cm^−2^, 8 min), due in part to the long lifetime of NO and its large diffusion coefficient. In vivo experiments on 4T1 tumour bearing mice showed a nearly complete ablation of tumour 14 days after the treatment (660 nm, 200 mW∙cm^−2^, 10 min).

**Figure 58:**
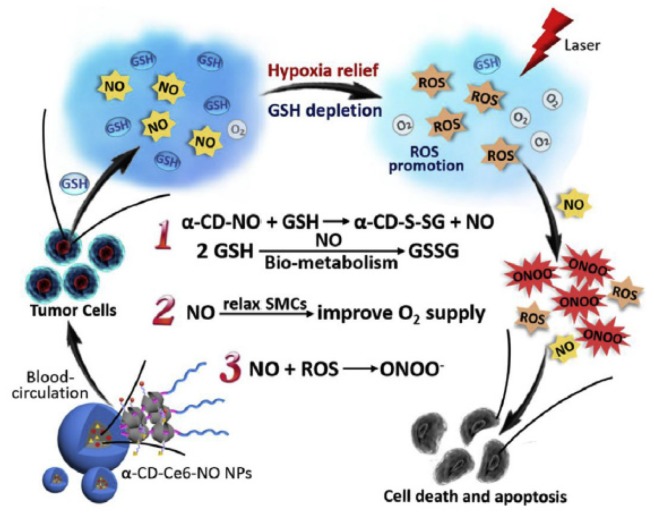
Multiple synergistic effects between NO and PDT generated from the supramolecular NPs α-CD-Ce6-NO NPs to improve therapeutic efficacy. Reprinted from [212] with permission from Elsevier Ltd, Copyright 2018.

Deng (2018) et al. [212] synthetized a NO nanogenerator, a glutathione (GSH)-sensitive NO carrier by conjugating S-nitrosothiol to alpha-cyclodextrin, integrated in a supramolecular nanocarrier alpha-CD-Ce6-NO (Figure 58). This compound presented many advantages: (1) acted as GSH scavenger, (2) SMC (smooth muscle cells) relaxation to increase blood flow and 3) NO generation which could react with ROS to produce radical nitrogen species (RNS).

**Figure 59:**
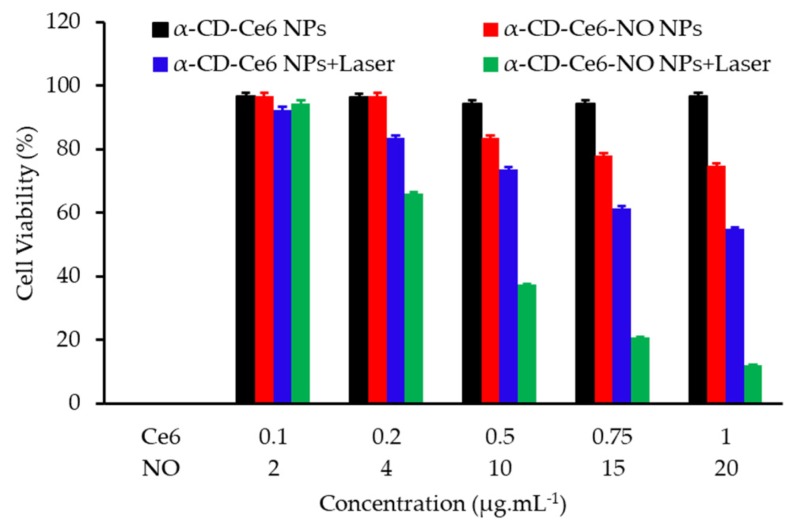
Cytotoxicity of MCF-7 cells incubated with α-CDCe6 NPs, α-CD-Ce6-NO NPs, α-CD-Ce6 NPs with laser, and α-CD-Ce6-NO NPs with laser (660 nm, 0.200 W, 2 min). Adapted from Deng et al. [212].

After NPs internalization in MCF-7 cells great amount of ROS was produced when cells were exposed to 660 nm laser irradiation without GSH, slightly less when GSH was added, but nevertheless four times larger than NP without NO. Cell viability (Figure 59) decreased up to 12.6 % when synergy between PDT (660 nm, 0.2 W∙cm^−2^, 2 min) and NO production was taken into account.

In vivo, tumour growth (nude mice bearing MCF-7 xenograft) was clearly inhibited with this treatment, going up to a complete ablation 21 days after treatment (660 nm, 0.5 W∙cm^−2^, 5 min) (Table 23).

### 5.4. O_2_ Donor

We report two teams who have described the use of compounds with endoperoxide able to directly generate ^1^O_2_ under photothermal conditions. Yuan et al. [213] used substituted diphenyl anthracene (DPA) as ^1^O_2_ donor. The endoperoxide of DPA is known to be stable and biocompatible, with a great ^1^O_2_ formation yield. Substituents in various positions affect the cycloreversion. A nanomicelle of 60 nm diameter was synthesized with an amphiphilic block copolymer in which were introduced both ortho-substituted DPA and tetraphenyl porphyrin. IR780 iodide was then encapsulated in the core of the NPs to form PMT NPs. These PMT NPs could produce heat and ROS under illumination at 808 nm, heat conduced to the release of ^1^O_2_ from endoperoxide of DPA. In vitro, PDT was performed onto to HepG2 cells (human liver carcinoma) and led to more than 95 % of dead cells, compared to about 20 % when no substituted DPA was used. In vivo experiments on HepG2 tumour bearing mice, with a light density around 1.0 W∙cm^−2^ were performed. Large tumour with a volume of 500 mm^3^ was treated both with PT NPs (block copolymer different) and PMT NPs. In the first case, growth was inhibited during treatment but accelerated after. In the second case, a decrease of the tumour volume was observed after three days of treatment (808 nm, 1 W∙cm^−2^, 2–4 min), leading to such a weak value of the volume that cannot be measured.

Han et al. [214] designed pH-responsive micelles (C/O@N-Micelle) with an average size of 43 nm and obtained by self-assembly of the triblock copolymer poly(ethyleneglycol)-β-poly (ε-capro- lactone)-β-poly(2-(piperidin-1-yl)ethyl methacrylate) (PEG-b-PCLb-PPEMA) co-encapsulating cypate (a photothermal agent) and a ^1^O_2_ donor (diphenylanthracene endoperoxide, DPAE). Under NIR irradiation (808 nm, 1.5 W∙cm^−2^, 5 min), an increase of temperature of 7 °C was highlighted confirming the photothermal effect of cypate and an efficient ^1^O_2_ generation due to this effect. In vitro in 4T1 cells, a better cellular uptake of C/O@N-Micelle was observed when the pH was decreased from 7.4 to 6.8 and a high cytotoxic effect was observed under a combined PTT/PDT treatment (808 nm, 1.5 W∙cm^−2^, 5 min) with C/O@N-Micelle. In vivo in 4T1 tumour-bearing mice, they confirmed in vitro results with high tumour accumulation, significant hyperthermia and ROS production and an efficient tumour growth inhibition after PTT/PDT treatment with C/O@N-Micelle (Table 24). 

### 5.5. Active Compounds in both Normoxic and Hypoxic Conditions 

**Figure 60:**
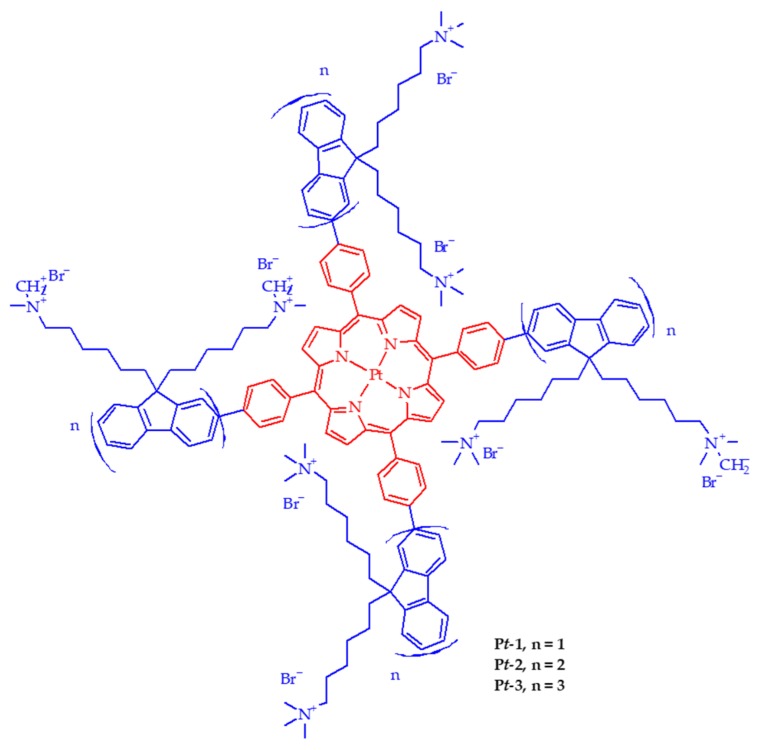
Chemical structures of Pt-1, Pt-2, and Pt-3. Adapted from Lv et al. [215].

Lv et al. [215] elaborated bifunctional agents, based on Pt (II) porphyrins, for tumour hypoxia imaging and PDT treatment efficient under hypoxia. A Pt (II) porphyrins core and four cationic fluorine oligomers arms with different lengths composed the three agents synthesized (Pt-1, Pt-2 and Pt-3) (Figure 60). 

These compounds presented emission in the red with an increase of phosphorescence emission with decreasing level of O_2_ and high ϕ_Δ_ of 0.80, 0.86 and 0.92 respectively. Pt-3 showed the lowest aggregation and the highest O_2_ sensitivity due to its 3D architecture and was tested in vitro and in vivo. In vitro studies were performed in HeLa cells incubated with Pt-3 and irradiated by light at 520 nm (10 mW∙cm^−2^, 10 min). The molecule showed high efficiency in normoxic media to kill cancer cells and under hypoxic atmosphere, its efficiency was significantly better than hematoporphyrin used for comparison, with an apparition of apoptosis of cells 1 h after PDT for Pt-3 against 2h for HP. A HeLa xenograft tumour-bearing mice model was used for in vivo studies. Under 520 nm irradiation (160 mW∙cm^−2^, 10 min), a high decrease of tumour volume was observed for Pt-3 and HP with a more significant decrease for Pt-3 (Table 25).

**Figure 61:**
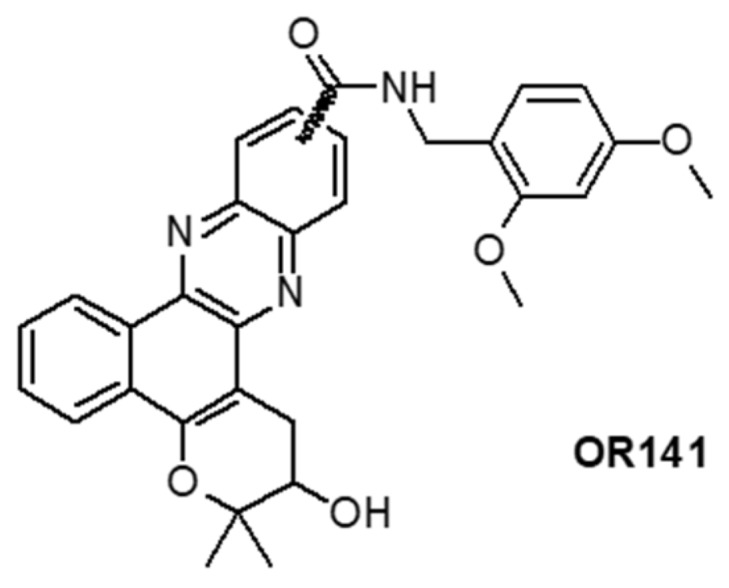
Molecular structure of OR141. Adapted from Pinto et al. [216].

Pinto et al. [216] investigated a benzophenazine compound (OR141) as a PS for PDT applications under normoxic and hypoxic conditions (Figure 61). They chose this compound after a phenotypic screening. 

Generation of ^1^O_2_ was identified as the main photoinduced mechanism by light excitation of OR141. The authors showed that the production of ^1^O_2_ was independent of the level of O_2_ (1 or 21 %) since almost no change was observed between the two conditions contrary to others PS such as verteporfin and MB. They studied in endothelial cells the pathways inhibited under hypoxia by OR141 under light irradiation and found that OR141 inhibited the HIF-2α expression and the mTOR (mammalian target of rapamycin) pathway. Finally, they investigated OR141 efficiency in vivo in a human colon carcinoma xenograft model. The PS accumulated during 4 h after injection and the mice were irradiated with white light (optic fiber, 15 min) after this time. This protocol was repeated each four days and a significant decrease of tumour growth was observed. 

**Figure 62:**
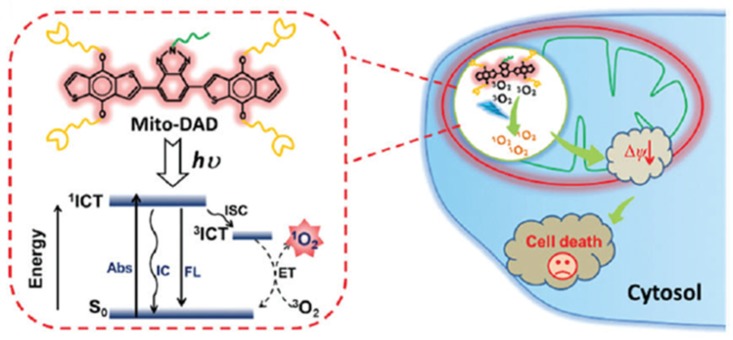
Illustration of enhanced cytotoxicity through photogeneration of ^1^O_2_ by a mitochondria-targeting DAD molecule. Republished from [217] with permission of the Royal Society of Chemistry, Copyright 2018.

Sun et al. [217] developed a novel PS based on a donor-acceptor-donor (DAD) model for targeting mitochondria and be an effective PDT agent. This molecule (Mito-DAD) was composed of an electron-donor (benzodithiophene), a median electron acceptor (benzotriazole), an oligoethylene glycol for a better water solubility, and a mitochondrial targeting unit (four triphenylphosphonium units) (Figure 62). 

This compound presented a high ϕ_Δ_ of 0.64 in methanol. Under low O_2_ concentrations (5%), the ϕ_Δ_ remained high with a value of 0.52. No ROS generation was observed in the dark but under LED light irradiation an elevation of intracellular ROS was showed. Moreover, the cell death was induced very quickly after 2 min of visible light irradiation (470 nm, 16 mW∙cm^−2^). After light irradiation (LED lamp, 3 min), the HeLa cells viability have been shown to be Mito-DAD concentration-dependent with a decrease of viability of 96% to 4.6% with concentration from 0 to 5 µM whereas under the dark, the cell viability remained superior at 90%.

Under 5% O_2_ concentration, an efficient PDT effect was also observed, after treatment of cells with Mito-DAD and irradiated (LED lamp 470 nm, 16 mW∙cm^−2^) during 5 min, with a percentage of cells viability of only 6.6% (Table 25). 

**Figure 63:**
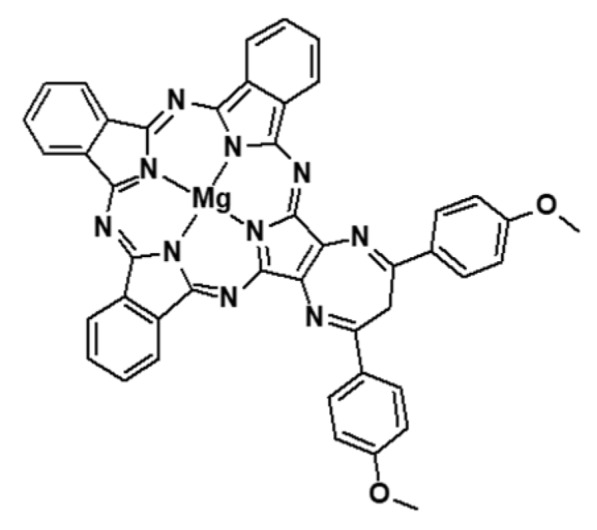
Structure of tribenzoporphyrazine. Adapted from Wieczorek et al. [218].

Wieczorek et al. [218] designed novels porphyrazines incorporated in liposomes for PDT application in both normoxia and hypoxia media. They synthesized three compounds derived from porphyrazines, one of them the tribenzoporphyrazine (Figure 63) presented a ϕ_Δ_ of 0.069 and 0.180 in DMF and DMSO, respectively. 

Two types of liposomes formulations were chosen to incorporate the porphyrazines, the first one, composed of 1-α-phosphatidyl-D,L-glycerol (PG) and 1-palmitoyl-2-oleoyl-sn- glycero-3-phosphocholine (POPC), negatively charged and the second one, composed of 1,2-dioleoyl-3-trimethylammonium-propane (chloride salt, DOTAP) and POPC, positively charged. Porphyrazines alone and their liposomes formulations were tested in vitro in human prostate carcinoma cells (LNCaP). Under normoxic conditions, the tribenzoporphyrazine, as for the DOTAP-POPC and PG-POPC formulations, showed a significant cytotoxicity under light irradiation (690 nm, 2 J∙cm^−2^). IC_50_ was significantly lower for the both liposome formulations in comparison with tribenzoporphyrazine alone. The same results were observed in hypoxic conditions (1% of O_2_).

## 6. Hypoxia-Dependent PDT 

The cell survival and propagation of the tumour are greatly facilitated by a hypoxic TME which is a common factor in tumours. Hypoxia induces a number of complex intracellular signaling pathways, a major one being the HIF pathway. The overexpression of HIF-1α and HIF-2α subunits lead to key cellular responses, which cause, among other things, the increase of blood vessel formation, aggressiveness and metastasis. 

The use of hypoxia-responsive drug delivery system for PDT seems to be a rising approach for an improvement of its efficiency, which could be alterated by hypoxia. Therefore, several strategies are designed to overcome hypoxia in the treatment of solid cancers such as the development of anti-hypoxia agents, hypoxia-active nanoparticles, and hypoxia-targeting agents for anticancer PDT. The aim of these strategies is to produce O_2_ and activate the nanoparticles or agents in the TME, but also to target biomarkers of tumour hypoxia to improve the PSs’ effectiveness that are administered. 

### 6.1. Hypoxia-Reducible Compounds

#### 6.1.1. Azobenzene (AZO)

The AZO group (−N=N−) can be reduced and cleaved by azoreductase (a typical biomarker of hypoxia) under the hypoxic environment of tumour cells, acts as the hypoxia responsive linker component.

Zhang et al. [219] reported a NPs (CPs-CPT-Ce6 NPs) composed of a conjugated polymer containing AZO bridges (CPs), which was hypoxia-responsive, adsorbing Ce6 and camptothecin as chemodrug and coating to PVP. Under irradiation (670 nm, 50 mW∙cm^−2^, 10 min), an efficient ROS production by CPs-CPT-Ce6 NPs was observed which induced an enhancement of tumour hypoxia and thus induced the dissociation of NPs by the reductive cleavage of AZO bridges and finally release CPT for an efficient chemo-PDT treatment. Both in vitro in HeLa and NIH3T3 cells and in vivo chemo-PDT (670 nm, 50 mW∙cm^−2^, 10 min) studies in HeLa tumour-bearing mice proved the better efficiency of this synergistic treatment in comparison with PDT and chemotherapy alone. 

Huang et al. [220] also developed a drug delivery system (DDS) based on the use of hypoxia-induced cleaved AZO bridges composed of gold NPs (AuNPs) functionalized with β-cyclodextrins conjugating on the surface DRHC (double-stranded DNA/RNA hybridization complex) containing HIF-1α-against antisense oligonucleotide (ASO). A PS (5,10,15,20-tetra- kis-(1-methyl-4-pyridyl)-21*H*,23*H*-porphine, TMPyP4,) was finally loaded on DDS to obtain DDS@TMPyP4. In HepG2 cells and in hypoxic conditions, an effective cleavage of AZO bridges was observed conducting to the release of ASO and a decrease of HIF-1α expression. In vitro in HepG2 and L02 cells incubated with DDS@TMPyP4, an efficient synergistic effect of hypoxia-triggered ASO release and PDT was observed (660 nm, 1 W∙cm^−2^, 30 min).

Piao et al. [221] developed azoSeR based on a seleno-rosamine-based dye (SeR) linked to an AZO. The ϕ_Δ_ was very weak for azoSeR (0.03) in comparison with SeR (0.56) due to the presence of the azo moiety which interfere with the intersystem crossing. In vitro studies were performed in rat liver microsomes to evaluate the ability of NADPH to reduce the AZO in normoxic and hypoxic conditions. Under normoxia, no change on the absorption spectra of azoSeR was observed after addition of NADPH while under hypoxia a new absorbance peak was observed. Its ability to induce cell death under light irradiation (535 nm, 28 mW∙cm^−2^, 3 min) was evaluated in human lung cancer-derived A549 cells incubated with the PS under normoxia or hypoxia conditions. The cell death was mainly induced for cells treated under hypoxia. Moreover, azoSeR was able to selectively kill hypoxic cells without ablating normoxic cells. The evaluation of sensitivity of azoSeR to different O_2_ concentrations, under light irradiation, showed a significant toxicity even for O_2_ concentration of 5% and the authors also observed an increase of cell death in mild hypoxia conditions (~ 8% of O_2_). 

Li et al. [222] developed polymeric micelles which presented the ability to be responsive to both hypoxia and ^1^O_2_ to improve the tumour uptake and the cargo release of the PS in the tumour site. The micelles (mPEG-Azo-PAsp-IM Micelles) were composed by AZO covalently bind to both hydrophilic (mPEG) and hydrophobic (PAsp-IM) polymers, the last one containing the ^1^O_2_-responsive moiety (imidazole, IM). The micelles were formed by self-assembly of mPEG-Azo-PAsp-IM and could encapsulated Ce6 with a content of 4.1±0.5%. The ^1^O_2_ production could induce a rapid drug release whereas the hypoxia conditions could induce a dissection of AZOto enhance the PS uptake in tumour. The micelles without AZO (SR micelles) were synthesized as a comparative to mPEG-Azo-PAsp-IM micelles (DR micelles) for investigation of Ce6 release. The authors showed similar ^1^O_2_ production for both SR and DR micelles under a 660 nm irradiation (100 mW∙cm^−2^, 10 min) and observed a good Ce6 release due to the dissociation of micelles by oxidation of imidazole moiety by ^1^O_2_. In Lewis lung carcinoma (LLC) cells, the intracellular level of Ce6 was significantly higher after laser irradiation of DR micelles in hypoxic conditions in comparison with SR micelles. In vitro PDT studies in Lewis lung carcinoma (LLC) cells were performed to investigate the efficiency of DR micelles in comparison with Ce6 alone and SR micelles. High similar phototoxicity (irradiation at 660 nm, 100 mW∙cm^−2^, 10 min) was observed in hypoxic conditions for Ce6 alone and DR micelles, which 2 folds more than that for SR micelles. Under normoxic conditions, Ce6 alone showed the highest photocytotoxicity and for DR micelles, it was lower than that in hypoxia. The in vivo PDT tests in LLC tumour-bearing mice highlighted a better efficiency of DR micelles in comparison with SR micelles confirming the in vitro studies.

**Figure 64:**
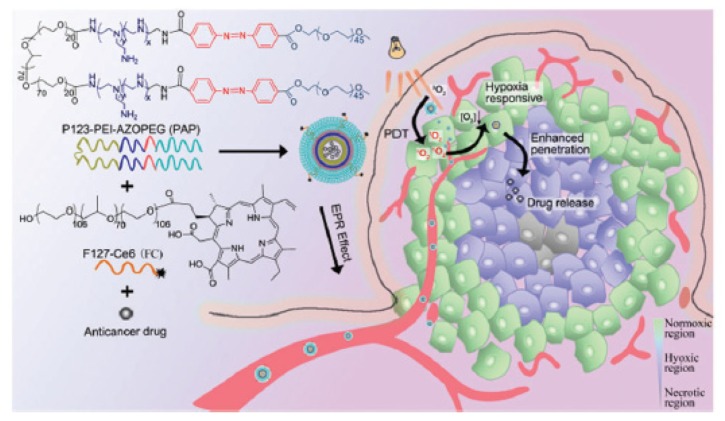
The structure and mechanism of PAP-FC for combined chemo-PDT. Republished from [223] with permission of the Royal Society of Chemistry, Copyright 2018.

Li et al. [223] designed a light-enhanced hypoxia responsive NP denoted (PAP-FC NPs) for synergic treatment of solid tumours. The PAP molecules were obtained by covalent combination of poly(ethylene oxide)-block-poly(propylene oxide)-block-poly(ethylene oxide) triblock copolymer Pluronic (P123), polyethyleneimine (PEI600), hypoxia-responsive AZO, and polyethylene glycol (PEG2000), while FC molecules were obtained by covalent conjugation of Ce6 to triblock copolymer Pluronic F127. At critical micellar concentration, PAP and FC polymers self-assembled into PAP-FC NPs, which ensure the encapsulation of DOX and thus afford PAP-FC/DOX for combined chemo-PDT treatment (Figure 64).

According to both the in vitro and in vivo studies against MCF-7 cells, PAP-FC/DOX found to be able to generate high level of ^1^O_2_ upon laser irradiation (660 nm, 8 mW∙cm^−2^, 6 min). In addition, followed by consumption of the tissue O_2_, the AZO bond broke quickly in the hypoxia condition, leading to an efficient release of DOX, and therefore triggering apoptosis of the internal tumour cells, which resulted in enhanced antitumour efficacy. 

Wang et al. [224] designed a kind of “one stone two birds” living drug delivery system by combining the PDT and hypoxia responsive chemotherapy, denoted as Ce6-PEG-Azo-PCL for cervical cancer treatment. This living drug delivery system was fabricated with three essentials constituents, i.e., AZO, biodegradable hydrophobic poly(ε-caprolactone) (PCL), and hydrophilic poly(ethylene glycol) (PEG)-conjugated Ce6. Subsequently, the functional NP Ce6-PEG-Azo-PCL was further loaded with DOX to afford Ce6-PEG-Azo-PCL@DOX (DOX@NP). Irradiation of DOX@NP with 671 nm laser (10 mW, 5 min) resulted in high ROS generation. The continuous consumption of O_2_ facilitated intracellular hypoxia microenvironment, and triggered the disassembly of hypoxia responsive AZO at tumour site to release loaded DOX resulting in enhanced anticancer effect. 

#### 6.1.2. Other

**Figure 65:**
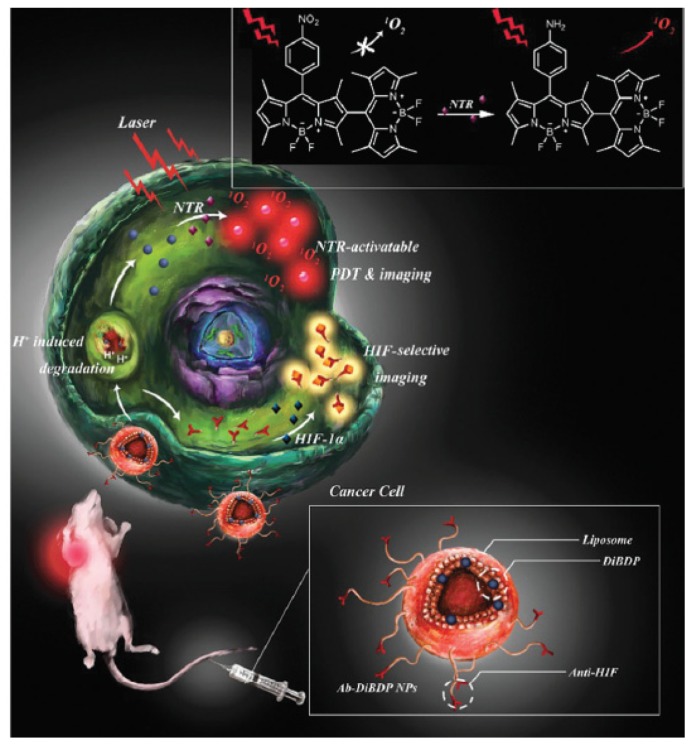
Schematic illustration of Ab-DiBDP NPs for dual hypoxia marker imaging and activatable PDT against tumours. Republished from [225] with permission of the Royal Society of Chemistry, Copyright 2018.

Chen et al. [225] designed pH-sensitive nanoliposomes constituted by a PS activatable under hypoxia conditions (DiBDP), substituted by a nitro group, and a Cy7-marked anti-HIF-1α antibody (Ab-DiBDP) for theranostic applications. DiBDP was not able to release ^1^O_2_ due to the nitro group but could be reduced by an enzyme, the nitroreductase (NTR), and thus, was “switched on” and could release ^1^O_2_ (Figure 65). The nanoliposomes showed an average size of 86±17 nm and were able to release quickly 86 % of DiBDP at pH 5.0 within 24 h. 

They first evaluated the NTR-activatable generation of ^1^O_2_ induced by the irradiation of DiBDP by using DPBF and showed a high increase of ϕ_Δ_ of the PS, from 0.05 to 0.46 when it was incubated with NTR. They performed the same evaluation for Ab-DIBDP NPs, by using the SOSG and showed a significant increase of SOSG fluorescence with NTR. The combination of Ab-DiDBP NPs and laser in vitro in HeLa cells in hypoxic conditions showed a high cytotoxicity (more than 56 % of cell death). In vivo studies in HeLa tumour-bearing mice were also performed to evaluate the efficiency of Ab-DiBDP NP-mediated PDT. The tumour growth was severely reduced for mice treated with Ab-DiBDP NPs and laser in comparison with other treatments (Ab-NPs + laser and DiBDP + laser) (Table 26).

### 6.2. Environment-Accumulated Hypoxia Compounds 

Evans et al. [226] described the use of a MB like molecule (EtNBS) capable of destroying hypoxic region in the core of 3D models of OVCAR-5 human OvCa cells of metastatic ovarian cancer. EtNBS was capable to incorporate into the core of the nodule. After PDT (652 nm, 15 J∙cm^−2^, 25 to 300 mW∙cm^−2^) even at low light fluence (5 J∙cm^−2^) EtNBS could destroy the nodule core cells. At higher fluence, the entire nodule could be destructed. In hypoxic conditions, PDT (670 nm, 100 mW∙cm^−2^, 5 to 20 J∙cm^−2^) with EtNBS was still very efficient and the authors could observe significant cell killing at 20 J∙cm^−2^. In vivo experiments will be performed.

Guan et al. [227] synthesized a novel theranostic nanosheet, composed of a metal complex (Ru(C-bpy)_2_) loaded to single layered layered double hydroxide (LDH), denoted as Ru(C-bpy)_2_/mLDH) for theranostic applications. The nanosheets Ru(C-bpy)_2_/mLDH showed an average diameter of 35±5 nm and a stronger fluorescence under an excitation at 488 nm for hypoxia media in comparison with normoxic media. Under light excitation (520 nm, 100 mW∙cm^−2^ for 8 min), a significant decrease of cell viability was observed for Ru(C-bpy)_2_/mLDH which appeared to be more phototoxic than Ru(C-bpy)_2_ alone. They observed that ϕ_Δ_ was depended of the excitation wavelength and the combination of single layered LDH with the ruthenium complex significantly enhanced the generation of ^1^O_2_ with a ϕ_Δ_ of 0.28 for Ru(C-bpy)_2_/mLDH against 0.19 for Ru(C-bpy)_2_ alone. An in vivo model of nude mice bearing subcutaneous HeLa tumour was used to evaluate the PDT efficiency of the nanosheets. The animals treated with Ru(C-bpy)_2_/mLDH and irradiation at 520 nm (100 mW∙cm^−2^) for 8 min, showed a significant suppression of tumour volume in comparison with the control and Ru(C-bpy)_2_ groups.

### 6.3. Other

**Figure 66:**
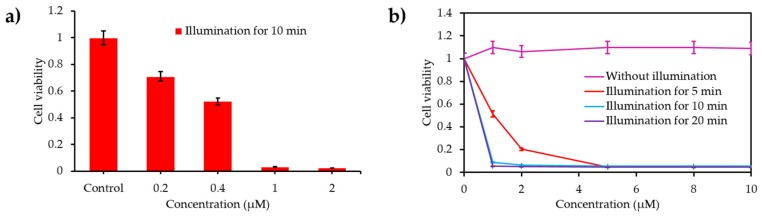
(**a**) Cell viability of A549 cells incubated with PC70 at gradient concentrations for 3 h and exposed to light irradiation for 10 min at a power density of 17 mW∙cm^−2^ and (**b**) dose- and time-dependent PDT effects of PC70 on the A549 cell viability. Adapted from Guan et al. [228].

Guan M. et al. [228] developed a biocompatible, water soluble and photostable PS, the trismethylpyridylporphyrin-C70 (PC70) constituted by C_70_ linked to 5-(4-formyl- phenyl)-10,15,20-tris(4-pyridyl)-porphine (D-TMPyP), which could be used in hypoxic media. PC_70_ could be assembled in a structure similar to liposome with a diameter of 30 nm. In vitro studies in A549 cells were performed to evaluate PDT efficiency of this compound in normoxic and hypoxic conditions. Under normoxic conditions, it was shown that PC_70_ phototoxicity was both concentration and illumination time-dependent. For cells incubated with 1 µM of PC_70_ and illuminated with white light (17 mW∙cm^−2^) during 10 min, the percentage of cell death reached 98 % (Figure 66). 

Under hypoxic conditions, in the same conditions of irradiation as previously, the photodamage of A459 cells was significantly higher with PC_70_ (80%) in comparison with D-TMPyP (22 %) demonstrating the high potential of PC_70_ under white light illumination, to kill hypoxic cells (Table 27). 

## 7. Fractional PDT

The strategy is to intermittently irradiate the tumour in order to regulate the consumption of ^3^O_2_ and the formation of ^1^O_2_. This discontinuous irradiation technique proved positive in most cases studied. This phenomenon seems due to the fact that the pulsed irradiation allows an optimal replenishment of cellular O_2_ (Table 28).

In 1996, Van Geel et al. [229] investigated the influence of different ways of illumination (single or fractionated) or different fluence rates for a discontinuous illumination, on the PDT efficiency of two PS (Photofrin and *meta*-tetrahydroxyphenylchlorin, mTHPC) in female C3H/Km mice inoculated with RIF1 cells. They first studied the difference between interstitial and superficial illumination and showed that to have the same fluence rate of interstitial illumination they have to increase the time of superficial illumination, by a factor of 2.7. However, they did not observe a significant difference of tumour response with PDT treatment Photofrin for both illuminations whereas they highlighted an increased response with mTHPC under superficial illumination. The fractionated illumination studies were performed only with mTHPC and no marked difference on tumour regrowth was observed between continuous or fractionated illumination. They also tested a single dose application of mTHPC in comparison with two doses (each 24h before the illumination) and observed no difference in the tumour response. However, for a single dose of mTHPC and by varying the time interval between each illumination, even if no change was observed on the delay for tumour regrowth, an increase of cure mice was observed for a time interval of 1h. Moreover, the number of cure mice was better with the injection of two doses of mTHPC. They finally determined that the fluence rate, for a discontinuous illumination had no influence on the tumour response.

In two studies, Klimenko et al. [230,231] studied the influence of the type of irradiation (pulse of continuous) on the O_2_ consumption and ^1^O_2_ generation. A first theoretical model showed a high decrease of O_2_ generation and the cumulative ^1^O_2_ production at high fluence rates under continuous wave (CW) mode whereas, for pulse irradiation mode, a high level of ^3^O_2_ was maintained as the cumulative ^1^O_2_ concentration, which increased in comparison with CW mode. 

Both modes of irradiation were tested in vitro in k562 cell line by using radachlorin and the authors observed that there was less cell surviving while CW mode in comparison with pulse mode at fluence of 1.25 and 2.5 J∙cm^−2^. Moreover, they observed different mechanisms of cell death with the two modes with an apoptotic mechanism for pulse mode and a necrotic mechanism for CW.

Kawauchi et al. [232] in 2004 studied the difference in mouse renal carcinoma cells (Renca) viability and total O_2_ consumption and O_2_ consumption rate after pulsed laser irradiation (670 nm nanosecond pulsed Nd:YAG laser, peak fluence rate 1 mW∙cm^−2^, 30 Hz) or continuous laser irradiation (CW, 670 nm, 40 J∙cm^−2^) by using (13,17-bis[1-carboxypropionyl]carbamoyl- ethyl-3-ethenyl-8-ethoxyiminoethylidene-7-hydroxy-2,7,12,18-tetramethylporphyrin sodium (PAD-S31)/ Concerning the cytotoxicity at two different irradiances (180 mW∙cm^−2^ and 270 mW∙cm^−2^), continuous excitation induced more cell viability than pulsed irradiation. The cytotoxic effect was independent of the fluence rate for both excitation modes. The O_2_ consumption rate decreased of half after pulsed light compared to CW. The total O_2_ consumption was also less important with pulsed light compared to CW. Fluence rates for both excitation modes influenced the O_2_ consumption (less O_2_ consumption for 270 mW∙cm^−2^ than for 180 mW∙cm^−2^) but did not have any influence on the total O_2_ consumed.

**Figure 67:**
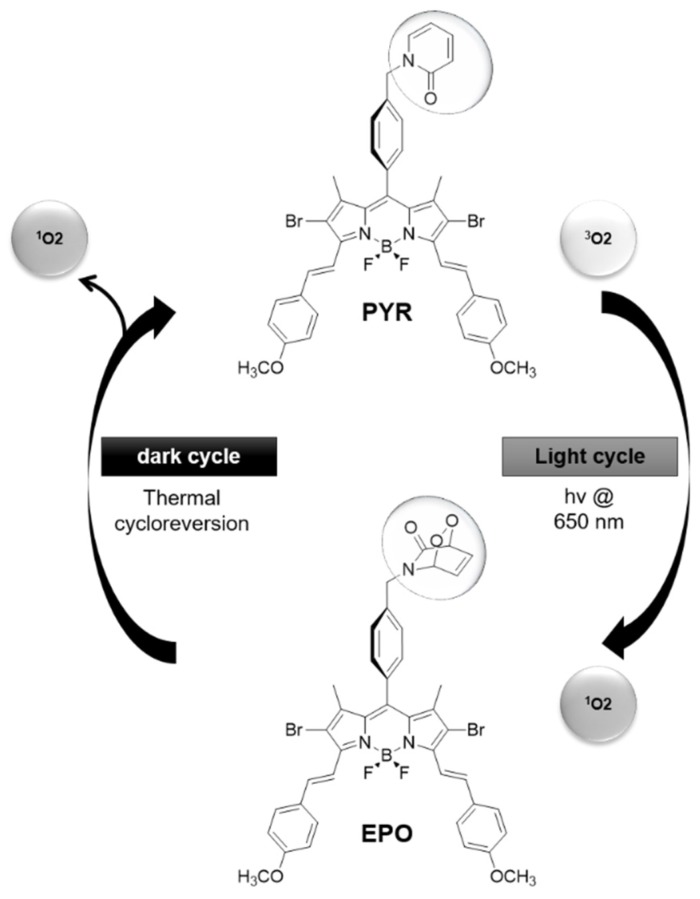
^1^O_2_ generation achieved first by irradiation of bifunctional compound PYR at λ = 650 nm, and subsequently by thermal cycloreversion in the dark. The cycles can be repeated indefinitely. Adapted from Turan et al. [233].

In 2016, Turan et al. [233] developed a PS for fractional PDT able to produce ^1^O_2_ under light irradiation and in the dark. This PS was based on a pyridone (PYR) which could be converted in 2-pyridone endoperoxide (EPO) after light irradiation (650 nm) by reaction with ^1^O_2_ generated. In the dark EPO was able to release ^1^O_2_ by thermal conversion and thus returned to the PYR form (Figure 67). 

Under alternation of dark and light (650 nm, 15 min) cycles on this PS, a continuous production of ^1^O_2_ was observed. The PDT efficiency of PYR and EPO was evaluated in vitro in HeLa cells and for a better solubility the compound was incorporated into micelles. After administration of the compounds and 655 nm LED array irradiation (324 µmol∙m^−2^∙s^−1^ photon flux) during 10 min repeated every one hour, a significant decrease of cell viability was observed for both compounds (Table 28).

Moreover, EPO seemed to present a better phototoxic effect with a CC50 (50% cytotoxic concentration) of 8.6 nM against 49.0 nM for PYR.

## 8. Conclusions

The first clinical trials in PDT were performed in 1978 by Dougherty in Roswell Park (Buffalo, NY, USA) and the first approval of PDT using Photofrin for the treatment of bladder cancer was 25 years ago in 1993. Since then, many efforts have been done to improve the chemistry of the PS, the light illumination and also the oxygenation of the tissue. In this review, we collected all the data till March 2019 concerning the improvement of oxygenation for PDT applications. We showed that different strategies can be used to decrease hypoxia, leading to highly efficient treatment, and even metastasis inhibition of aggressive hypoxic malignant cancers. In all the studies, two main approaches have been described concerning hypoxia. In one side, the authors tried to relieved tumor hypoxia by carrying O_2_ in tumour or decreasing tumour O_2_ comsumption and in another side, they used hypoxia to activate their designed platforms and thus improved PDT efficiency. To summarize, five different ways can be followed: (1) playing with the light and realizing fractionated illumination to allow the re-oxygenation of the tissues between two illuminations; (2) designing molecules or NPs that are able to produce ROS even in hypoxic media; (3) adding O_2_ into the hypoxic media by transporting O_2_ with hemoglobin or perflu orocarbon systems, improving the blood flow by increasing the temperature or with combination of anti-angiogenic compounds, producing O_2_ by the decomposition oh H_2_O_2_ in the hypoxic medium; (4) using combined strategies, in particular hypoxic-activated chemotherapy, PTT that does not require O_2_ or antiangiogenic therapy and (5) development of hypoxia-activated compunds. These strategies present a significant interest to enhanced cancer-PDT effect by fighting hypoxia. Many in vitro and in vivo experiments in all kind of cancer proved that the decrease of hypoxia really improved PDT efficiency. At this time, no clinical trial is registered using one of this strategy, it should be the next step.

Overcoming hypoxia to improve PDT is a relatively new subject whose interest has been steadily increased since the 1990s. In 1991, Freitas and Baronzio [5] reported a few strategies to fight hypoxia by improving the oxygenation of tumours which rely essentially on the increase of tumour O_2_ concentration. In our review, we reported an enhancement of the attention for hypoxia these last years with an increase of publications about hypoxia and PDT year after year. In our point of view, fighting hypoxia could become essential for an efficient PDT treatment of cancer and could lead to significant improvements for the treatment of patients.

## Figures and Tables

**Table 1:** % Mammary carcinoma tumour implanted into C3H mice after PDT treatment (630 nm, total light irradiation of 150 mW∙cm^−2^).

	0 h	4 h	18 h	24 h
RA	0.495	0.381	0.043	0.0045
NBO	0.185	0.120	0.006	0.00035

**Table 2:** Summary of publications about HBO as O_2_ vehicle.

Ref	Application	O_2_ Source	PS	Energy of Excitation	Type of ROS	In Vitro	In Vivo
[20]	PDT	Hyperbaric chamber with 100% O_2_ (21, 100, 200, 300, or 400 kPa)	5-ALA	435 nm, 12.9 mW∙cm^−2^, 0.5 min and 30 min	^1^O_2_	AY27, WiDr and SW840 cell line	No
[21]	PDT	HBO (0.25 MPa; 98% O_2_ + 2% CO_2_)	5-ALA	630 nm, 5 J∙cm^−2^, 8 h per days	ROS	A431 cancer cell line	No
[22,23,24,25]	PDT	HBO or NBO 100% O_2_ (hyperbaric chamber: 3 atp)	Photofrin	630 nm, “Fractional light” group: 150 mW∙cm^−2^, 200 J∙cm^−2^, 30 s light and dark intervals “Reduced light dose rate” group: 75 mW∙cm^−2^, 200 J∙cm^−2^	^1^O_2_	No	MCa tumour-bearing mice
[26]	PDT	HBO (hyperbaric chamber: 2.5 atm)	RB	808 nm 16 min, 0.75 W∙cm^−2^	ROS	4T1 cell line	4T1 tumour bearing mice
[27,28,29,30,31,32,33]	PDT	HBO (hyperbaric chamber: 2 atm O_2_)	HpD (Photosan) or 5-ALA	630 nm, 300 J∙cm^−2^	nd	No	Clinical study on patients with oesophageal carcinoma
[34]	PDT	Nicotinamide and carbogen (95% oxygen with 5% carbodioxide)	*m*-THPC	652 nm, 20, 100 and 200 mW∙cm^−2^, 5 min	nd	No	H-MESO1 tumour-bearing mice

nd: not determined; HBO: hyperbaric oxygen; 5-ALA: 5-aminolevulinic acid; RB: Rose Bengal; HpD: haematoporphyrin derivatives; *m*-THPC: *meta*-tetra(hydroxyphenyl)chlorin.

**Table 3:** Summary of publications about RBC as O_2_ vehicle.

Ref	Application	Vehicle	PS	Energy of Excitation	Type of ROS	In Vitro	In Vivo
[36]	PDT	Artificial red cells (ARCs) containing Hb	IGC	In vitro: 808 nm, 100 mW∙cm^−2^, 5 min In vivo: 808 nm, 100 mW∙cm^−2^, 30 min	ROS	MCF-7 cell line	MCF-7 tumour-bearing mice
[37]	PDT	Hb	Ce6	In vitro: 660 nm, 0.1 W∙cm^−2^, 2 min In vivo: 660 nm, 0.1 W∙cm^−2^, 30 min	^1^O_2_	4T1 cell line	4T1 tumour-bearing mice
[38]	PDT	RBCs	ZnF_16_Pc	In vitro: 671 nm, 100 mW∙cm^−2^, 0–60 min In vivo: 671 nm, 100 mW∙cm^−2^, 30 min	^1^O_2_	U87MG cell line	U87MG tumour bearing mice
[39]	PDT	RBCs	RB	980 nm + 808 nm, 1.5 W∙cm^−2^, 15 min	^1^O_2_	U87MG cell line	U87MG tumour-bearing mice
[40]	PDT	Hb	RB	In vitro: two-photon 808 nm, 390 mW∙cm^−2^, 20 min In vivo: two-photon 808 nm, 390 or 250 mW∙cm^−2^, 8 or 15 min	^1^O_2_	MCF-7 cell line	MCF-7 tumour-bearing mice
[41]	PDT	Hb	ICG	808 nm, 1 W∙cm^−2^, 1min	^1^O_2_	CT-26 cell line	S180 and CT26 tumour-bearing mice
[42]	PDT	Hb	BODIPY-Br_2_	635 nm, 25 mW∙cm^−2^, 10 min	^1^O_2_	HepG2 cell line	No
[43]	PDT	Hb	TCPP	600 nm, 70 mW∙cm^−2^, 2min	^1^O_2_	4T1 cell line	No
[44]	PDT	RBC membranes encapsulating Hb	MB	In vitro: 660 nm, 30 mW∙cm^−2^, 5 min In vivo: 660 nm, 220 mW∙cm^−2^, 5 min	^1^O_2_	4T1 cell line	4T1 tumour-bearing mice
[45]	PDT	Hb-RBC	ICG	808 nm 60 mW∙cm^−2^ 5 min	^1^O_2_	Macrophages (RAW264.7 cell line) + multicellular tumour spheroids	MCF-7 tumour bearing mice

Hb: Hemoglobin; ICG: Indocyanine green; Ce6: Chlorin e6; RBC: Red Blood cells; ZnF_16_Pc: zinc 1,2,3,4,8,9,10,11,15,16,17,18,22,23,24,25-hexadecafluoro-29*H*,31*H*-phthalocyanine; RB: Rose Bengal; BODIPY-Br_2_: 4,4-difluoro-4-bora-3a,a-diaza-s-indacene brominated; TCPP: carboxyphenyl-porphyrin; MB: methylene blue.

**Table 4:** Summary of publications about PFC as O_2_ vehicle.

Ref	Application	O_2_ Source	PS	Energy of Excitation	Type of ROS	In Vitro	In Vivo
[48]	PDT	PFH	IR780	808 nm, 2 W∙cm^−2^, 20 s	^1^O_2_	MCF-7 and CT26 adenocarcinoma cell lines	CT26 tumour-bearing mice
[49]	PDT	PFC	Fluorous porphyrin	420 nm, 8.5 mW∙cm^−2^, 30 min	^1^O_2_	A375 cell line	No
[50]	PDT	PFH	Plasmon resonance	670 nm, 0.48 mW∙cm^−2^, 10 min	^1^O_2_	MCF-7 cell line	MCF-7 tumour-bearing mice
[51]	PDT	Pentafluorophenyl	5,10,15,20-tetrakis(4-aminophenyl) porphyrin	In vitro: 655 nm, 1.52 mW∙cm^−2^, 15 min In vivo: 655 nm, 1.52 mW∙cm^−2^, 60 min	^1^O_2_	SMMC-7721 cell line	No
[52]	PDT	PFOB	ICG	In vitro: 808 nm, 1 W∙cm^−2^, 3 min In vivo: 808 nm, 1 W∙cm^−2^, 10 min	^1^O_2_ + ROS	MDA-MB-231 cell line	MDA-MB-231 tumour-bearing mice
[53]	Ultrasound triggered PDT and RT	PFC	Ce6	671 nm, 1.2 W∙cm^−2^, 5 min	^1^O_2_	4T1 and CT26 cell line	4T1 and CT26 tumour-bearing mice
[54]	PDT	PFC	ZnF_16_Pc	In vitro: 671 nm, 100 mW∙cm^−2^, 200 s In vivo: 671 nm, 500 mW∙cm^−2^, 10 min	^1^O_2_	U87MG cell line	U87MG tumour-bearing mice
[55]	PDT	PFH	IR780	808 nm, 2 W∙cm^−2^, 20 s	^1^O_2_	CT26 cell line	CT26 tumour-bearing mice
[56]	PDT	PFSEA	THPP	In vitro: 660 nm, 10 mW∙cm^−2^, 30 min In vivo: 660 nm, 50 mW∙cm^−2^, 60 min	^1^O_2_	4T1 cell line	4T1-tumour-bearing mice
[57]	PDT + PTT	PFH	AlPc	670 nm, 1 W∙cm^−2^, 5 min	^1^O_2_	4T1 cell line	4T1-tumour-bearing mice
[58]	PDT	Perfluorooctanoic acid	Ce6	In vitro: 630 nm, 30 mW∙cm^−2^, 3 min In vivo: 630 nm, 500 mW∙cm^−2^, 20 min	^1^O_2_	C6, HepG2 and HeLa cell lines	C6 glioma tumour-bearing mice
[59]	PDT	PFH	Ce6	670 nm, 1 W∙cm^−2^, 1 min	^1^O_2_	4T1 cell line	4T1 tumour-bearing mice
[60]	PDT	Fluorinated polypeptide NPs	BODIPY-Br_2_	In vitro: 635 nm, 34 mW∙cm^−2^, 10 min In vivo: 635 nm, 100 mW∙cm^−2^, 10 min	^1^O_2_	HepG2 cell line	4T1 tumour-bearing mice
[61]	PDT + PTT	Perfluorooctyl bromide	IR780	In vitro: 808 nm, 1 W∙cm^−2^, 5 min In vivo: 808 nm, 1 W∙cm^−2^, on 30 s, off to room temperature, 20 cycles	^1^O_2_	4T1 cell line	4T1 tumour-bearing mice
[62]	PDT	Perfluorooctyl bromide	IR780	808 nm, 2 W∙cm^−2^, 20 s	^1^O_2_	MDA-MB-231 and MCF-7 cell lines	MDA-MB-231 tumour-bearing mice
[63]	PDT	PFH	Ce6	In vitro: 660 nm, 100 mW∙cm^−2^, 5 min In vivo: 660 nm, 100 mW∙cm^−2^, 10 min	^1^O_2_ + ROS	MDA-MB-231 cell line	MDA-MB-231 tumour-bearing mice
[64]	PDT	PFC	Hypocrellin B	630 nm, 20 mW∙cm^−2^, 300 s, 6 J per well	^1^O_2_	H1299 cell line	No
[65]	PDT	NPs with fluorocarbon chains	IR780	In vitro: 808 nm, 2 W∙cm^−2^, 20 s In vivo: 808 nm, 2 W∙cm^−2^, 5 min	^1^O_2_	4T1 tumour spheroids	4T1 tumour-bearing mice

PFH: perfluorohexane; PFC: perfluorocarbon; PFOBRB: perfluorooctyl bromide; ICG: Indocyanine green; Ce6: Chlorin e6; ZnF_16_Pc: zinc 1,2,3,4,8,9,10,11,15,16,17,18,22,23,24,25-hexadecafluoro-29*H*,31*H*-phthalocyanine; PFSEA: perfluorosebacic acid; THPP: meso-5, 10, 15, 20-tetra (4-hydroxylphenyl) porphyrin; AlPc: aluminium phthalocyanine; BODIPY-Br_2_: 4,4-difluoro-4-bora-3a,a-diaza-s-indacene brominated.

**Table 5:** Summary of publications about O_2_ microbubbles as O_2_ vehicle.

Ref	Application	O_2_ Source	PS	Energy of Excitation	Type of ROS	In Vitro	In Vivo
[67]	Hyperthermia/PDT	O_2_-loaded polymer bubbles	Ce6	660 nm, 100 mW∙cm^−2^, 10 min	^1^O^2^	RAW 264.7 cell line	Tramp-C1 tumour-bearing C57BL/6JNarl mice
[68]	PDT	O_2_ nanobubbles	Ce6	670 nm, 300 mW∙cm^−2^, 30 min	^1^O_2_	C6 glioma cell line	C6 glioma tumour-bearing mice

Ce6: Chlorin e6.

**Table 6:** Summary of publication about the decomposition of H_2_O_2_ by MnO_2._

Ref	Application	Catalyst	O_2_ Source	PS	Energy of Excitation	Type of ROS	IN VITRO	In Vivo
[75]	PDT	Mn (II) complex	H_2_O_2_	BODIPY derivatives	500–600 nm, 15 min	^1^O_2_, ^•^OH and others ROS	HepG-2 cell line	
[76]	PDT	MnO_2_	H_2_O_2_	Ce6	In vitro: 661 nm, 5 mW∙cm^−2^, 30 min In vivo: 661 nm, 5 mW∙cm^−2^, 1 h	^1^O_2_	4T1 cell line	4T1 tumour-bearing mice
[77]	PDT	MnO_2_	H_2_O_2_	DVDMS	In vitro: 630 nm, 75 mW∙cm^−2^, 5 min In vivo: 630 nm, 300 mW∙cm^−2^, 8 min	^1^O_2_	MCF-7 cell line	MCF-7 tumour-bearing mice
[78]	PDT	MnO_2_	H_2_O_2_	ICG	In vitro: 808 nm, 0.5 W∙cm^−2^, 10 min In vivo: 808 nm, 0.8 W∙cm^−2^, 10 min	^1^O_2_	SCC-7 cell line	SCC7 tumour-bearing mice
[79]	PDT	MnO_2_	H_2_O_2_	HMME	In vitro: 532 nm, 1.5 W∙cm^−2^, 2 min In vivo: 808 nm, 1.5 W∙cm^−2^, 1.5 min	^1^O_2_	MCF-7 cells line	S180 tumour model
[80]	PDT	manganese ferrite NPs	H_2_O_2_	Ce6	In vitro: 670 nm, 0.5 W∙cm^−2^, 30 s In vivo: 670 nm, 0.8 W∙cm^−2^, 5 min	^1^O_2_	U87 MG cell line	U87 tumour-bearing mice
[81]	PDT	MnO_2_	H_2_O_2_	HPPH	In vitro: 10 mW∙cm^−2^, 1 min In vivo: 630 nm, 10 mW∙cm^−2^, 10 min		U87MG cell line	U87MG tumour-bearing mice
[82]	PDT	MnO_2_	H_2_O_2_ and H^+^	MB	650 nm, 100 mW∙cm^−2^, 15 min	^1^O_2_	HeLa cells line	U14 tumour-bearing mice
[83]	PDT	MnO_2_ nanosheets	H_2_O_2_	Ce6	808 nm, 0.4 W∙cm^−2^, 60 min	^1^O_2_	B16F10 cell line	No
[84]	PDT	MnO_2_	H_2_O_2_	Ce6	In vitro: 980 nm, 0.5 W∙cm^−2^, 10 min In vivo: 660 nm, 0.5 W∙cm^−2^, 30 min	^1^O_2_	4T1 cell line	4T1 tumour-bearing mice
[85]	PDT	MnO_2_	H_2_O_2_	CD	In vitro: 635 nm, 100 mW∙cm^−2^, 30 min In vivo: 635 nm, 100 mW∙cm^−2^, 10 min	^1^O_2_	HeLa cell line	4T1-luc tumour-bearing mice
[86]	PDT	MnO_2_	H_2_O_2_	Ce6	660 nm, 100 mW∙cm^−2^, 10 min	^1^O_2_	HeLa cells line	No
[87]	PDT	MnO_2_	H_2_O_2_	PEGylated p–n heterojunction nanosheets	980 nm, 0.4 W∙cm^−2^, 5 min	^1^O_2_	HeLa cells and HEK 293 cell lines	
[88]	PDT	MnO_2_	H_2_O_2_	Gold nano-cage (AuNC)	808 nm, 0.8 W∙cm^−2^, 3 min	^1^O_2_	4T1 cell line	4T1 tumour-bearing mice
[89]	PDT	MnO NP	H_2_O_2_	IR808	808 nm, 0.5 W∙cm^−2^, 5 min	^1^O_2_	MCF-7 cancer cells	MCF-7 tumour-bearing mice
[90]	PDT	MnO_2_	H_2_O_2_	Ce6	In vitro: 660 nm, 5 mW∙cm^−2^, 30 min In vivo: 660 nm, 200 mW∙cm^−2^, 15 min	^1^O_2_	MB-49 cells line	Mice with orthotopic bladder cells
[91]	PDT	MnO_2_	H_2_O_2_	Ce6	In vitro: 638 nm, 5 mW∙cm^−2^, 30 min In vivo: 638 nm, 5 mW∙cm^−2^, 5 min	^1^O_2_	HeLa and HepG-2 cell lines	HeLa tumour-bearing mice
[92]	PDT	MnO_2_	H_2_O_2_ and H^+^	Poly(cyclopentadithiophene-alt-benzothiadiazole) (PCPDTBT)	In vitro: 808 nm, 0.44 W∙cm^−2^, 5 min In vivo: 808 nm, 0.30 W∙cm^−2^, 5 min	^1^O_2_	4T1 cells line	4T1 tumour-bearing mice
[93]	PDT	MnO_2_	H_2_O_2_	NMOFs composed by TCPP and Fe^3+^	660 nm, 50 mW∙cm^−2^, 15 min	^.^OH	4T1 cells line	4T1 tumour-bearing mice
[94]	PDT	MnO_2_	H_2_O_2_ and H^+^	FBP	In vitro: 660 nm, 150 mW∙cm^−2^, 3 min In vivo: 660 nm, 150 mW∙cm^−2^, 10 min	^1^O_2_	HeLa cell line	HeLa tumour-bearing mice
[95]	PTT/PDT	MnO_2_	H_2_O_2_	Cu_2-x_S	980 nm, 0.72 W∙cm^−2^, 5 min	^1^O_2_	MCF-7 and A549 cell lines	B16 tumour-bearing mice
[96]	PTT/PDT	MnO_2_	H_2_O_2_ and H^+^	Ce6	808 + 660 nm, 1.0 W∙cm^−2^, 10 min	^1^O_2_	HeLa cell line	HeLa tumour-bearing mice
[97]	PTT/PDT	MnO_2_	H_2_O_2_ and H^+^	IR780	785 nm PDT: 50 mW∙cm^−2^ PTT/PDT: 0.3 to 1.0 W∙cm^−2^	^1^O_2_	HepG2 and 3T3 cell lines	HepG2 tumour-bearing mice
[98]	Chemo-PDT	MnO_2_	H_2_O_2_	Ce6	In vitro: 660 nm, 5 mW∙cm^−2^, 30 min In vivo: 660 nm, 5 mW∙cm^−2^, 1 h	^1^O_2_	4T1 cell line	4T1 tumour-bearing mice
[99]	Chemo-PDT	MnO_2_	H_2_O_2_ and H^+^	g-C_3_N_4_	660 nm, 5 mW∙cm^−2^, 30 min	^1^O_2_	4T1 cell line	4T1 tumour-bearing mice
[100]	PDT	MnO_2_ nanosheets	H_2_O_2_, H^+^	Silicon phthalocyanine dihydroxide (SPCD)	In vitro: NIR, 1.5 W∙cm^−2^, 5 min In vivo: NIR, 2 W∙cm^−2^, 10 min	^1^O_2_	hc-4T1 cell line	4T 1 tumour-bearing mice
[101]	PDT and gene-silencing therapy	O_2_/Mn^2+^-evolving nanocomposite	H_2_O_2_	Ce6	In vitro: 635 nm, 30 mW∙cm^−2^, 5 min In vivo: 635 nm, 100 W∙cm^−2^, 5 min	^1^O_2_	MCF-7 cell line	MCF-7 tumour-bearing mice
[102]	PDT	Mn-Carbon dot	H_2_O_2_	Mn-Pc	635 nm, 50 mW∙cm^−2^, 10 min	^1^O_2_	HeLa cell line	4T1 tumour-bearing mice

BODIPY: 4,4-difluoro-4-bora-3a,a-diaza-s-indacene; Ce6: Chlorin e6; DVDMS: sinoporphyrin sodium; ICG: Indocyanine green; HMME: hematoporphyrin monomethyl ether; HPPH: 2-devinyl-2-(1-hexyloxiethyl)pyropheophorbide; MB: methylene blue; CD: carbon dot; NMOFs: nanoscale metal organic frameworks; TCPP: carboxyphenyl-porphyrin; FBP: fluorescein isothiocyanate (FITC)-labelled peptide-functionalized black phosphorus; g-C_3_N_4_: Graphitic carbon nitrides; Mn-Pc: Mn(II) phthalocyanines.

**Table 7:** Summary of publications about the decomposition of H_2_O_2_ by catalase.

Ref	Application	Catalyst	O_2_ Source	PS	Energy of Excitation	Type of ROS	In Vitro	In Vivo
[103]	PDT	Catalase	H_2_O_2_	MB	635 nm, 100 mW∙cm^−2^, 5 min	^1^O_2_	HaCaT, U87-MG, MCF-7 and SKOV-3 cell lines	U87-MG tumour bearing mice
[104]	PDT	Catalase	H_2_O_2_	MB	808 nm, 1 W∙cm^−2^, 5 min	^1^O_2_	PC-3 and bMSCs cell lines	No
[105]	PDT	Catalase	H_2_O_2_	MB	In vivo: 635 nm, 50 mW∙cm^−2^, 10 min	^1^O_2_	SW1990 and 293 T cell lines	SW1990 tumour-bearing mice
[106]	PDT	Catalase	H_2_O_2_	AlPcS_4_	In vitro: 660 nm, 30 mW∙cm^−2^, 1 min In vivo: 660 nm, 220 mW∙cm^−2^, 5 min	^1^O_2_	HeLa, COS7, SCC-7 and 4T1 cell lines	HeLa tumour-bearing mice
[72]	PDT	Catalase	H_2_O_2_	Ce6	In vitro: 660 nm, 5 mW∙cm^−2^, 30 min In vivo: 660 nm, 5 mW∙cm^−2^, 60 min	^1^O_2_	4T1 cell line	4T1 tumour-bearing mice
[107]	PDT	Catalase	H_2_O_2_	Ce6	660 nm, 5 mW∙cm^−2^, 60 min	^1^O_2_	4T1 cell line	4T1 tumour-bearing mice
[108]	PDT	Catalase	H_2_O_2_	Ce6	In vitro: 650 nm, 100 mW∙cm^−2^, 10 min, 25 J∙cm^−2^ In vivo: 650 nm, 100 mW∙cm^−2^, 10 min	^1^O_2_	CAL-27, HeLa, L929 cell lines	CAL-27 tumour-bearing mice
[109]	PDT	Catalase	H_2_O_2_	HMME	LED, 3000 mW∙cm^−2^ for 2 min	^1^O_2_	B16-F10 cell line	Female C57 mice
[111]	PDT	Thiolate catalase (CAT)	H_2_O_2_	RB	Green LED array, 40 mW∙cm^−2^, 10 min	^1^O_2_	RAW 264.7 cell line	No
[112]	PDT	Biomimetic nanothylakoid	H_2_O_2_	chlorophyll	660 nm 1 W∙cm^−2^, 10 min	^1^O_2_	4T1 cell line	4T1 tumour-bearing mice
[114]	PDT	Catalase	H_2_O_2_	THPP	In vitro: 660 nm, 5 mW∙cm^−2^, 30 min In vivo: 660 nm, 5 mW∙cm^−2^, 60 min	^1^O_2_	4T1 cell line	4T1 tumour-bearing mice
[115]	PDT/lung cancer	AuNCs-NH_2_	H_2_O_2_	PpIX	532 nm, 200 mW∙cm^−2^, 5 J per well	^1^O_2_	H 460 cell line	No
[116]	PDT	G-quadruplex-hemin DNAzyme	H_2_O_2_	Ce6	In vitro: 660 nm, 5 mW∙cm^−2^, 30 min In vivo: 660 nm, 5 mW∙cm^−2^, 60 min		4T1 cell line	4T1 tumour-bearing mice
[117]	PDT	MnFe-LDH	H_2_O_2_	MB	In vitro: 650 nm, 100 mW∙cm^−2^, 10 min In vivo: 650 nm, 100 mW∙cm^−2^, 15 min	^1^O_2_	HeLa cell line	U14 tumour-bearing mice
[118]	PDT	PB NPs	H_2_O_2_	ZnPc	671 nm, 400 mW∙cm^−2^, 5 min	^1^O_2_	HeLa, A549 and 4T1 cell lines	4T1 tumour-bearing mice
[119]	PDT	PB NPs	H_2_O_2_	Ce6	In vitro: 660 nm, 1.0 W∙cm^−2^, 5 min In vivo: 660 nm, 1.0 W∙cm^−2^, 2 min	^1^O_2_	U87MG and HUVEC cell lines	U87MG tumour-bearing mice
[120]	PDT	Fe(III)	H_2_O_2_	MB	650 nm, 100 mW∙cm^−2^, 15 min	^1^O_2_	HeLa cell line	U14 tumour-bearing mice
[121]	PDT	Fe(III)	H_2_O_2_	MB	In vitro: 650 nm, 100 mW∙cm^−2^, 10 min In vivo: 650 nm, 100 mW∙cm^−2^, 15 min	^1^O_2_	HeLa cell line	-
[122]	PDT	Fe(III)	H_2_O_2_	Ce6, TPEDC, TPETCF	In vitro: white light excitation, 50 mW∙cm^−2^ 5 min In vivo: white light excitation, 50 mW∙cm^−2^, 10 min	^1^O_2_	4T1 cell line	4T1 tumour-bearing mice
[123]	Immuno-PDT	Fe (III)	H_2_O_2_	TBP	In vitro: 650 nm, 20 mW∙cm^−2^, 15 min In vivo: 650 nm, 100 mW∙cm^−2^, 7.5 min	^1^O_2_	CT26 cell line	CT26 tumour-bearing mice
[124]	PDT	Cy5- aptamer-heme (H1) and 3’-heme labelled oligonucleotide (H2)	H_2_O_2_	BPNS	In vitro: 660 nm, 150 mW∙cm^−2^, 3 min In vivo: 660 nm, 150 mW∙cm^−2^, 10 min	^1^O_2_	HeLa cell line	4T1 tumour-bearing mice

MB: Methylene Blue; AlPcS_4_: Al(III) phthalocyanine chloride tetrasulfonic acid; Ce6: Chlorin e6; HMME: Hematoporphyrin monomethyl ether; RB: Rose Bengal; THPP: meso-5, 10, 15, 20-tetra(4-hydroxyphenyl) porphyrin; AuNCs-NH_2_: peroxidase amine-terminated PAPAM dendrimer-encapsulated gold NP; PPIX: Protoporphyrin IX; MnFe-LDH: Manganese-iron layered double oxide; PB NPs: Prussian Blue nanoparticles; ZnPc: Zinc phthalocyanine; TPEDC: 2-((4’-(2,2-bis(4-methoxyphenyl)-1-phenylvinyl)-1,1’-biphenyl-4-yl)(phenyl)methylene)malonitrile; TPETCF: (*E*)-2-(4-(4-(2,2-bis(4-methoxyphenyl)-1-phenylvinyl) styryl)-3-cyano-5,5-dimethylfuran-2(5*H*)-ylidene)malonitrile; TBP: 5,10,15,20-tetra(*p*-benzoato)porphyrin. BPNS: black phosphorus nanosheet.

**Table 8:** Summary of publications about H_2_O_2_ decomposition by Pt NPs.

Ref	Application	Catalyst	O_2_ Source	PS	Energy of Excitation	Type of ROS	In Vitro	In Vivo
[125]	PDT	Pt NP	H_2_O_2_	TCPP	In vitro: 638 nm, 1.0 W∙cm^−2^, 10 min In vivo: 638 nm, 1.0 W∙cm^−2^, 8 min	^1^O_2_	HeLa and 4T1 cell lines	H22 tumour-bearing mice
[126]	PDT	Pt NP	H_2_O_2_	Ce6	660 nm, 150 mW∙cm^−2^, 5 min + 808 nm, 500 mW∙cm^−2^, 5 min	^1^O_2_	4T1 cell line	4T1 tumour-bearing mice
[127]	PDT	Pt NP	H_2_O_2_	zirconium-TCPP	In vitro: 660 nm, 30 mW∙cm^−2^, 3 min In vivo: 660 nm, 200 mW∙cm^−2^, 5 min	^1^O_2_	CT26 cell line	CT26 tumour-bearing mice
[128]	PDT	Pt NP	H_2_O_2_	Ce6	660 nm, 50 mW∙cm^−2^; 5 min	^1^O_2_	A549 cell line	No
[129]	PDT	Pt NP	H_2_O_2_	BPNS	660 nm, 1.0 W∙cm^−2^, 10 min	^1^O_2_	4T1 cell line	4T1 tumour-bearing mice

TCPP: carboxyphenylporphyrin; BPNS: black phosphorus nanosheet.

**Table 9:** Summary of publications about H_2_O_2_ decomposition by others ways.

Ref	Application	Catalyst	O_2_ Source	PS	Energy of Excitation	Type of ROS	In Vitro	In Vivo
[130]	PDT	Light-triggered polymeric vesicle coupled to Ce6 and encapsulating H_2_O_2_.	H_2_O_2_	Ce6	805 nm, 1000 mW∙cm^−2^, 3 min - 660 nm, 100 mW∙cm^−2^, 10 min	^1^O_2_	BxPC-3 cell line	BxPC-3 tumour-bearing mice
[131]	PTT/PDT	Hydrophilic H_2_O_2_/poly(vinylpyrrolidone) complex	H_2_O_2_	IR780	808 nm, 0.5 W∙cm^−2^, 3 min	ROS	HepG2 cell line	HepG2 tumour-bearing mice
[132]	PDT	Cream	H_2_O_2_	5-ALA methyl ester (MAL, Metvix^®^)	570–670 nm, 105 mW∙cm^−2^, 16 min, *in vvo*	nd	Erithema cell line	40 healthy volunteers
[133]	PDT	BiOI/BiOIO_3_ nanocomposite	H_2_O_2_	BiOI/BiOIO_3_	650 nm, 500 mW∙cm^−2^, 15 min	^1^O_2_ and ^●^OH	HeLa cell line	4T1 tumour-bearing mice
[134]	Chemo-PDT	Fe(OH)-modified UCNP	H_2_O_2_	Ce6	In vitro: 808 nm, 1 W∙cm^−2^, 10 min In vivo: 808 nm, 1 W∙cm^−2^, 30 min	^1^O_2_, ^●^OOH and ^●^OH	4T1 cell line	4T1 tumour-bearing mice

nd: not determined; Ce6: chlorin e6; 5-ALA: 5-sminolevulinic acid; UCNP: Up-conversion nanoparticles.

**Table 10:** Summary of publication about water splitting.

Ref	Application	Catalyst	O_2_ Source	PS	Energy of Excitation	Type of ROS	In Vitro	In Vivo
[136]	PDT	CaO_2_	Water splitting	RB	980 nm, 2 W∙cm^−2^, 5 s	^1^O_2_	HT29 cell line	HT29 tumour-bearing mice
[137]	PDT	CaO_2_	Water splitting	MB	In vitro: 660 nm, 30 mW∙cm^−2^, 30 + 60 s In vivo: 658 nm, 280 mW∙cm^−2^, 2 + 8 min	^1^O_2_	4T1 cell line	4T1 tumour-bearing mice
[138]	PDT	CaO_2_	Water splitting	RB	White light, 205 J∙cm^−2^, 5 min	^1^O_2_	BxPC-3 cell line	MIAPaCa-2 tumour-bearing mice
[139]	PDT	C_3_N_4_ NPs	Water splitting	PpIX	630 nm, 80 mW∙cm^−2^, 10 min	nd	4T1 cell line	4T1 tumour-bearing mice
[140]	PDT	Fe-C_3_N_4_ NPs	Water splitting	Ru(bpy)_3_^2+^	In vitro: 800 nm - 2 photons, 2.7 W, 3 or 5 min In vivo: 800 nm - 2 photons, 2.7 W, 5 min	^1^O_2_	4T1 cell line	4T1 tumour bearing Balb/ c mice
[141]	PDT	C_3_N_4_ nanosheets	Water splitting	CD	In vitro: 980 nm, 500 mW∙cm^−2^, 5 min In vivo: 980 nm, 500 mW∙cm^−2^, 10 min	^●^OH O_2_^−●^	HeLa cell line	U14 tumour-bearing mice
[142]	PDT	Thylakoid membrane from plants	Water splitting	Ag, SiO_2_ or ZnO NPs	660 nm, 155 mW∙cm^−2^, 2 min	ROS	CT26 cell line	CT26 tumour-bearing mice
[144]	PTT/PDT	Bi_2_WO_6_ NPs	Water splitting	Bi_2_WO_6_ NPs	808 nm, 1 W∙cm^−2^, 5 to 20 min	^●^OH	HeLa cell line	HeLa tumour-bearing mice
[145]	PDT	Au-semiconductor nanocomposite	Water splitting		In vitro: 450 nm, 500 mW∙cm^−2^, 10 min In vivo: 450 nm, 500 mW∙cm^−2^, 30 min	nd	4T1 cell line	4T1 tumour-bearing mice

nd: not determined; RB: Rose Bengal; MB: Methylene Blue; C_3_N_4_: Carbon nitride; PPIX: Protoporphyrin IX; Ru(bpy)_3_^2+^: tris(bipyridyl)cationic Ru complexes; CD: Carbon dot.

**Table 11:** Summary of publications about the destruction of ECM to modify TME.

Ref	Application	Target	Additional Compound	PS	Energy of Excitation	Type of ROS	In Vitro	In Vivo
[146]	PDT	Hyaluronan of ECM	Hyaluronidase	Ce6	660 nm, 2 mW∙cm^−2^ 60 min	^1^O_2_ and others ROS	No	4T1 tumour-bearing mice
[147]	PDT	Collagen of ECM	Collagenase	Ce6	660 nm, 5 mW∙cm^−2^, 60 min	nd	No	4T1 tumour-bearing mice

nd: not determined; Ce6: Chlorin e6.

**Table 12:** Summary of publications about the reduction of tumour O_2_ consumption to modify TME.

Ref	Application	Additional Compound	PS	Energy of Excitation	Type of ROS	In Vitro	In Vivo
[148]	PDT	Met	HCe6	In vitro: 660 nm, 10 min In vivo: 660 nm, 30 min, 0.035 W∙cm^−2^	nd	4T1 cell line	4T1 tumour-bearing mice
[149]	PDT	Met	W_18_O_49_	808 nm, 1 W∙cm^−2^, 10 min	^1^O_2_	Raji cell line	Raji lymphoma-bearing mice
[150]	PDT	TAM	Ce6	660 nm, 5 mW∙cm^−2^, 30 min	^1^O_2_	4T1 cell line	4T1 tumour-bearing mice
[151]	PDT	Ato	ICG-BSA nanocomplex	808 nm, 1 W∙cm^−2^, 5 min	ROS	HeLa cell line	HeLa tumour-bearing mice

nd: not determined; Met: Metformin; HCe6: Hydrophobic Chlorin e6; TAM: Tamoxifen; Ato: Atovaquone; ICG-BSA: Indocyanine Green-Bovine Serum Albumin.

**Table 13:** Summary of publications about the use of TPZ for Chemo-PDT.

Ref	Application	Supplementary Therapy Used	Chemo-Drug	PS	Energy of Excitation	Type of ROS	In Vitro	In Vivo
[156]	Chemo-PDT	Chemotherapy	TPZ	SPCD	In vitro: 980 nm, 0.7 W∙cm^−2^, 5 min In vivo: 980 nm, 1.4 W∙cm^−2^, 15 min	^1^O_2_ and others ROS	HeLa cell line	HeLa tumour-bearing mice
[157]	Chemo-PDT	Chemotherapy	TPZ	TPC	650 nm, 1.2 W∙cm^−2^,10 min	^1^O_2_	MCF-7 cell line	MCF-7 tumour-bearing mice
[158]	Chemo-PDT	Chemotherapy	TPZ	PCN-224	In vitro: 660 nm, 30 mW∙cm ^−2^, 5 min In vivo: 660 nm, 220 mW∙cm ^−2^, 10 min	^1^O_2_	4T1 and COS7 cell line	4T1 tumour-bearing mice
[159]	Chemo-PDT	Chemotherapy	TPZ	ICG	In vitro: 808 nm, 2 W∙cm^−2^, 3 min In vivo: 808 nm, 2 W∙cm^−2^, 5 min	^1^O_2_	4T1 cell line	4T1 tumour-bearing mice
[160]	Chemo-PDT	Chemotherapy	TPZ	ICG	405 nm, 0.75 W∙cm^−2^, 5 min and 808 nm, 1.0 W∙cm^−2^, 10 min	^1^O_2_	4T1 cell line	4T1 tumour-bearing mice
[161]	Chemo-PDT	Chemotherapy	TPZ	TCPP	In vitro: 635 nm, 12 mW∙cm^−2^, 9 min In vivo: 635 nm, 0.1 W∙cm^−2^, 30 min	^1^O_2_	4T1 cell line	4T1 tumour-bearing mice
[162]	Chemo-PDT	Chemotherapy	TPZ	IR780	808 nm, 1 W∙cm^−2^, 3 min	^1^O_2_	4T1 cell line	4T1 tumour-bearing mice
[163]	Chemo-PDT	Chemotherapy	TPZ	Ce6	670 nm, 0.48 W∙cm^−2^, 10 min	^1^O_2_	MCF-7 cell line	MCF-7 tumour-bearing mice
[164]	Chemo-PDT	Chemotherapy	TPZ	Diketopyrrolopyrrole (DPP)-based compound	660 nm, 1.5 W∙cm^2^, 10 min	^1^O_2_	MCF-7 cell line	No

TPZ: Tirapazamine; SPCD: silicon phthalocyanine dihydroxide; TPC: 5-(4-carboxyphenyl)-10, 15, 20-tris(3-hydroxyphenyl) chlorin; PCN-224: porphyrinic metal organic framework; ICG: Indocyanine green; TCPP: Carboxyphenyl-porphyrin; Ce6: Chlorin e6.

**Table 14:** Summary of publications about the use of DOX for chemo-PDT.

Ref	Application	Supplementary Therapy Used	Drug	PS	Energy of Excitation	Type of ROS	In Vitro	In Vivo
[165]	Chemo-PDT	Chemotherapy	DOX	2-nitroimidazole (NI)	In vitro: light, 0.1 W∙cm^−2^, 5 or 20 min In vivo: 532 nm, 0.1 W∙cm^−2^, 5 min or 635 nm, 0.1 W∙cm^−2^, 5 min	^1^O_2_	HeLa cell line	HeLa tumour-bearing mice
[166]	Chemo-PDT	Chemotherapy	DOX	Ce6	In vitro: 660 nm, 100 mW∙cm^−2^, 5 min In vivo: 660 nm, 100 mW∙cm^−2^, 10 min	^1^O_2_	MCF-7 cell line	MCF-7 tumour-bearing mice
[167]	Chemo-PDT	Chemotherapy	DOX	Ce6	In vitro: 660 nm, 100 mW∙cm^−2^, 2 min In vivo: 660 nm, 100 mW∙cm^−2^, 20 min	^1^O_2_	MCF-7 cell line	Female BALB/c nude mice
[168]	Chemo-PDT	Chemotherapy	DOX	Ce6	980 nm, 0.5 W∙cm^−2^, 5 min	ROS	HeLa cell line	U14 tumour-bearing mice
[169]	Chemo-PDT	Chemotherapy	DOX	RB	In vitro: 808 nm, 0.5 W∙cm^−2^, 10 min In vivo: 808 nm, 0.5 W∙cm^−2^, 5 min	^1^O_2_	L929 cell line	H22 tumour bearing mice
[170]	Chemo-PDT	Chemotherapy	DOX	Ce6	In vitro: 660 nm, 2 mW∙cm^−2^, 30 min In vivo: 660 nm, 10 mW∙cm^−2^, 30 min	^1^O_2_	LLC cell line	LLC tumour bearing mice
[171]	Chemo-PDT	Chemotherapy	DOX	PPy	638 nm, 1 W∙cm^−2^, 10 min	^1^O_2_	HepG2 cell line	No
[172]	Chemo-PDT	Chemotherapy	DOX	Ce6	660 nm, 100 mW∙cm^−2^, 10 min	^1^O_2_	4T1 cell line	4T1 tumour-bearing mice

DOX: Doxorubicin; Ce6: Chlorin e6; RB: Rose Bengal; PPy: Polypyrrole.

**Table 15:** Summary of publications about the use of AQ4N for chemo-PDT.

Ref	Application	Supplementary Therapy Used	Drug	PS	Energy of Excitation	Type of ROS	In Vitro	In Vivo
[173]	Chemo-PDT	Chemotherapy	AQ4N	Ce6	In vitro: 660 nm, 2 mW∙cm^−2^, 30 min In vivo: 660 nm, 2 mW∙cm^−2^, 1 h	^1^O_2_	4T1 cell line	4T1 tumour-bearing mice
[174]	Chemo-PDT	Chemotherapy	AQ4N	Verteporfin	In vitro: 690 nm, 30 mW∙cm^−2^, 10 min In vivo: 690 nm, 50 mW∙cm^−2^, 20 min	^1^O_2_	PC-3 cell line	PC-3 tumour-bearing mice
[175]	Chemo-PDT	Chemotherapy	AQ4N	Photochlor (HPPH)	In vitro: 671 nm, 100 mW∙cm^−2^, 6 min In vivo: 671 nm, 100 mW∙cm^−2^, 10 min	ROS	U87MG cell line	U87MG tumour-bearing mice

Ce6: Chlorin e6.

**Table 16:** Summary of publications about the use of Pt(IV) drugs for chemo-PDT.

Ref	Application	Supplementary Therapy Used	Drug	PS	Energy of Excitation	Type of ROS	In Vitro	In Vivo
[176]	Chemo-PDT	Chemotherapy	Pt (IV) complex	No	In vitro: 396 nm, 5 mW·cm^−2^, 5min In vivo: 396 nm, 0.4 W·cm^−2^, 10 min	^●^OH and ^1^O_2_	A549 cell line	A549 tumour- bearing mice
[177]	Chemo-PDT	Chemotherapy	Pt (IV) Up conversion NP	Ce6	980 nm near-infrared light convert into 365 nm and 660 nm emissions In vitro: 0.85 W∙cm^−2^, 5 min In vivo: 0.80 W∙cm^−2^, 10 min	^1^O_2_	L929 cell line	HeLa, B16, HCT116 or MDA-MB-231 tumour-bearing mice
[178]	Chemo-PDT	Chemotherapy	cis-Pt (IV)	THPP	In vitro: 660 nm, 5 mW·cm^−2^, 20 min In vivo: 660 nm, 5 mW·cm^−2^, 45 min	^1^O_2_	4T1 cell line	4T1 tumour-bearing mice

Ce6: Chlorin e6; THPP: mesotetra(p-hydroxyphenyl)porphine.

**Table 17:** Summary of publications about the use of HIF-1α inhibitors for antiangiogenic-PDT.

Ref	Application	Therapy Used	Drug	PS	Energy of Excitation	Type of ROS	In Vitro	In Vivo
[179]	Antiangiogenic-PDT	Antiangiogenic	HIF-1α siRNA	Photosan	640 nm, 320 mW∙cm^−2^, 100 J∙cm^−2^	nd	SCC4 and SAS cell lines	SCC4 and SAS tumour bearing nude mice.
[180]	Antiangiogenic-PDT	Antiangiogenic	HIF-1α siRNA	Cationic porphyrin-grafed lipid	650 nm, 200 mW, 10 min	^1^O_2_	MDA-MB-231 cell line	MDA-MB-231 tumour-bearing mice
[181]	Antiangiogenic-PDT	Antiangiogenic	Acriflavine	ZnPc	671 nm, 500 mW, 15 J∙cm^−2^	nd	A431 cell line	No
[182]	Antiangiogenic-PDT	Antiangiogenic	Acriflavine	ZnPc	671 nm, 500 mW, 15 J∙cm^−2^	ROS	SK-ChA-1 cell line	No

nd: not determined; HIF-1α siRNA: hypoxia-inducible factor-1α small interfering RNA; ZnPc: zinc phthalocyanine.

**Table 18:** Summary of publications about the use of VEGF inhibitor for antiangiogenic-PDT.

Ref	Application	Therapy Used	Drug	PS	Energy of Excitation	Type of ROS	In Vitro	In Vivo
[183]	Antiangiogenic-PDT	Antiangiogenic	EMAP-II or IM862	Photofrin	In vitro: 570–650 nm, 0.35 mW∙cm^−2^ In vivo: 630 nm, 75 mW∙cm^−2^, 200 J∙cm^−2^	nd	BA cell line	BA tumour-bearing mice
[184]	Antiangiogenic-PDT	Antiangiogenic	SU5416 and SU6668	hypericin	Halogen light with red acetate filter, 47.7 J∙cm^−2^, 60 mW∙cm^−2^	nd	No	CNE2 tumour-bearing mice
[185]	Antiangiogenic-PDT	Antiangiogenic	sunitinib, sorafenib and axitinib/bevacizumab	Visudyne	420 nm, 5 J∙cm^−2^, 35 mW∙cm^−2^	nd	No	A2780 tumour-bearing mice
[186]	Antiangiogenic-PDT	Antiangiogenic	VEGF-A siRNA	Photosan	640 nm In vitro: 10 J∙cm^−2^, 159 s In vivo: 320 mW∙cm^−2^, 100 J∙cm^−2^, 11 min	nd	SCC4 and SAS cell lines	SCC4 and SAS tumour bearing nude mice .
[187]	Antiangiogenic-PDT	Antiangiogenic	5,6-dimethylxanthenone-4-acetic acid	DPP-4	660 nm, 0.8 W∙cm^−2^, 4 min	^1^O_2_ and others ROS	HeLa and HUVEC cell lines	HeLa tumour-bearing mice

nd: not determined; DPP-4: diketopyrrolopyrrole.

**Table 19:** Summary of publications about the use of others inhibitors for antiangiogenic-PDT.

Ref	Application	Therapy Used	Drug	PS	Energy of Excitation	Type of ROS	In Vitro	In Vivo
[188]	Antiangiogenic-PDT	Antiangiogenic	celecoxib or NS-398	Photofrin	570–650 nm, 0.35 mW∙cm^−2^, 0 to 525 J∙cm^−2^, 0 to 150 s	nd	BA cell line	BA tumour-bearing mice
[189]	Antiangiogenic-PDT	Antiangiogenic	Chalcone	phthalocyanine	red light (>600 nm), 3.6 J∙cm^−2^	^1^O_2_	HT29 cell line	No
[190]	Antiangiogenic-PDT	Antiangiogenic	DHA	5-ALA	630 nm, 25 W∙cm^−2^	nd	Eca109 cell line	Eca109 tumour-bearing mice
[191]	Antiangiogenic-PDT	Antiangiogenic	DHA	5-ALA	630 nm, 20 or 25 W∙cm^−2^	nd	Eca109 and Ec9706 cell lines	No
[192]	Antiangiogenic-PDT	Antiangiogenic	acetazolamide	AZBPS	660 nm, 2 W∙cm^−2^, 30 min	^1^O_2_	MDA-MB-231 and MCF7 cell line	MDA-MB-231 tumour-bearing nude mice

nd: not determined; AZBPS: acetazolamide conjugated BODIPY; 5-ALA: 5-aminolevulinic acid.

**Table 20:** Summary of publications about PTT/PDT.

Ref	Application	Supplementary Therapy Used	PS	Energy of Excitation	Type of ROS	In Vitro	In Vivo
[193]	PTT/PDT	PTT	Photofrin and Porphysome	Photofrin: 635 nm, 200 mW, 318 s Porphysome: 671 nm, 200 mW, 18 s; PTT: 671 nm, 750 mW, 85 s	nd	No	KB tumour-bearing mice
[194]	PTT/PDT	PTT	Iron porphyrin	600 nm, PDT: 50 mW∙cm^−2^, 10 min PTT: 1.0 W∙cm^−2^ for 5 min,	ROS	4T1 cell line	4T1 tumour-bearing mice
[195]	PDT	External heating	Ce6	In vitro: 660 nm, 50 mW∙cm^−2^, 5 min In vivo: 660 nm, 200 mW∙cm^−2^, 20 min	^1^O_2_	4T1 cell line	4T1 tumour-bearing mice
[196]	PTT/PDT	PTT	*h*Ce6	NIR irradiation: 785 nm, 1 W∙cm^−2^, 10 min Light irradiation: 660 nm, 2 mW∙cm^−2^, 30 min	nd	4T1 cell line	4T1 tumour-bearing mice

nd: not determined; Ce6: Chlorin e6; *h*Ce6: *h*exylamine conjugated chlorin e6.

**Table 21:** Summary of publications about the use of PDT type I strategy for a PDT Hypoxia-independent.

Ref	Application	Hypoxia-Independent Strategy	PS	Energy of Excitation	Type of ROS	In Vitro	In Vivo
[199]	PDT	Modulation of the mechanism of photoactivation by micelles	mTHPP	532 nm, 20 mW∙cm^−2^, 10 min	^1^O_2_ and others ROS	H2009, A549 and PC-3 cell lines	No
[16]	PDT	Alginate formulation to switch photochemistry of PS from type II to type I	MB	600 nm, 6 mW∙cm^−2^	O_2_^−●^ and others ROS	MDAMB231, 4T1, SKBR3, and MCF7 cell lines	
[200]	PDT	Superoxide radical generator	ENBS-B	660 nm, 14.4 J∙cm^−2^, 15 min	O_2_^−●^, ^●^OH	HepG2 cell line	HepG2 tumour-bearing mice
[201]	PTT/PDT	Fenton reaction	CFNs	650 nm, 0.49 W∙cm^−2^, 15 min and 808 nm 1.3 W∙cm^−2^, 10 min	^●^OH, O^2−●^	HeLa cell line	U14 tumour-bearing mice
[202]	PDT	Type I PSs	Ru (II) complexes	In vitro: white light (400–800 nm) 30 mWcm^−2^, 10 min In vivo: xenon lamp, 250 mW∙cm^−2^, 15 min	^1^O_2_ and others ROS	HeLa cell line	HeLa tumour-bearing mice
[203]	PDT	Fenton reaction	Fe_3_O_4_@MIL-100(Fe)-UCNP	In vitro: 980 nm laser 0.9 Wcm^−2^, 10 min In vivo: 980 nm laser 0.9 Wcm^−2^, 15 min	^●^OH	HeLa cell line	U14 tumour-bearing mice
[204]	PDT	Carbon radical generator	Ru(III) complexes	470 nl LED	Carbon radicals	SKOV-3 cell line	No
[205]	PDT	Type I reaction	Os(biq)_2_(phen)](PF_6_)_2_, Os(biq)_2_(IP)](PF_6_)_2_ and Os(biq)_2_(dppn)](PF_6_)_2_	625 nm (90 J∙cm^−2^ = 450 mW∙cm^−2^, 200 s) or 808 nm light (600 J∙cm^−2^ = 900 mW∙cm^−2^, 667 s)	^1^O_2_ and oxygen-independent pathways	HT1376 and U87 cell lines	CT26.CL25 tumour-bearing mice

*m*THPP: 5,10,15,20-Tetrakis(*meso*-hudroxyphenyl)porphyrin; MB: Methylene Blue; CFNs: copper ferrite nanosphere.

**Table 22:** Summary of publications about the use of O_2_ independent cytotoxic compounds for PDT hypoxia-independent.

Ref	Application	Hypoxia-Independent Strategy	PS	Energy of Excitation	Type of ROS	In Vitro	In Vivo
[206]	PDT	Photocontrollable cytotoxic peptidomimetic (photoisomerization)	Diaryletene-derived peptodomimetics	Visible light, 100 mW∙cm^−2^, 20 min	No	HeLa and COLO-205 cell lines	LLC tumour-bearing mice
[207,208]	PDT	Photoacid generators to induce an imbalance of the pH of tumour cells	PAG	In vitro: 377 nm, 5.4 mW∙cm^−2^ In vivo: two-photon (710 nm), 2.0 mW∙cm^−2^	No	HCT-116 cell line	No

PAG: photacid generator.

**Table 23:** Summary of publications about the use of NO donor for PDT hypoxia-independent.

Ref	Application	Hypoxia-Independent Strategy	PS	Energy of Excitation	Type of ROS	Others Radicals	In Vitro	In Vivo
[209]	PDT	SNP: NO donor to reduce O_2_ consumption	Tetraphenylporphyrin	660 nm, 20 mW. cm^−2^, 3 or 10 min	^1^O_2_	NO + others ROS	4T1 cells	4T1 tumour-bearing mice
[210]	PDT	Compound able to produce both NO and ^1^O_2_ under irradiation	Ru complex	660 nm, 5 J∙cm^−2^	^1^O_2_	NO	B16F10 cell line	No
[211]	PDT	NO-generation induced by ROS production (NO donor: L-arginine)	Porphyrinic metal-organic framework	In vitro: 660 nm, 30 mW∙cm^−2^, 8 min In vivo: 660 nm, 200 mW∙cm^−2^, 10 min	^1^O_2_	NO	4T1 cell line	4T1 tumour-bearing mice
[212]	PDT	Glutathione-sensitive supramolecular NO nanogenerator	Ce6	In vitro: 660 nm laser, 0.2 W, 2 min In vivo: 660 nm laser, 0.5 W, 5 min		NO + Peroxynitrite anions	MCF-7 cell line	MCF-7 tumour-bearing mice

NO: Nitric oxide; Ce6: Chlorin e6.

**Table 24:** Summary of publications about ^1^O_2_ donor for a PDT Hypoxia-independent.

Ref	Application	Hypoxia-Independent Strategy	PS	Energy of Excitation	Type of ROS	In Vitro	In Vivo
[213]	PTT/PDT	Use of ^1^O_2_ donor: endoperoxide of DPA	tetraphenylporphyrin	In vitro: 808 nm, 0.5 W∙cm^−2^, 1 min In vivo: 808 nm, 1.0 W∙cm^−2^, 0–5 min	^1^O_2_ and others ROS	HepG2 cell line	HepG2 tumour-bearing mice
[214]	PTT/PDT	Use of ^1^O_2_ donor: DPAE	DPAE	808 nm, 1.5 W∙cm^−2^, 5 min	ROS	4T1 cell line	4T1 tumour-bearing mice

DPA: diphenylanthracene; DPAE: diphenylanthracene endoperoxide.

**Table 25:** Summary of publications about the use of active compounds in both normoxic and hypoxic conditions for PDT hypoxia-independent.

Ref	Application	PS	Energy of Excitation	Type of ROS	In Vitro	In Vivo
[215]	PDT	Pt (II) porphyrins linked to cationic oligofluorenes arms	In vitro: 520 nm, 10.0 mW∙cm^−2^, 10 min In vivo: 520 nm, 160 mW∙cm^−2^, 10 min	^1^O_2_	HeLa cell line	HeLa tumour-bearing mice
[216]	PDT	OR141	White light, 15 min	^1^O_2_	Endothelial cells	Human colon carcinoma xenograft model
[217]	PDT	Mito-DAD	470 nm, 16 mW∙cm^−2^, 3 min in normoxia and 5 min in hypoxia	^1^O_2_	HeLa cell line	No
[218]	PDT	Porphyrazines derivatives	690 nm, 2 J∙cm^−2^	^1^O_2_	LNCaP cell line	No

DAD: donor-acceptor-donor.

**Table 26:** Summary of publications about the use of Hypoxia-reducible compounds for PDT hypoxia-dependent.

Ref	Application	Hypoxia-Dependent Strategy	PS	Energy of Excitation	Type of ROS	In Vitro	In Vivo
[219]	Chemo-PDT	Hypoxia-cleaved Azobenzene	Ce6	In vitro: 670 nm, 50 mW∙cm^−2^, 6 min In vivo: 670 nm, 50 mW∙cm^−2^, 10 min	^1^O_2_	HeLa and NIH3T3 cell lines	HeLa tumour-bearing mice
[220]	Chemo-PDT	Hypoxia-cleaved Azobenzene	TMPyP4	660 nm, 1 W∙cm^−2^, 30 min	ROS	HepG2 and L02 cell lines	No
[222]	PDT	Hypoxia-cleaved Azobenzene	Ce6	In vitro: 660 nm, 100 mW∙cm^−2^, 10 and 5 min In vivo: 660 nm, 200 mW∙cm^−2^, 30 min	^1^O_2_	LLC cell line	LLC tumour-bearing mice
[221]	PDT	Hypoxia-cleaved Azobenzene	azoSeR	535 nm, 28 mW∙cm^−2^, 3 min	^1^O_2_	A549 cell line	No
[223]	Chemo-PDT	Hypoxia-cleaved Azobenzene	Ce6	In vitro: 660 nm, 8 mW∙cm^−2^, 6 min In vivo: 660nm, 8 mW∙cm^−2^, 30 min	^1^O2	MCF-7 cell line	MCF-7 tumour-bearing mice
[224]	Chemo-PDT	Hypoxia-cleaved Azobenzene	Ce6	In vitro: 671 nm, 10 mW∙cm^−2^, 5 min In vivo: 671 nm, 150 mW∙cm^−2^, 10 min	^1^O2	HeLa cell line	HeLa tumour-bearing mice
[225]	PDT	Hypoxia-reducible compound by NTR	DiBDP substituted with a nitro group	520 nm, 100 mW∙cm^−2^, 5 min	^1^O_2_	HeLa cell line	HeLa tumour-bearing mice

Ce6: Chlorin e6; TMPyP4: 5,10,15,20-tetrakis-(1-methyl-4-pyridyl)-21*H*,23*H*-porphine; azoSeR: azobenzene seleno-rosamine-based dye; NTR: Nitroreductases; DiBDP: dimeric BODIPY.

**Table 27:** Summary of publications about the use of compounds hypoxia environment-accumulated for PDT hypoxia-dependent.

Ref	Application	PS	Energy of Excitation	Type of ROS	In Vitro	In Vivo
[226]	PDT	EtNBS	EtNBS-PDT: 652 nm, 15 J∙cm^−2^, 25 to 300 mW∙cm^−2^ Hypoxia EtNBS-PDT: 670 nm, 100 mW∙cm^−2^, 5 to 20 J∙cm^−2^	nd	3D adherent OVCAR-5 human OvCa model	No
[227]	PDT	Ru(C-bpy)_2_/mLDH	520 nm, 100 mW∙cm^−2^, 8 min	^1^O_2_	HeLa cell line	HeLa tumour-bearing mice

nd: not determined.

**Table 28:** Summary of publications about fractional PDT.

Ref	Application	PS	Excitation Wavelength	Type de ROS	In Vitro	In Vivo
[229]	Continuous vs. fractional PDT	Photofrin and mTHPC	Interstitial: 628±3 nm for Photofrin and 652±3 nm for mTHPC Superficial: 100 mW∙cm^−2^	nd	No	RIF1 tumour-bearing mice
[230,231]	Continuous vs. fractional PDT	Radachlorin	20 mW∙cm^−2^ Pulse mode: 200 ms – pulse duration, 700 ms - period	^1^O_2_	k562 cell line	No
[232]	Continuous vs. fractional PDT	PAD-S31	Pulsed: 670 nm nanosecond pulsed Nd:YAG laser, peak fluence rate 1 MW∙cm^−2^, 30 Hz CW: 670 nm, 40 J∙cm^−2^, 180 mW∙cm^−2^ or 270 mW∙cm^−2^	nd	Renca cell line	No
[233]	Fractional PDT	Pyridone	655 nm, 324 µmol∙m^−2^∙s^−1^ photon flux for 10 min every 1h	^1^O_2_	HeLa cell line	No

nd: not determined; mTHPC: meta-tetrahydroxyphenylchlorin.
